# Supplementary material for: Predictors and prognosis of population-based subjective cognitive decline: longitudinal evidence from the Caerphilly Prospective Study (CaPS)
Source: BMJ Open. 2023 Oct 16;13(10):e073205. doi: 10.1136/bmjopen-2023-073205 (PMC10582873; doi:10.1136/bmjopen-2023-073205)
Supplement: Supplementary data [file bmjopen-2023-073205supp001.pdf]

Supplemental material for "Predictors and prognosis of population-based subjective cognitive decline: Longitudinal evidence from the Caerphilly Prospective Study (CaPS)"

Contents

Items derived from The Wisconsin Sleep Scale 1

Supplemental Figure 1: Caerphilly Prospective Study (CaPS) flowchart 2

Supplemental Figure 2: Histogram displaying delayed recall minus immediate recall ("internal inconsistency") at phase 5, according to presence or absence of simple Subjective Cognitive Decline (sSCD) 3

Supplemental Table 1: Relative frequency of possible risk variables, amongst people with and without simple Subjective Cognitive Decline (sSCD) (Phase 5) 4

Supplemental Table 2: Missingness by variable, as a percentage of n=1225 participants at phase 5 6

Supplemental table 3: possible risk factors (phases 2 to 4) for simple Subjective Cognitive Decline (phase 5), including unadjusted and imputed models 8

Items derived from The Wisconsin Sleep Scale

The Wisconsin sleep scale is mainly composed of items scored on a 1-5 point scale. These items were separated into two groups, then summed and standardized: i) *Experiencing poor sleep*, including: difficulty initiating sleep; waking overnight; difficulty getting back to sleep; not feeling rested during the day; daytime sleepiness; dozing easily in the daytime; use of caffeine or other stimulants to stay awake; and napping during the day. This comprised 8 items, (alpha 0.77). Minimum and maximum scores prior to standardisation were 8 and 39. ii) Breathing-disordered Sleep, including: frequency of snoring; loudness of snoring; awareness of snoring; making gasp/ choke/ snort noises during sleep; waking with these noises; and frequency of momentary periods in sleep where you breathe abnormally. This comprised 6 items (alpha 0.77). Minimum and maximum scores prior to standardisation were 6 and 30. Note this approach excluded items on the Wisconsin questionnaire that were either scored on a substantially different scale, or assessing other aspects of sleep: how awake you feel today; restless legs/ twitches/ kicking during sleep; nasal congestion interrupting sleep; sleep latency; reported duration of sleep (weekdays and weekends); use of sleeping tablets; and allergies causing nasal congestion.

Supplemental Figure 1: Caerphilly Prospective Study (CaPS) flowchart

References: (1, 2, 3)

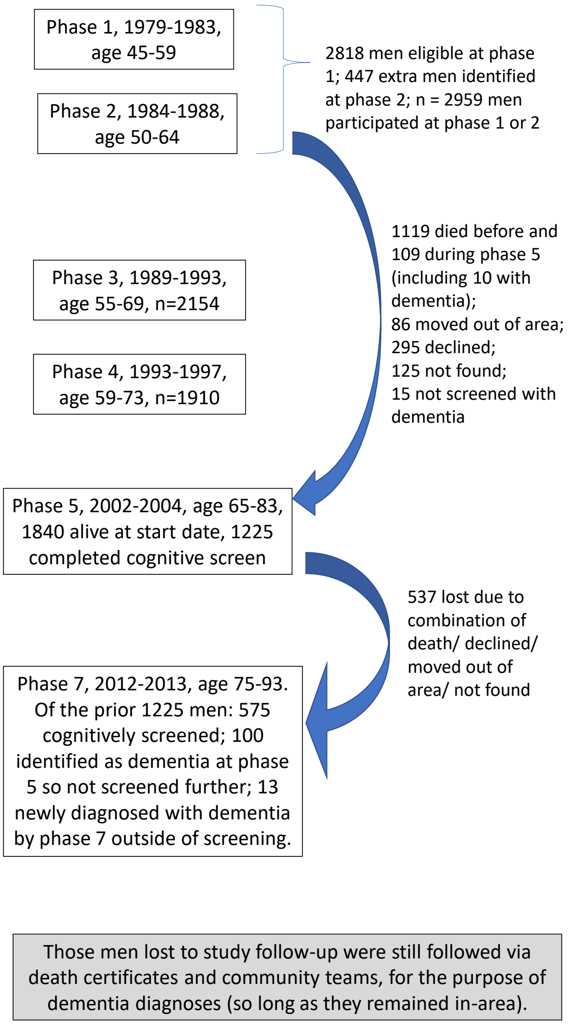

References:

1. Fish M, Bayer AJ, Gallacher JE, Bell T, Pickering J, Pedro S, et al. Prevalence and pattern of cognitive impairment in a community cohort of men in South Wales: methodology and findings from the Caerphilly Prospective Study. Neuroepidemiology. 2008;30(1):25-33.
2. Creavin ST, Gallacher J, Bayer A, Fish M, Ebrahim S, Ben-Shlomo Y. Metabolic syndrome, diabetes, poor cognition, and dementia in the Caerphilly prospective study. J Alzheimers Dis. 2012;28(4):931-9.
3. Elwood PC, Pickering J, Bayer A, Gallacher JEJ. Vascular disease and cognitive function in older men in the Caerphilly cohort. Age and Ageing. 2002;31(1):43-8.

Supplemental Figure 2: Histogram displaying delayed recall minus immediate recall ("internal inconsistency") at phase 5, according to presence or absence of simple Subjective Cognitive Decline (sSCD)

This analysis included only those men with no objective decline in measured cognition since Phase 3.

Score greater than 0 is "inconsistent". Note how roughly one fifth of men had this marker of internal inconsistency (regardless of subjective decline status).

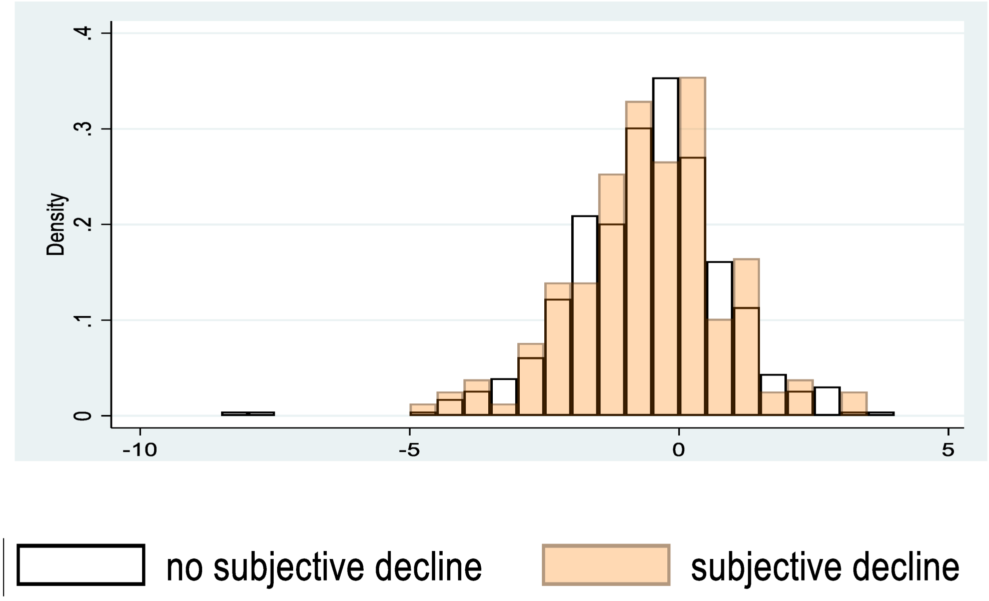

Supplemental Table 1: Relative frequency of possible risk variables, amongst people with and without simple Subjective Cognitive Decline (sSCD) (Phase 5)

	TOTAL N	NO SUBJECTIVE COGNITIVE DECLINE, N	SUBJECTIVE COGNITIVE DECLINE, N
TOTAL: N	1225	739	326
CIND (PHASE 5)	192	79	71
DEMENTIA (PHASE 5)	75	19	31
MANUAL SOCIAL CLASS	720	417	192
LEFT SCHOOL BEFORE 14	507	286	149
TEETOTAL (AT ANY PHASE 3-5)	120	63	37
CUMULATIVE HIGH ALCOHOL INTAKE	182	110	47
CURRENT SMOKER (ANY OF PHASE 3-5)	452	261	122
PROBABLE ISCHAEMIC HEART DISEASE	253	131	84
ANTIDEPRESSANT MEDICATION (BINARY)	15	6	8
	ALL	NO SUBJECTIVE COGNITIVE DECLINE, MEAN (SD)	SUBJECTIVE COGNITIVE DECLINE, MEAN (SD)
AGE (YEARS)	73.0 (4.2)	72.5 (4.0)	73.6 (4.2)
PREMORBID IQ	107.1 (9.9)	108.0 (9.9)	107.0 (9.7)
BODY MASS INDEX	26.9 (3.5)	27.0 (3.5)	26.8 (3.3)
WAIST-HIP RATIO	0.93 (0.06)	0.93 (0.06)	0.93 (0.06)
NUMBER OF MEDICATIONS FOR VASCULAR RISK	0.40 (0.83)	0.32 (0.71)	0.54 (1.05)
EXPERIENCING POOR SLEEP	-0.05 (0.95)	-0.23 (0.87)	0.33 (0.97)
BREATHING-DISORDERED SLEEP	0.02 (0.93)	-0.05 (0.88)	0.22 (0.98)
GHQ (MOOD) CHRONIC SCORING	9.0 (6.3)	7.8 (5.7)	11.3 (6.7)
STAI (TRAIT ANXIETY)	35.9 (8.9)	34.3 (8.5)	38.8 (9.3)
CAMCOG RATE OF CHANGE PER YEAR	-0.29 (0.96)	-0.17 (0.63)	-0.35 (0.91)

**AH4 RATE OF CHANGE PER YEAR**	-0.15 (0.64)	-0.08 (0.61)	-0.26 (0.66)

CIND = cognitive impairment, not depression; QGH = general health questionnaire (a measure of mood symptoms); STAI = Spielberger State-Trait Anxiety Inventory; CAMCOG = Cambridge Cognitive Examination; AH4= Alice Heim 4 test. *For missingness data, see Supplementary Table 1.*

Note where "no subjective cognitive decline" and "subjective cognitive decline" columns do not add up to the total, this is due to missing data. This data was missing 160/1225 (13%) for the whole cohort, 42/192 (22%) for CIND and 25/75 (33%) for dementia.

Supplemental Table 2: Missingness by variable, as a percentage of n=1225 participants at phase 5

VARIABLE	PHASE OF DATA COLLECTION	% MISSING
Age	Baseline	(none)
Sex	Baseline	(none, all men)
Manual social class	Baseline	4.9
Left school at 14	Baseline	10.3
STAI	2	5.3
Probable ischaemic heart disease	3	5.6
Antidepressant medication (binary)	3	Missingness data not available
Premorbid IQ	3	12.1
Body mass index	3	10.5
Waist hip ratio	3	8.2
Experiencing poor sleep	3	9.1
Sleep-disordered breathing	3	34.0
Mood symptoms (GHQ)	3	17.8
CAMCOG	3	11.6
AH4	3	12.2
CAMCOG	4	15.4
AHR	4	16.2
Teetotal	3-5	4.8
Cumulative high alcohol intake	3-5	4.8
Smoker	3-5	4.7
Number of medications for vascular risk	3	Missingness data not available
Subjective Cognitive Decline	5	13.1
CAMCOG	5	0.4
AH4	5	6.9
Immediate recall	5	2.5
Delayed recall	5	2.5
CIND or dementia	5	0.2
CAMCOG (excluding men who had dementia by phase 5)	7	56.0

CIND or dementia (excluding men who had dementia by phase 5)	7	53.0

Supplemental table 3: possible risk factors (phases 2 to 4) for simple Subjective Cognitive Decline (phase 5), including unadjusted and imputed models

	n FOR UNADJUSTED OR	UNADJUSTED OR (95% CI)	ADJUSTED OR (95% CI) (n=601)	ADJUSTED OR (95% CI), MICE (N=1225)	ADJUSTED OR (95% CI) EXCLUDING DEMENTIA (N=580)
**AGE (YEARS)**	1065	1.07 (1.03, 1.10)	1.12 (1.06, 1.17)	1.08 (1.04-1.12)	1.11 (1.05, 1.17)
**SOCIAL CLASS (MANUAL/NON-MANUAL)**	1020	1.10 (0.84, 1.45)	0.99 (0.65, 1.51)	1.07 (0.75-1.51)	0.95 (0.62, 1.46)
**PREMORBID IQ (X 0.1)**	956	0.90 (0.78, 1.03)	1.06 (0.84, 1.33)	1.09 (0.90-1.32)	1.05 (0.83, 1.32)
**HIGH ALCOHOL USE (BINARY)**	1022	0.96 (0.66, 1.39)	1.07 (0.63, 1.83)	1.08 (0.71-1.64)	1.20 (0.70, 2.07)
**TEETOTAL (BINARY)**	1022	1.37 (0.89, 2.11)	0.64 (0.31, 1.32)	1.14 (0.69-1.90)	0.60 (0.29, 1.27)
**PROBABLE ISCHAEMIC HEART DISEASE (BINARY)**	1015	1.62 (1.18, 2.22)	0.98 (0.59, 1.62)	0.94 (0.65-1.36)	1.03 (0.61, 1.72)
**NUMBER OF VASCULAR MEDICATIONS**	1065	1.33 (1.15, 1.55)	1.18 (0.92, 1.50)	1.10 (0.92-1.31)	1.20 (0.93, 1.54)
**SMOKING (BINARY)**	1022	1.10 (0.83, 1.44)	0.89 (0.60, 1.34)	1.10 (0.80-1.51)	0.85 (0.56, 1.30)
**WAIST-HIP RATIO (X10)**	990	1.19 (0.94, 1.50)	0.93 (0.66, 1.31)	1.07 (0.84-1.38)	0.89 (0.63, 1.27)
**POOR SLEEP**	983	1.91 (1.64, 2.22)	1.52 (1.19, 1.94)	1.48 (1.22-1.79)	1.55 (1.21, 1.99)
**SLEEP-DISORDERED BREATHING**	727	1.39 (1.17, 1.65)	1.13 (0.91, 1.40)	1.16 (0.95-1.42)	1.12 (0.90, 1.40)
**MOOD SYMPTOMS (GHQ SCORE: CHRONIC)***	886	1.78 (1.53, 2.06)	1.27 (1.01, 1.59)	1.38 (1.13-1.69)	1.23 (0.97, 1.55)
**TRAIT ANXIETY (STAI)***	1019	1.66 (1.44, 1.91)	1.38 (1.09, 1.74)	1.31 (1.10-1.57)	1.39 (1.09, 1.76)
**RATE OF DECLINE CAMCOG**	969	1.37 (1.13, 1.66)	1.18 (0.83, 1.69)	1.15 (0.95-1.40)	1.18 (0.72, 1.95)
**RATE OF DECLINE AH4**	925	1.59 (1.26, 1.99)	1.33 (0.95, 1.85)	1.50 (1.16-1.95)	1.36 (0.97, 1.92)

MICE = multiple imputation with chained equations
