# Supplementary material for: Predictors and prognosis of population-based subjective cognitive decline: longitudinal evidence from the Caerphilly Prospective Study (CaPS)
Source: BMJ Open. 2023 Oct 16;13(10):e073205. doi: 10.1136/bmjopen-2023-073205 (PMC10582873; doi:10.1136/bmjopen-2023-073205)
Supplement: Author's manuscript [file bmjopen-2023-073205.draft_revisions.pdf]

BMJ Open is committed to open peer review. As part of this commitment we make the peer review history of every article we publish publicly available.

When an article is published we post the peer reviewers' comments and the authors' responses online. We also post the versions of the paper that were used during peer review. These are the versions that the peer review comments apply to.

The versions of the paper that follow are the versions that were submitted during the peer review process. They are not the versions of record or the final published versions. They should not be cited or distributed as the published version of this manuscript.

BMJ Open is an open access journal and the full, final, typeset and author-corrected version of record of the manuscript is available on our site with no access controls, subscription charges or pay-per-view fees (http://bmjopen.bmj.com).

If you have any questions on BMJ Open's open peer review process please email info.bmjopen@bmj.com

BMJ Open

Predictors and prognosis of population-based subjective cognitive decline: Longitudinal evidence from the Caerphilly Prospective Study (CaPS)

Journal:	*BMJ Open*
Manuscript ID	bmjopen-2023-073205
Article Type:	Original research
Date Submitted by the Author:	02-Mar-2023
Complete List of Authors:	Ball, Harriet; University of Bristol, Population Health Sciences<br>Coulthard , Liz ; University of Bristol Medical School, Translational Health Sciences<br>Fish, Mark; Royal Devon University Healthcare NHS Foundation Trust<br>Bayer, Tony; Cardiff University, Institute of Primary Care and Public Health<br>Gallacher, John; Oxford<br>Ben-Shlomo, Yoav; University of Bristol, Population Health Sciences
Keywords:	Dementia < NEUROLOGY, Dementia, Delirium & cognitive disorders < PSYCHIATRY, EPIDEMIOLOGY

SCHOLARONE™
Manuscripts

I, the Submitting Author has the right to grant and does grant on behalf of all authors of the Work (as defined in the below author licence), an exclusive licence and/or a non-exclusive licence for contributions from authors who are: i) UK Crown employees; ii) where BMJ has agreed a CC-BY licence shall apply, and/or iii) in accordance with the terms applicable for US Federal Government officers or employees acting as part of their official duties; on a worldwide, perpetual, irrevocable, royalty-free basis to BMJ Publishing Group Ltd ("BMJ") its licensees and where the relevant Journal is co-owned by BMJ to the co-owners of the Journal, to publish the Work in this journal and any other BMJ products and to exploit all rights, as set out in our licence.

The Submitting Author accepts and understands that any supply made under these terms is made by BMJ to the Submitting Author unless you are acting as an employee on behalf of your employer or a postgraduate student of an affiliated institution which is paying any applicable article publishing charge ("APC") for Open Access articles. Where the Submitting Author wishes to make the Work available on an Open Access basis (and intends to pay the relevant APC), the terms of reuse of such Open Access shall be governed by a Creative Commons licence – details of these licences and which Creative Commons licence will apply to this Work are set out in our licence referred to above.

Other than as permitted in any relevant BMJ Author's Self Archiving Policies, I confirm this Work has not been accepted for publication elsewhere, is not being considered for publication elsewhere and does not duplicate material already published. I confirm all authors consent to publication of this Work and authorise the granting of this licence.

For peer review only

Predictors and prognosis of population-based subjective cognitive decline: Longitudinal evidence from the Caerphilly Prospective Study (CaPS)

Harriet Ball[1], Elizabeth Coulthard[2], Mark Fish[3], Antony Bayer[4], John Gallacher[5], Yoav Ben-Shlomo[1].

1. Population Health Sciences, Bristol Medical School, University of Bristol, UK
2. Translational Health Sciences, Bristol Medical School, University of Bristol, UK
3. Royal Devon University Healthcare Trust, Exeter, UK
4. Division of Population Medicine, Cardiff Medical School, Cardiff University, UK
5. Department of Psychiatry, University of Oxford, UK

Corresponding author: Harriet Ball, harriet.ball@bristol.ac.uk Southmead Hospital Learning and Research Building, Bristol Medical School, University of Bristol.

Word count: 4,077

For peer review only

For peer review only - http://bmjopen.bmj.com/site/about/guidelines.xhtml

Article summary

Objectives: to unpick associations between the subjective experience of cognitive decline, and objective cognition. This subjective experience is often conceptualised as an early step towards neurodegeneration, but this has not been scrutinised at the population level. An alternative explanation is poor meta-cognition, the extreme of which is seen in Functional Cognitive Disorder (FCD).

Design: prospective cohort (Caerphilly Prospective Study)

Setting: population-based, South Wales, UK

Participants: This study began in 1979; 1225 men participated at average age 73 in 2002-2004, including assessment of simple Subjective Cognitive Decline (sSCD, defined as subjective report of worsening memory or concentration). Dementia outcomes were followed up to 2012-2014. Data on non-completers was additionally obtained from death certificates and local health records.

Primary and Secondary Outcome Measures: The primary outcome measure was incident dementia in the final 10 years. Secondary outcome measures included prospective change in objective cognition, and cross-sectional cognitive internal inconsistency (the existence of a cognitive ability at some times, and its absence at other times, with no intervening explanatory factors except for focus of attention).

Results: sSCD was common (30%) and only weakly associated with prior objective cognitive decline (sensitivity 36% [95% CI 30-42], specificity 72% [95% CI 68-75]). Independent predictors of sSCD were older age, poor sleep quality and higher trait anxiety. Those with sSCD did not have excess cognitive internal inconsistency, but results suggested a mild attentional deficit. sSCD did not predict future objective cognitive change (linear regression coefficient -0.01 (95% CI -0.13, 0.15)).

Conclusions: sSCD is weakly associated with prior objective cognitive decline, and does not predict future cognition. Prior sleep difficulties and anxiety were the most robust predictors of sSCD. sSCD in

For peer review only - http://bmjopen.bmj.com/site/about/guidelines.xhtml

For peer review only

the absence of objective decline appears to be a highly prevalent example of poor meta-cognition (i.e., poor self-awareness of cognitive performance), which could be a driver for later FCD.

Strengths and limitations of this study

- The design was prospective and population-based, with detailed collection of many potential risk or confounding factors, from middle to older age
- Outcome measures included objective test scores, and clinical dementia diagnoses (including local clinic and death certificate records for those who did not participate to the end)
- The study only included men from the outset, so we cannot extrapolate results to women

Funding: CaPS was funded by the Medical Research Council (grant G9824960-E01/1), and phase 5 was funded by a grant from the Alzheimer's Society.

Competing interests: None of the authors declare any competing interests.

Keywords: Dementia, subjective cognitive decline, functional cognitive disorder

For peer review only - http://bmjopen.bmj.com/site/about/guidelines.xhtml

BACKGROUND

We know that cognitive symptoms are common, and many people who attend memory clinics due to cognitive symptoms turn out not to have a progressive cognitive disorder.(1) The link between subjective reports and objective evidence of cognitive decline is surprisingly weak,(2, 3, 4) or sometimes found only in subgroups. An international working group (SCD-I) has published criteria for "pre-MCI Subjective Cognitive Decline" as those who experience persistent decline in cognitive ability relative to their baseline; despite scoring in normal ranges on objective cognitive tests; and excluding those meeting criteria for Mild Cognitive Impairment (MCI) or dementia, that explained by psychiatric or neurologic disease (except AD), medical disorder, medication or substance abuse.(5) This definition has been created to harmonise research exploring evidence of neurodegeneration. However, this definition excludes many in the population with new-onset subjective experience of cognitive decline (which is important since this experience, regardless of the exclusions in the definition, drives their concern and presentation to medical services). We define *simple* subjective cognitive decline (sSCD) as purely the subjective experience, irrespective of objective cognition or medical or psychiatric disorders.

A multi-cohort study found the risk of dementia in those with SCD (as defined by the SCD-I) was driven very largely by clinic-based rather than community or population-based samples.(6) Similarly, population-level association between SCD and amyloid status is masked once controlling for anxiety and demographic variables.(7) In the English Longitudinal Study of Ageing (ELSA) cohort,(8) although objective cognitive changes predicted subsequent worsening in subjective cognition and depression, deterioration in subjective cognition did not prospectively predict either depression or objective cognition. Therefore, isolated subjective cognitive problems *often do not* herald the start of a progressive neurodegenerative process. Research attention is now focusing on a narrower group who have extra clinical features ("SCD-plus"), but who are yet further from the common population experience of subjective worsening in cognitive ability. "SCD-plus" refers to Subjective Cognitive

For peer review only - http://bmjopen.bmj.com/site/about/guidelines.xhtml

Decline plus at least one additional feature including: amnestic rather than non-amnestic complaints; onset <5 years ago; onset >age 60 years; concern associated with SCD; persistence of SCD over time; help-seeking; confirmation of decline by an informant).(9) “SCD plus” has been associated with a higher risk of developing objective cognitive deficits,(10) or frank dementia, than those with SCD alone.(11)

The aim of this paper is to explore population-based associations with, and predictions from, the subjective experience of cognitive decline (sSCD), since outcomes are likely to be different than when examining clinic-based samples with exclusions making them less representative. The latter group likely have worse outcomes, which might make more aggressive investigation and potential treatment appropriate, but this may not hold for the whole population with subjective cognitive problems. This paper also takes a more nuanced and dimensional view of any association with objective cognitive change, noting individual differences in test scores rather than whether someone is just above versus just below a cutoff for “abnormal”.

A subjective experience of cognitive difficulty that outstrips objective evidence, when severe and impairing, is a key feature of Functional Cognitive Disorder(12); but a milder experience might be common in the wider population. Functional symptoms (which can wax and wane) therefore might explain *some* of the “recovery” of people with scores in the Mild Cognitive Impairment range to the normal range (up to one third in community based samples [13]).

Population-based analyses show subjective cognitive concerns are more common in people who are older, of lower social class and less educated[14]; and are associated with worse quality of life, and higher anxiety[15]. Sleep disturbance is associated with objective cognitive dysfunction, more so at older ages[16], but less has been reported about its association with subjective cognition.

For peer review only - http://bmjopen.bmj.com/site/about/guidelines.xhtml

For peer review only

The current paper uses a prospective cohort of men in Wales, with in-depth cognitive assessments, and data spanning over three decades, to examine the prevalence of sSCD, its relation to objective cognitive change over time, and prospective associations with potential causative factors. We hypothesized that (i) sSCD is a poor indicator of objective test score, and some men with sSCD will show features of cognitive internal inconsistency. (ii) sSCD has different risk factors compared to objective cognitive decline, related to psychological processes around attention paid to normal cognitive slips. (iii) men with sSCD do not have an elevated risk of future objective cognitive decline or dementia.

METHODS

The Caerphilly Prospective Study (CaPS) began in 1979 (Supplemental Figure 1). Men aged 45-59 were identified from electoral rolls and general practice lists in Caerphilly and surrounding villages in South Wales, UK. The initial focus was on cardiovascular health, but later Phases (beginning in phase 3, age 55-69) added cognitive assessments including the Cambridge Cognitive Examination (CAMCOG)[17], the Alice Heim 4 test (AH4)[18] and components of the Rivermead Behavioural Memory Test (RBMT,[19] which includes immediate recall of a 21-clause story, and delayed recall after 20 minutes). Cognitive assessments were also repeated at Phases 4 and 5. At Phase 5 (mean age 73 years), a subset of participants (those who could not complete the CAMCOG, or scored 82 or fewer, or showed at least a 10 point decline over time) were invited to a clinical assessment to identify those who had dementia, and those who had "cognitive impairment not dementia" (CIND, which includes those with MCI). Informants (e.g., spouses or children) also rated change in participants' day to day abilities (IQCODE(20)). A further assessment of *likely* dementia status was made holistically at Phase 7, by a cognitive disorders clinician (AB), using all available information, including longitudinal CAMCOG score, IQCODE, and all medical records. Those who had previously been classified as having dementia at Phase 5 were assumed to still have this diagnosis and were not re-evaluated. By this Phase (mean age 82 years), some men had other health problems making it

For peer review only - http://bmjopen.bmj.com/site/about/guidelines.xhtml

difficult to complete the cognitive assessments, leading to altered scores related to factors such as hearing, vision or manual dexterity. We have followed the STROBE cohort reporting guidelines.(21) In assessing decline in objective cognitive test scores from Phases 3 to 5, we focused firstly on an instrument typically used as a dementia screen (CAMCOG), and secondly a scale more often used to indicate reasoning skills in the general population (AH4). The mean score at Phases 3 and 4 (or just one if the other was missing), was subtracted from the score at Phase 5, and divided by the time interval, to indicate rate of change over time. We defined “objective cognitive decline” as: a decline of one standard deviation below the mean decline, of those men who had neither dementia nor CIND at Phase 5. While arguably an arbitrary cutoff, this value was chosen as a real-world approach to capture a difference from an expected average decline over 8-12 years.

At Phase 5, men reported on their subjective cognition via two items (*change in memory*, and *change in concentration*, over the past 12 months). Each was recorded on a rating scale; we re-coded responses indicating improvement to zero, to focus on decline over time (0-2). Scores on these two items were correlated (Spearman rho 0.64, p<0.001) and we combined them to generate a total score (0-4). A binary variable (sSCD) was defined as positive for participants scoring 2 or more out of 4. Therefore our definition of SCD is more basic than SCD as defined earlier by the international working group.

“Cognitive internal inconsistency” was not a focus of this study at the time of data collection. Performance validity tests exist, but are protracted and were not performed as part of this study; further, the literature around these tests mainly focus on identification of conscious malingering, eg the TOMM.(22) We therefore looked for ways to identify unambiguous cognitive internal inconsistency in routinely collected data. We pragmatically operationalised this as delayed recall being paradoxically higher than immediate recall on the RBMT story recall task, and compared the frequency of this across those with and without sSCD.

For peer review only - http://bmjopen.bmj.com/site/about/guidelines.xhtml

We considered the following variables as important confounders: age, social class (manual vs non-manual), years of education (before or after age 14 years) and premorbid IQ generated via the National Adult Reading Test (NART)[23]. We assessed neurodegenerative risk markers at Phases 2 to 4. These included probable ischaemic heart disease (assessed through hospital records and ECG changes), BMI and waist-hip ratio, smoking status, and medication history indicating vascular risk (i.e., medications whose indications were unequivocally for blood pressure, vascular disease, diabetes, or lipid-lowering). Alcohol exposure was indexed both as a binary marker of avoidance ("teetotal" at *any* of Phases 2 through 4), and a binary indicator of the highest cumulative alcohol use at Phases 2 through 4 (those drinking an average of 4 or more units per day across the period), due to previous work suggesting a non-linear association between alcohol and cognition[24]. At Phase 3 we also assessed mood symptoms, using the chronic scoring of the General Health Questionnaire, the Spielberger State-Trait Anxiety Inventory, and use of any anti-depressant medication. Sleep problems were assessed using items from the Wisconsin Sleep Questionnaire, divided into "experiencing poor sleep" and "breathing-disordered sleep" (see Supplemental material).

Statistical analysis plan

We examined the prevalence of our main outcome measure, sSCD and its association with potential confounders. We examined the relationship between sSCD and objective cognitive decline, the latter assessed both as a continuous score, and a binary measure "objective cognitive decline" (on CAMCOG and AH4 separately). We looked for an association between sSCD and cognitive internal inconsistency, using a chi-squared test. We ran multivariable logistic regression models to identify possible causal contributors to sSCD, and used linear regression to test whether sSCD predicted a decline in CAMCOG between Phases 5 and 7. To address potential survivor bias, we included multiple imputation analysis with chained equations (MICE) as well as complete case analysis, when looking at Phase 7 outcomes.

For peer review only - http://bmjopen.bmj.com/site/about/guidelines.xhtml

For peer review only

As sensitivity analyses, we repeated the above excluding those with a pre-existing diagnosis of dementia. This is because in a clinical setting, it is usually relatively straightforward to identify people whose cognitive impairment is severe enough to be categorised as dementia; the diagnostic difficulty lies in understanding causes amongst those whose cognitive difficulty is milder. We also repeated analyses looking at the memory-only versus concentration-only aspects of subjectively reported cognition.

Patient and public involvement

This study began in the 1970s when it was not normal practice to involve participants in the design and co-production of research. However, the researchers kept the participants well informed of the research progress and key findings through regular newsletters and including a 25th follow-up birthday party where all survivors met the research team and the Welsh Minister of Health. Over the study duration, ad hoc feedback has occurred with study participants writing or speaking to the research team with their views about the study. For example, a sensitive question was dropped based on participants' comments.

RESULTS

Sociodemographics, and frequency of risk variables

1,225 men took part at Phase 5 including completion of cognitive assessments, with mean age of 73 (range 65-83) years (Supplemental Figure 1). 62% of men (n=720) were of manual social class. 46% (n=507) had left school by age 14 (in later analyses, we use NART-IQ as a more normally distributed proxy for amount of education received). 75 (6.1%) were assessed as having dementia, and 192 (15.7%) as having CIND. 326 participants (30.6%) had sSCD. Unadjusted, subjects with sSCD were more likely to have CIND, Dementia, lower education, probable IHD, be older, report worse sleep, have a worse GHQ score, and show slightly greater declines in their CAMCOG and AH4 scores (Supplemental Table 1).

For peer review only - http://bmjopen.bmj.com/site/about/guidelines.xhtml

Only 15 men (1.2%) were on antidepressant medication; due to low prevalence, this variable was not analysed further.

Does objective cognitive decline predict subjective cognitive decline?

Objective CAMCOG cognitive change was scored continuously, and the average drop (from the baseline of Phase 3 or 4, to Phase 5) was 2.7 points per year in those developing dementia, 0.7 per year in those developing CIND, and 0.06 per year in those without either by Phase 5. The equivalent figures for AH4 were 0.9, 0.2 and 0.1 respectively. Objective changes in cognition were mildly worse in those with sSCD (Table 1), but the proportion of variance explained was very low. These associations were reduced when excluding those with dementia, although this (unadjusted) association remained statistically significant for AH4 (but not for CAMCOG).

Of people who reported sSCD, the majority did not have objective cognitive decline as defined by >=1 standard deviation decline (Table 2). Therefore the use of sSCD as a simple diagnostic screening tool for objective decline would be very inaccurate, since its sensitivity varied from 36 to 44% (lower if participants with dementia were excluded). Hence the majority of individuals are missed (> 50% false negative rate). The specificity of sSCD was consistently higher (between 72 to 75%) but still insufficiently high to use as a rule-in test, since almost a quarter of subjects are “false positives”.

Do those with subjective cognitive decline, but no objective decline, have cognitive internal inconsistency?

In the Rivermead story recall test, subjects reporting sSCD did slightly worse than others on immediate recall, and then proportionately worse on delayed recall (i.e., they scored lower on delayed recall, but this was in proportion to the items missed on immediate recall, which is more reflective of a mild attentional deficit than a true amnestic difficulty). There was no evidence of

For peer review only - http://bmjopen.bmj.com/site/about/guidelines.xhtml

excess cognitive internal inconsistency (indicated by a higher score on delayed than immediate recall) when comparing those with and without sSCD. (Table 3 and Supplemental Figure 2).

What predicts subjective cognitive decline?

Variables that predicted sSCD in unadjusted logistic models included: age; probable ischaemic heart disease; vascular medications; experiencing poor sleep; breathing-disordered sleep; mood symptoms; trait anxiety; rate of decline in CAMCOG; and rate of decline in AH4 (Table 4). There was no unadjusted association with social class, premorbid IQ, alcohol use (either high use or teetotal status), tobacco exposure, BMI, waist-hip ratio, nor baseline measures of CAMCOG and AH4. However, in a model simultaneously adjusting for all these factors, the only significant independent predictors remaining were: age, experiencing poor sleep, mood symptoms, and trait anxiety. These associations (except mood symptoms, which became borderline non-significant) remained when excluding participants with a clinical diagnosis of dementia at Phase 5.

Can subjective cognitive decline predict future CIND and dementia?

Of those who did not already have dementia at Phase 5, 37/573 (6.5%) had dementia by Phase 7. Of those who did not already have CIND, 84/528 (15.9%) had CIND or dementia by Phase 7. Of those with a cognitive functional classification excluding dementia at Phase 5, 575/1148 (50.1%) had missing data on this measure at Phase 7.

sSCD at Phase 5 did not predict worsening cognition at Phase 7 (Table 5), when examined either by category (new CIND or new dementia), or by change in CAMCOG score. Due to attrition to Phase 7, this was assessed both by complete case analysis and multiple imputation with chained equations (MICE). There was a potential trend towards an interaction between sSCD and CAMCOG score at Phase 5, but this was weakened and consistent with chance in the MICE analysis.

For peer review only - http://bmjopen.bmj.com/site/about/guidelines.xhtml

Sensitivity analyses

We replaced overall sSCD with either of memory-only or concentration-only subjective report indicators. These showed the same patterns of association with measures of objective cognition, internal inconsistency (lack of association), possible confounders and risk factors, and future dementia (lack of association).

DISCUSSION

This is the first large population-based cohort study of subjective experience of cognitive decline which can look at both predictors as well as future clinical outcomes. In addition, we were able to explore associations with cognitive internal inconsistency (which is hypothesised to contribute to functional neurological symptoms). In this population-based sample of 1,225 older men in South Wales, we found only a weak association between objective cognitive change, and subjective report of that same change. Those who had subjective cognitive concerns without objective cognitive decline, nonetheless, showed a mild deficit in attentional processes. At the population level, sSCD has low sensitivity and specificity to identify objective evidence of cognitive decline (both missing many people who lack insight into their cognitive problems; and detecting people who are concerned in the absence of objective problems). Independent predictors for sSCD included preceding anxiety or mood symptoms, and poor self-reported sleep, as well as older age, but not other variables known to be risk factors for neurodegeneration. sSCD, adjusted for objective cognition, did not predict later CIND or dementia.

There are various explanations for the weak association between subjective and objective cognition, including that population-based participants are substantially different to those in clinics (upon whom many previous studies have been based). Most clinic-based studies of SCD often have participants with "SCD-plus" (i.e., extra factors, such as partner concern, that make neurodegeneration more likely and may increase their chances of reaching clinic). The choice of test

For peer review only - http://bmjopen.bmj.com/site/about/guidelines.xhtml

For peer review only

and cut-off, and cross-sectional versus decline measure, can also affect results. Our decline measure is potentially a quite sensitive measure of change over time, which could therefore be less prone to measurement error than a one-off test, and also controls for differences in baseline ability. The exact question framing also matters regarding self-reported cognitive difficulties, since some items are more likely to index normal ageing, and others pathological changes.[25, 26, 27]

Of our two objective cognitive tests, AH4 showed a slightly more robust association with sSCD than did CAMCOG (AH4 assesses complex verbal and numerical reasoning, analogous to an IQ test; CAMCOG measures faculties more commonly assessed in the dementia clinic). Perhaps the population variance in complex reasoning more closely mirrors peoples' understanding of what it is to have memory or concentration problems. Alternatively, perhaps AH4 captures more meaningful variance in the normal range of cognition, whereas CAMCOG is better at detecting the more severe end (where those with cognitive difficulties often lack insight). Either way, it may be useful to incorporate measures similar to AH4 into neuropsychometry batteries for patients with suspected functional neurological symptoms, as this could represent a measure capable of tracking progress, providing a useful discussion point with patients.

Amongst men with subjective cognitive symptoms despite a lack of objective cognitive decline, we found they fared slightly worse (compared to those without cognitive symptoms) in a task of immediate recall, but no differently on delayed recall of the initially remembered items. This reinforces the suggestion that SCD is linked to a slight deficit in attentional processes (despite not having dipped on overall cognitive test scores over many years). This pattern is distinct from the amnestic processes typically seen in early Alzheimer's disease(28, 29) or the attentional problems alongside wider progressive cognitive problems seen in dementia with Lewy bodies[30]. It is also of note that this group did not exhibit cognitive internal inconsistency (no evidence of scoring *better* on delayed recall than on immediate recall). This is not entirely surprising, as our group are quite

For peer review only - http://bmjopen.bmj.com/site/about/guidelines.xhtml

distinct from clinic-based patients with Functional Cognitive Disorder in whom this characteristic is a key part of diagnosis(12) (and in whom more than a one-off example of evidence of internal inconsistency should be sought). Attentional problems have been identified in other conditions within the umbrella of functional somatic syndromes, when such patients have been tested cognitively (31). It is also important to recognise that our pragmatic measure of "cognitive internal inconsistency" is identifiable in approximately one fifth of normal healthy people (Supplemental Figure 2), a surprisingly high number if only taking this one snapshot measurement into account.

We found that sSCD was more common at older ages. The same has been found in individual studies where a broad age range is considered[8]. When comparing across studies of people of differing ages, this association is less robust(1), probably in part due to heterogeneity between included studies. Reasons for this association could include differences in meta-cognitive processes by age; older people resigning to using work-arounds; age-related cultural expectations; or the way questions are framed leading to self-rating against different comparator groups; as well as some loss of insight when objective cognitive deficits take over[32].

The dip in subjective cognition with age may reflect the recognised small dip in objective cognitive abilities at older ages, as well as higher prevalence of neurodegeneration. However, factors typically associated with neurodegenerative change (vascular health, alcohol use) did not predict later sSCD. Instead, predictors included prior reports of poor sleep quality, and prior anxiety symptoms. This supports the proposition that anxiety and poor sleep may be contributing to sSCD (rather than being a by-product of worry about cognitive change). These risk factors for sSCD may also predict functional cognitive symptoms. It is also notable that anxiety and poor sleep are relevant in our study which consisted of older males (whereas the literature and clinical diagnoses of functional disorders focus more on younger females).

For peer review only - http://bmjopen.bmj.com/site/about/guidelines.xhtml

Subjective reports of poor sleep are often reported alongside sSCD, but the opposite has been reported using actigraphy (an objective assessment of sleep quality)[1]. There are likely qualitatively different sleep problems amongst those with sSCD, compared to those with organic changes to sleep in early dementia.

Limitations

These data are observational, so we cannot draw strong causal inferences. CAMCOG represents a summation of different cognitive faculties; it would be helpful to be able to examine individual cognitive domains more carefully, to better understand which most closely track SCD; and similarly, the same cognitive faculty, under different levels of attentional focus (e.g., free recall vs recognition vs implicit memory). The nature of sSCD could also be probed in more detail, such as the level of concern associated with it.

This study examined older men only, in one region of Wales. We should be cautious to generalise our results to wider populations. This is not the demographic associated with the highest prevalence of FCD as reported from clinics. Nevertheless, ours is an important group to study, as we empirically don't know how common functional cognitive symptoms may be in the general population, especially since diagnostic labels may be swayed by the higher a priori likelihood of neurodegeneration at older ages.

Some results may be influenced by survival bias: only men who survived to Phase 5 (average age 73) are included; their cognitive trajectories are unrepresentative of men who died or became too impaired to fully take part. Nonetheless, this study gives greater insight than would a hypothetical study that only began enrolling men at an older age (and thus never included those men from the start). And, this study was further informed by examination of death certificates and local health records amongst those who did not take part to the end.

Conclusions and implications

For peer review only - http://bmjopen.bmj.com/site/about/guidelines.xhtml

Subjectively experiencing cognitive decline in the absence of an objective decline, is a marker of poor meta-cognition (i.e. poor ability to reflect on and monitor one's own cognitive abilities). This is a process postulated to be important in the development of FCD[33]. The current study demonstrates the high prevalence of subjective cognitive decline in the absence of objective cognitive decline. It is not linked to later neurodegeneration, and given its high prevalence, can at most be a partial contributor in the development of FCD. Since it is associated with increased self-focused attention[34], it may be a vulnerability point from which some people go on to develop FCD. Of course there are many alternative causes of cognitive change (aside from FCD and neurodegeneration), so holistic clinical assessment remains crucial for those with problematic symptoms.

Although low-mood symptoms, and reports of poor sleep which often go alongside low mood, were found to predict sSCD, this does *not* simply imply that depression causes SCD. Most of the people in our sample did not have symptoms sufficient to qualify for a clinical diagnosis of depression, rather these are correlations between milder level symptoms. Whilst depressive symptoms correlate primarily with subjective measures of cognition, they may[35] or may not[8] lead to objective cognitive deficits. This suggests that processes interfering with meta-cognition may affect those with depression, as well as many others without depression.

These findings are relevant to the current design of memory clinics, since any healthcare system would be overwhelmed if all people with sSCD were referred, with little need or benefit from clinical input (and potential iatrogenic harm via heightened concern whilst awaiting clinic assessment). It is therefore important to capture objective evidence of decline to identify those more likely to have early neurodegeneration. Importantly, patients with FCD assessed in tertiary centres often *do* have mild objective cognitive difficulties.(12, 36) Finally, our data provide reassuring evidence that

For peer review only - http://bmjopen.bmj.com/site/about/guidelines.xhtml

subjects with sSCD but without objective impairments, are not at increased risk of later CIND or dementia.

List of abbreviations:

FCD: Functional Cognitive Disorder

MCI: Mild Cognitive Impairment

SCD: Subjective Cognitive Decline

sSCD: simple Subjective Cognitive Decline (the subjective report of cognitive decline, with no other qualifiers such as score on objective tests or absence of comorbidities).

For peer review only

For peer review only - http://bmjopen.bmj.com/site/about/guidelines.xhtml

DECLARATIONS

Ethical approval and consent to participate

All participants gave informed consent. Ethical approval was provided by Gwent Research Ethics Committee (01/69).

Consent for publication

Not applicable.

Availability of data and materials

The datasets analysed during the current study are not publicly available due the sensitive information contained, and the nature of the consent given by the participants. The data that support the findings are available on reasonable request.

Competing interests

None of the authors declare any competing interests.

Funding

CaPS was funded by the Medical Research Council (grant G9824960-E01/1), and phase 5 was funded by a grant from the Alzheimer's Society.

Author Contributions

MF, AB, JG and YBS contributed to conception and design of the study. JG led the data collection. MF and AB established the clinical diagnoses. HB, YBS and EC planned the analyses. HB analysed the data and wrote the first draft. All authors read and approved the final manuscript.

Acknowledgements

HB is a National Institute for Health Research (NIHR) academic clinical lecturer. YBS is partially supported by the NIHR Applied Research Collaboration West (NIHR ARC West). JG is partially supported by MRC grant MR/T0333771. The views expressed in this article are those of the author(s) and not necessarily those of the NIHR or the Department of Health and Social Care.

For peer review only

For peer review only - http://bmjopen.bmj.com/site/about/guidelines.xhtml

Table 1: Relationship between change in objective cognition performance (Phase 3 to 5) and self-reported simple Subjective Cognitive Decline (sSCD) (phase 5)

	MEAN (SD)			P-VALUE	ADJUSTED R-SQUARED*
**DECLINE IN COGNITIVE TEST SCORE (ANNUAL RATE)**	No subjective cognitive decline	Subjective cognitive decline	Difference (95% CI)		
WHOLE SAMPLE					
**CAMCOG**	0.17 (0.63)	0.35 (0.91)	0.18 (0.8, 0.27)	<0.001	0.01
**AH4**	0.08 (0.61)	0.26 (0.66)	0.18 (0.09, 0.27)	<0.001	0.02
EXCLUDING THOSE WITH DEMENTIA AT PHASE 5					
**CAMCOG**	0.13 (0.55)	0.17 (0.49)	0.04 (-0.04, 0.11)	0.36	<0.001
**AH4**	0.07 (0.59)	0.22 (0.62)	0.16 (0.07, 0.24)	<0.001	0.01

*The proportion of variance in the dependent variable, that can be predicted by the independent variable

For peer review only - http://bmjopen.bmj.com/site/about/guidelines.xhtml

Table 2: The diagnostic utility of simple subjective cognitive decline (sSCD), for objective cognitive performance

	OBJECTIVE COGNITIVE DECLINE,* N(%)	NO OBJECTIVE COGNITIVE DECLINE, N(%)	P-VALUE	SENSITIVITY (95% CONFIDENCE INTERVAL)	SPECIFICITY (95% CONFIDENCE INTERVAL)
**WHOLE SAMPLE**					
	**CAMCOG +VE**	**CAMCOG -VE**			
**SSCD +VE**	90 (9)	201 (21)	0.03	36 (30-42)	72 (68-75)
**SSCD -VE**	163 (17)	515 (53)			
	**AH4 +VE**	**AH4-VE**			
**SSCD +VE**	71 (8)	205 (22)	<0.001	44 (36-52)	73 (70-76)
**SSCD -VE**	91 (10)	558 (60)			
	**CAMCOG OR AH4 +VE**	**CAMCOG OR AH4 -VE**			
**SSCD +VE**	132 (14)	159 (16)	<0.001	38 (33-43)	74 (71-78)
**SSCD -VE**	216 (22)	462 (48)			
**EXCLUDING DEMENTIA**					
	**CAMCOG +VE**	**CAMCOG -VE**			
**SSCD +VE**	67 (7)	199 (21)	0.36	31 (25-38)	72 (69-75)
**SSCD -VE**	148 (16)	513 (55)			
	**AH4 +VE**	**AH4-VE**			
**SSCD +VE**	61 (7)	196 (22)	<0.001	42 (34-51)	74 (71-77)
**SSCD -VE**	83 (9)	552 (62)			
	**CAMCOG OR AH4 +VE**	**CAMCOG OR AH4 -VE**			
**SSCD +VE**	109 (12)	157 (17)	0.002	35 (30-41)	75 (71-78)
**SSCD -VE**	201 (22)	460 (50)			

* Continuous scoring converted to a binary variable, +ve/-ve indicates whether a participant had declined more/less than 1 standard deviation from the mean rate, calculated amongst men without CIND nor dementia, between Phases 3 to 5.

sSCD = simple Subjective Cognitive decline; CAMCOG = Cambridge Cognitive Examination; AH4 = Alice Heim 4 test.

For peer review only - http://bmjopen.bmj.com/site/about/guidelines.xhtml

For peer review only

For peer review only - http://bmjopen.bmj.com/site/about/guidelines.xhtml

Table 3: Simple subjective cognitive decline and cognitive internal inconsistency, amongst people without objective cognitive decline (at phase 5).

MEN WITH NO OBJECTIVE COGNITIVE DECLINE, PHASE 5	SUBJECTIVE REPORT OF COGNITION		
**RIVERMEAD STORY RECALL TASK**	No sSCD, mean (SD)	sSCD, mean (SD)	P-value
**IMMEDIATE RECALL "IR"**	6.15 (2.82)	5.55 (2.59)	0.02
**DELAYED RECALL "DR"**	5.29 (2.78)	4.73 (2.59)	0.03
**IR-DR**	0.84 (1.45)	0.83 (1.41)	0.92
**RETENTION: DR/IR**	0.86 (0.37)	0.86 (0.35)	0.58
**RELATIVE DIFFERENCE: (IR-DR)/IR**	0.12 (0.37)	0.14 (0.35)	0.58
**CONSISTENCY OF RECALL ABILITY**	n (%)		Chi-squared P-value
**"CONSISTENT" (DR =/< IR)**	370 (80.8)	130 (82.3)	0.68
**"INCONSISTENT" (DR > IR)**	88 (19.2)	28 (17.7)	

sSCD = simple Subjective Cognitive decline

For peer review only - http://bmjopen.bmj.com/site/about/guidelines.xhtml

Table 4: Possible risk factors (phases 2 to 4) for simple Subjective Cognitive Decline (sSCD) (phase 5)

	n FOR UNADJUSTED OR	UNADJUSTED OR (95% CI)	ADJUSTED OR (95% CI) (n=601)	ADJUSTED OR (95% CI) EXCLUDING DEMENTIA (N=580)
AGE (YEARS)	1065	1.07 (1.03, 1.10)	1.12 (1.06, 1.17)	1.11 (1.05, 1.17)
SOCIAL CLASS (MANUAL/NON-MANUAL)	1020	1.10 (0.84, 1.45)	0.99 (0.65, 1.51)	0.95 (0.62, 1.46)
PREMORBID IQ (X 0.1)	956	0.90 (0.78, 1.03)	1.06 (0.84, 1.33)	1.05 (0.83, 1.32)
HIGH ALCOHOL USE (BINARY)	1022	0.96 (0.66, 1.39)	1.07 (0.63, 1.83)	1.20 (0.70, 2.07)
TEETOTAL (BINARY)	1022	1.37 (0.89, 2.11)	0.64 (0.31, 1.32)	0.60 (0.29, 1.27)
PROBABLE ISCHAEMIC HEART DISEASE (BINARY)	1015	1.62 (1.18, 2.22)	0.98 (0.59, 1.62)	1.03 (0.61, 1.72)
NUMBER OF VASCULAR MEDICATIONS	1065	1.33 (1.15, 1.55)	1.18 (0.92, 1.50)	1.20 (0.93, 1.54)
SMOKING (BINARY)	1022	1.10 (0.83, 1.44)	0.89 (0.60, 1.34)	0.85 (0.56, 1.30)
WAIST-HIP RATIO (X10)	990	1.19 (0.94, 1.50)	0.93 (0.66, 1.31)	0.89 (0.63, 1.27)
POOR SLEEP	983	1.91 (1.64, 2.22)	1.52 (1.19, 1.94)	1.55 (1.21, 1.99)
SLEEP-DISORDERED BREATHING	727	1.39 (1.17, 1.65)	1.13 (0.91, 1.40)	1.12 (0.90, 1.40)
MOOD SYMPTOMS (GHQ SCORE: CHRONIC)*	886	1.78 (1.53, 2.06)	1.27 (1.01, 1.59)	1.23 (0.97, 1.55)
TRAIT ANXIETY (STAI)*	1019	1.66 (1.44, 1.91)	1.38 (1.09, 1.74)	1.39 (1.09, 1.76)
RATE OF DECLINE CAMCOG	969	1.37 (1.13, 1.66)	1.18 (0.83, 1.69)	1.18 (0.72, 1.95)
RATE OF DECLINE AH4	925	1.59 (1.26, 1.99)	1.33 (0.95, 1.85)	1.36 (0.97, 1.92)

*standardised

For peer review only - http://bmjopen.bmj.com/site/about/guidelines.xhtml

Table 5: sSCD does not predict future cognitive outcomes (new CIND or dementia)

PREDICTOR AT PHASE 5: SSCD			
**OUTCOME AT PHASE 7**	CIND or dementia	Dementia	Decline in CAMCOG between phases 5 and 7*
**PARTICIPANTS**	Excluding those with CIND or dementia at phase 5	Excluding those with dementia at phase 5	Excluding those with dementia at phase 5
**MODEL**	Odds ratio (95% CI)	Odds ratio (95% CI)	Linear coefficient (95% CI)
**COMPLETE CASE ANALYSIS**	0.92 (0.49, 1.73)	1.35 (0.61, 2.99)	0.01 (-0.13, 0.15)
**MULTIPLE IMPUTATION**	0.98 (0.56, 1.74)	1.18 (0.53, 2.61)	-0.02 (-0.19, 0.15)
***WITH INTERACTION SSCD X PHASE 5 CAMCOG* MODEL**			Interaction term** linear coefficient (95% CI)
**COMPLETE CASE ANALYSIS**	-	-	0.03 (0.01, 0.05)
**MULTIPLE IMPUTATION**	-	-	0.02 (-0.00, 0.03)

All models controlled for: age, CAMCOG score at phase 5.

*The CAMCOG analysis also controlled for a dummy variable indicating CAMCOG points lost for hearing, vision, or manual dexterity at phase 7.

** The complete case analysis suggested there was an interaction effect between CAMCOG score and sSCD (both measured at phase 5), in predicting decline in CAMCOG from phase 5 to 7. sSCD predicted worse outcomes for those with a low CAMCOG score at phase 5; but SCD predicted better outcomes for those with a medium or high CAMCOG at phase 5. However, this interaction was not significant on the MICE analysis (p=0.08), therefore was dropped from the overall analysis.

For peer review only - http://bmjopen.bmj.com/site/about/guidelines.xhtml

References:

1. McWhirter L, Ritchie C, Stone J, Carson A. Functional cognitive disorders: a systematic review. The Lancet Psychiatry. 2020;7(2):191-207.
2. Burmester B, Leathem J, Merrick P. Subjective Cognitive Complaints and Objective Cognitive Function in Aging: A Systematic Review and Meta-Analysis of Recent Cross-Sectional Findings. Neuropsychology Review. 2016;26(4):376-93.
3. Crumley JJ, Stetler CA, Horhota M. Examining the relationship between subjective and objective memory performance in older adults: a meta-analysis. Psychol Aging. 2014;29(2):250-63.
4. Mol ME, van Boxtel MP, Willems D, Jolles J. Do subjective memory complaints predict cognitive dysfunction over time? A six-year follow-up of the Maastricht Aging Study. Int J Geriatr Psychiatry. 2006;21(5):432-41.
5. Jessen F, Amariglio RE, van Boxtel M, Breteler M, Ceccaldi M, Chételat G, et al. A conceptual framework for research on subjective cognitive decline in preclinical Alzheimer's disease. Alzheimer's & Dementia. 2014;10(6):844-52.
6. Slot RER, Sikkes SAM, Berkhof J, Brodaty H, Buckley R, Cavedo E, et al. Subjective cognitive decline and rates of incident Alzheimer's disease and non–Alzheimer's disease dementia. Alzheimer's & Dementia. 2019;15(3):465-76.
7. Pavisic IM, Lu K, Keuss SE, James S-N, Lane CA, Parker TD, et al. Subjective cognitive complaints at age 70: associations with amyloid and mental health. Journal of Neurology, Neurosurgery & Psychiatry. 2021;92(11):1215.
8. Brailean A, Steptoe A, Batty GD, Zaninotto P, Llewellyn DJ. Are subjective memory complaints indicative of objective cognitive decline or depressive symptoms? Findings from the English Longitudinal Study of Ageing. J Psychiatr Res. 2019;110:143-51.
9. Jessen F, Amariglio RE, Buckley RF, van der Flier WM, Han Y, Molinuevo JL, et al. The characterisation of subjective cognitive decline. The Lancet Neurology. 2020.
10. Visser PJ, Verhey F, Knol DL, Scheltens P, Wahlund L-O, Freund-Levi Y, et al. Prevalence and prognostic value of CSF markers of Alzheimer's disease pathology in patients with subjective cognitive impairment or mild cognitive impairment in the DESCRIPA study: a prospective cohort study. The Lancet Neurology. 2009;8(7):619-27.
11. Eckerström M, Göthlin M, Rolstad S, Hessen E, Eckerström C, Nordlund A, et al. Longitudinal evaluation of criteria for subjective cognitive decline and preclinical Alzheimer's disease in a memory clinic sample. Alzheimer's & Dementia: Diagnosis, Assessment & Disease Monitoring. 2017;8:96-107.
12. Ball HA, McWhirter L, Ballard C, Bhome R, Blackburn DJ, Edwards MJ, et al. Functional cognitive disorder: dementia's blind spot. Brain. 2020.
13. Ganguli M, Jia Y, Hughes TF, Snitz BE, Chang C-CH, Berman SB, et al. Mild Cognitive Impairment that Does Not Progress to Dementia: A Population-Based Study. J Am Geriatr Soc. 2019;67(2):232-8.
14. Banks J, Blundell R, Calderwood L. Wave 1- Health, Wealth and lifestyles of the older population in England: ELSA 2002. Marmot M, Banks J, Blundell R, Lessor C, Nazroo J, editors2002.
15. Mol ME, van Boxtel MP, Willems D, Verhey FR, Jolles J. Subjective forgetfulness is associated with lower quality of life in middle-aged and young-old individuals: a 9-year follow-up in older participants from the Maastricht Aging Study. Aging Ment Health. 2009;13(5):699-705.
16. Miller MA, Wright H, Ji C, Cappuccio FP. Cross-sectional study of sleep quantity and quality and amnestic and non-amnestic cognitive function in an ageing population: the English Longitudinal Study of Ageing (ELSA). PLoS One. 2014;9(6):e100991.
17. Roth M, Tym E, Mountjoy CQ, Huppert FA, Hendrie H, Verma S, et al. CAMDEX. A standardised instrument for the diagnosis of mental disorder in the elderly with special reference to the early detection of dementia. Br J Psychiatry. 1986;149:698-709.

For peer review only - http://bmjopen.bmj.com/site/about/guidelines.xhtml

18. Heim AW. Manual for the Group test of General Intelligence AH4. London, England: National Foundation for Educational Research.; 1955.
19. Wilson BA, Cockburn J, Baddeley AD. The Rivermead behavioural memory test. London: Thames Valley Test Company; 1985.
20. Jorm AF, Jacomb PA. The Informant Questionnaire on Cognitive Decline in the Elderly (IQCODE): socio-demographic correlates, reliability, validity and some norms. Psychol Med. 1989;19(4):1015-22.
21. von Elm E, Altman DG, Egger M, Pocock SJ, Gøtzsche PC, Vandenbroucke JP. Strengthening the Reporting of Observational Studies in Epidemiology (STROBE) statement: guidelines for reporting observational studies. Bmj. 2007;335(7624):806-8.
22. Tombaugh TN. The Test of Memory Malingering (TOMM): Normative data from cognitively intact and cognitively impaired individuals. Psychological Assessment. 1997;9(3):260.
23. Nelson HE. National adult reading test (NART) : for the assessment of premorbid intelligence in patients with dementia : test manual. Windsor, Berks: NFER-Nelson; 1982.
24. Rehm J, Hasan OSM, Black SE, Shield KD, Schwarzinger M. Alcohol use and dementia: a systematic scoping review. Alzheimers Res Ther. 2019;11(1):1-.
25. Liew TM, Yap P, Ng TP, Mahendran R, Kua EH, Feng L. Symptom clusters of subjective cognitive decline amongst cognitively normal older persons and their utilities in predicting objective cognitive performance: structural equation modelling. Eur J Neurol. 2019;26(9):1153-60.
26. Cosentino S, Devanand D, Gurland B. A Link between Subjective Perceptions of Memory and Physical Function: Implications for Subjective Cognitive Decline. Journal of Alzheimer's disease : JAD. 2018;61(4):1387-98.
27. Suo C, Gates N, Singh MF, Saigal N, Wilson GC, Meiklejohn J, et al. Midlife managerial experience is linked to late life hippocampal morphology and function. Brain imaging and behavior. 2017;11(2):333-45.
28. Goldstein FC, Loring DW, Thomas T, Saleh S, Hajjar I. Recognition Memory Performance as a Cognitive Marker of Prodromal Alzheimer's Disease. Journal of Alzheimer's disease : JAD. 2019;72(2):507-14.
29. Russo MJ, Campos J, Vazquez S, Sevlever G, Allegri RF. Adding Recognition Discriminability Index to the Delayed Recall Is Useful to Predict Conversion from Mild Cognitive Impairment to Alzheimer's Disease in the Alzheimer's Disease Neuroimaging Initiative. Front Aging Neurosci. 2017;9:46.
30. Oda H, Yamamoto Y, Maeda K. Neuropsychological profile of dementia with Lewy bodies. Psychogeriatrics. 2009;9(2):85-90.
31. Teodoro T, Edwards MJ, Isaacs JD. A unifying theory for cognitive abnormalities in functional neurological disorders, fibromyalgia and chronic fatigue syndrome: systematic review. Journal of Neurology Neurosurgery and Psychiatry. 2018;89(12):1308-19.
32. de Winter JCF, Dodou D, Hancock PA. On the paradoxical decrease of self-reported cognitive failures with age. Ergonomics. 2015;58(9):1471-86.
33. Bhome R, McWilliams A, Huntley JD, Fleming SM, Howard RJ. Metacognition in functional cognitive disorder- a potential mechanism and treatment target. Cogn Neuropsychiatry. 2019;24(5):311-21.
34. Chin J, Oh KJ, Seo SW, Na DL. Are depressive symptomatology and self-focused attention associated with subjective memory impairment in older adults? Int Psychogeriatr. 2014;26(4):573-80.
35. Schweizer S, Kievit RA, Emery T, Cam CAN, Henson RN. Symptoms of depression in a large healthy population cohort are related to subjective memory complaints and memory performance in negative contexts. Psychological medicine. 2018;48(1):104-14.
36. Pennington C, Ball H, Swirski M. Functional Cognitive Disorder: Diagnostic Challenges and Future Directions. Diagnostics (Basel). 2019;9(4).

For peer review only - http://bmjopen.bmj.com/site/about/guidelines.xhtml

Supplemental material for "Predictors and prognosis of population-based subjective cognitive decline: Longitudinal evidence from the Caerphilly Prospective Study (CaPS)"

Contents

Items derived from The Wisconsin Sleep Scale 1

Supplementary Figure 1: Caerphilly Prospective Study (CaPS) flowchart (for references, see main manuscript)........ 2

Supplementary Figure 2: Histogram displaying delayed recall minus immediate recall ("internal inconsistency") at phase 5, according to presence or absence of simple Subjective Cognitive Decline (sSCD). 3

Supplementary Table 1: Relative frequency of possible risk variables, amongst people with and without simple Subjective Cognitive Decline (sSCD) (Phase 5)........ 4

Supplementary Table 2: Missingness by variable, as a percentage of n=1225 participants at phase 5 5

Items derived from The Wisconsin Sleep Scale

The Wisconsin sleep scale is mainly composed of items scored on a 1-5 point scale. These items were separated into two groups, then summed and standardized: i) *Experiencing poor sleep*, including: difficulty initiating sleep; waking overnight; difficulty getting back to sleep; not feeling rested during the day; daytime sleepiness; dozing easily in the daytime; use of caffeine or other stimulants to stay awake; and napping during the day. This comprised 8 items, (alpha 0.77). Minimum and maximum scores prior to standardisation were 8 and 39. ii) Breathing-disordered Sleep, including: frequency of snoring; loudness of snoring; awareness of snoring; making gasp/ choke/ snort noises during sleep; waking with these noises; and frequency of momentary periods in sleep where you breathe abnormally. This comprised 6 items (alpha 0.77). Minimum and maximum scores prior to standardisation were 6 and 30. Note this approach excluded items on the Wisconsin questionnaire that were either scored on a substantially different scale, or assessing other aspects of sleep: how awake you feel today; restless legs/ twitches/ kicking during sleep; nasal congestion interrupting sleep; sleep latency; reported duration of sleep (weekdays and weekends); use of sleeping tablets; and allergies causing nasal congestion.

For peer review only

Supplemental Figure 1: Caerphilly Prospective Study (CaPS) flowchart

References: (1, 2, 3)

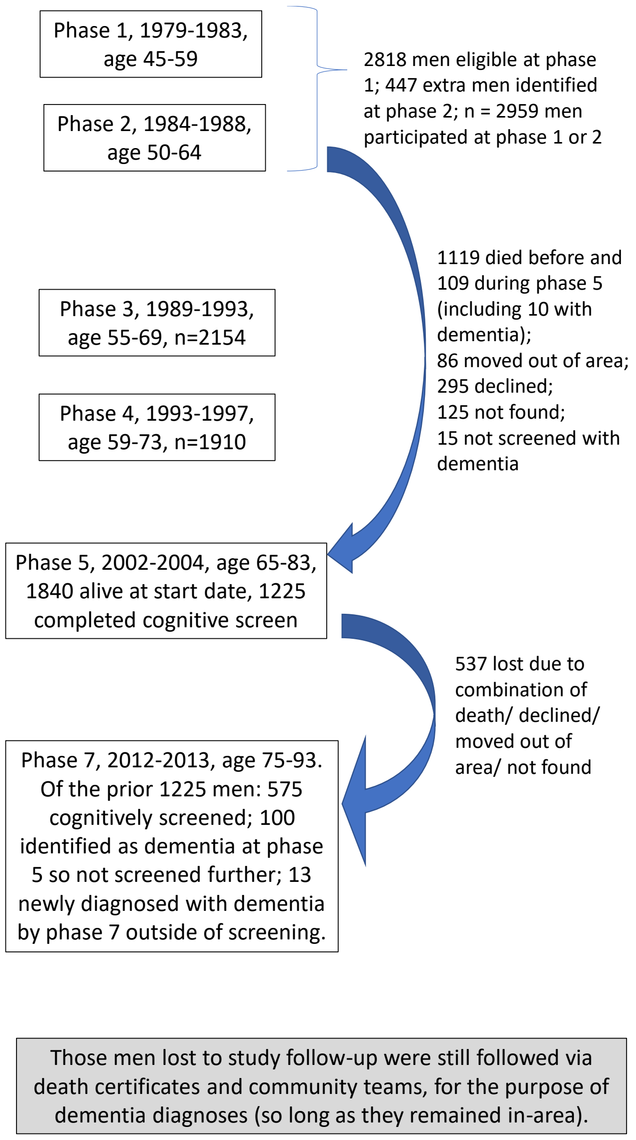

References:

1. Fish M, Bayer AJ, Gallacher JE, Bell T, Pickering J, Pedro S, et al. Prevalence and pattern of cognitive impairment in a community cohort of men in South Wales: methodology and findings from the Caerphilly Prospective Study. Neuroepidemiology. 2008;30(1):25-33.

2. Creavin ST, Gallacher J, Bayer A, Fish M, Ebrahim S, Ben-Shlomo Y. Metabolic syndrome, diabetes, poor cognition, and dementia in the Caerphilly prospective study. J Alzheimers Dis. 2012;28(4):931-9.

3. Elwood PC, Pickering J, Bayer A, Gallacher JEJ. Vascular disease and cognitive function in older men in the Caerphilly cohort. Age and Ageing. 2002;31(1):43-8.

Supplemental Figure 2: Histogram displaying delayed recall minus immediate recall ("internal inconsistency") at phase 5, according to presence or absence of simple Subjective Cognitive Decline (sSCD)

This analysis included only those men with no objective decline in measured cognition since Phase 3.

Score greater than 0 is "inconsistent". Note how roughly one fifth of men had this marker of internal inconsistency (regardless of subjective decline status).

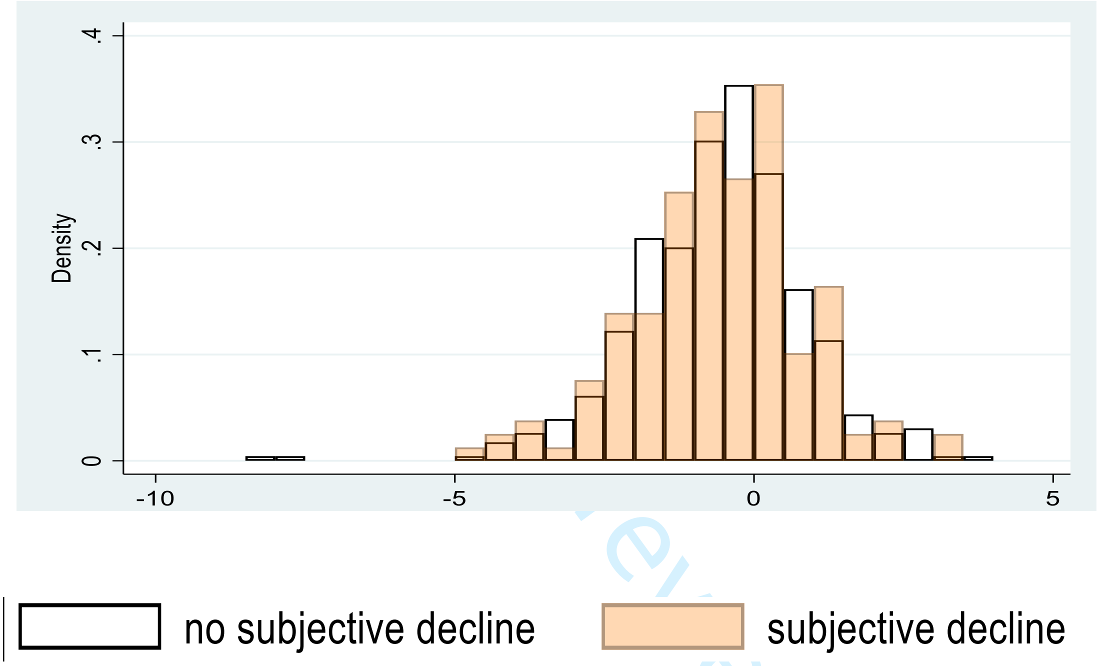

Supplemental Table 1: Relative frequency of possible risk variables, amongst people with and without simple Subjective Cognitive Decline (sSCD) (Phase 5)

	TOTAL N	NO SUBJECTIVE COGNITIVE DECLINE, n (%)	SUBJECTIVE COGNITIVE DECLINE, N (%)
**TOTAL: N**	1225	739 (69.4)	326 (30.6)
**CIND (PHASE 5)**	192	79 (10.7)	71 (21.8)
**DEMENTIA (PHASE 5)**	75	19 (2.6)	31 (9.5)
**MANUAL SOCIAL CLASS**	720	417 (59.0)	192 (61.3)
**LEFT SCHOOL BEFORE 14**	507	286 (38.7)	149 (45.7)
**TEETOTAL (AT ANY PHASE 3-5)**	120	63 (8.5)	37 (11.4)
**CUMULATIVE HIGH ALCOHOL INTAKE**	182	110 (14.9)	47 (14.4)
**CURRENT SMOKER (ANY OF PHASE 3-5)**	452	261 (36.8)	122 (39.0)
**PROBABLE ISCHAEMIC HEART DISEASE**	253	131 (17.7)	84 (25.8)
**ANTIDEPRESSANT MEDICATION (BINARY)**	15	6 (0.8)	8 (2.5)
	**ALL**	**NO SUBJECTIVE COGNITIVE DECLINE, MEAN (SD)**	**SUBJECTIVE COGNITIVE DECLINE, MEAN (SD)**
**AGE (YEARS)**	73.0 (4.2)	72.5 (4.0)	73.6 (4.2)
**PREMORBID IQ**	107.1 (9.9)	108.0 (9.9)	107.0 (9.7)
**BODY MASS INDEX**	26.9 (3.5)	27.0 (3.5)	26.8 (3.3)
**WAIST-HIP RATIO**	0.93 (0.06)	0.93 (0.06)	0.93 (0.06)
**NUMBER OF MEDICATIONS FOR VASCULAR RISK**	0.40 (0.83)	0.32 (0.71)	0.54 (1.05)
**EXPERIENCING POOR SLEEP**	-0.05 (0.95)	-0.23 (0.87)	0.33 (0.97)
**BREATHING-DISORDERED SLEEP**	0.02 (0.93)	-0.05 (0.88)	0.22 (0.98)
**GHQ (MOOD) CHRONIC SCORING**	9.0 (6.3)	7.8 (5.7)	11.3 (6.7)
**STAI (TRAIT ANXIETY)**	35.9 (8.9)	34.3 (8.5)	38.8 (9.3)
**CAMCOG RATE OF CHANGE PER YEAR**	-0.29 (0.96)	-0.17 (0.63)	-0.35 (0.91)
**AH4 RATE OF CHANGE PER YEAR**	-0.15 (0.64)	-0.08 (0.61)	-0.26 (0.66)

CIND = cognitive impairment, not depression; QGH = general health questionnaire (a measure of mood symptoms); STAI = Spielberger State-Trait Anxiety Inventory; CAMCOG = Cambridge Cognitive Examination; AH4= Alice Heim 4 test. *For missingness data, see Supplementary Table 1.*

For peer review only

Supplemental Table 2: Missingness by variable, as a percentage of n=1225 participants at phase 5

VARIABLE	PHASE OF DATA COLLECTION	% MISSING
Age	Baseline	(none)
Sex	Baseline	(none, all men)
Manual social class	Baseline	4.9
Left school at 14	Baseline	10.3
STAI	2	5.3
Probable ischaemic heart disease	3	5.6
Antidepressant medication (binary)	3	Missingness data not available
Premorbid IQ	3	12.1
Body mass index	3	10.5
Waist hip ratio	3	8.2
Experiencing poor sleep	3	9.1
Sleep-disordered breathing	3	34.0
Mood symptoms (GHQ)	3	17.8
CAMCOG	3	11.6
AH4	3	12.2
CAMCOG	4	15.4
AHR	4	16.2
Teetotal	3-5	4.8
Cumulative high alcohol intake	3-5	4.8
Smoker	3-5	4.7
Number of medications for vascular risk	3	Missingness data not available
Subjective Cognitive Decline	5	13.1
CAMCOG	5	0.4
AH4	5	6.9
Immediate recall	5	2.5
Delayed recall	5	2.5
CIND or dementia	5	0.2
CAMCOG (excluding men who had dementia by phase 5)	7	56.0
CIND or dementia (excluding men who had dementia by phase 5)	7	53.0

For peer review only

Reporting checklist for cohort study.

Based on the STROBE cohort guidelines.

Instructions to authors

Complete this checklist by entering the page numbers from your manuscript where readers will find each of the items listed below.

Your article may not currently address all the items on the checklist. Please modify your text to include the missing information. If you are certain that an item does not apply, please write "n/a" and provide a short explanation.

Upload your completed checklist as an extra file when you submit to a journal.

In your methods section, say that you used the STROBE cohortreporting guidelines, and cite them as:

von Elm E, Altman DG, Egger M, Pocock SJ, Gotzsche PC, Vandenbroucke JP. The Strengthening the Reporting of Observational Studies in Epidemiology (STROBE) Statement: guidelines for reporting observational studies.

For peer review only

		Reporting Item	Page Number
**Title and abstract**			
Title	#1a	Indicate the study's design with a commonly used term in the title or the abstract	1

Abstract	#1b	Provide in the abstract an informative and balanced summary of what was done and what was found	2
**Introduction**			
Background / rationale	#2	Explain the scientific background and rationale for the investigation being reported	4
Objectives	#3	State specific objectives, including any prespecified hypotheses	6
**Methods**			
Study design	#4	Present key elements of study design early in the paper	6
Setting	#5	Describe the setting, locations, and relevant dates, including periods of recruitment, exposure, follow-up, and data collection	6
Eligibility criteria	#6a	Give the eligibility criteria, and the sources and methods of selection of participants. Describe methods of follow-up.	6
Eligibility criteria	#6b	For matched studies, give matching criteria and number of exposed and unexposed	N/A
Variables	#7	Clearly define all outcomes, exposures, predictors, potential confounders, and effect modifiers. Give diagnostic criteria, if applicable	7-8
Data sources / measurement	#8	For each variable of interest give sources of data and details of methods of assessment (measurement). Describe	7-8

For peer review only

		comparability of assessment methods if there is more than one group. Give information separately for for exposed and unexposed groups if applicable.	
Bias	#9	Describe any efforts to address potential sources of bias	8-9
Study size	#10	Explain how the study size was arrived at	6
Quantitative variables	#11	Explain how quantitative variables were handled in the analyses. If applicable, describe which groupings were chosen, and why	8-9
Statistical methods	#12a	Describe all statistical methods, including those used to control for confounding	
8-9			
Statistical methods	#12b	Describe any methods used to examine subgroups and interactions	23
Statistical methods	#12c	Explain how missing data were addressed	8
Statistical methods	#12d	If applicable, explain how loss to follow-up was addressed	8
Statistical methods	#12e	Describe any sensitivity analyses	
9			

Results

For peer review only

Participants	#13a	Report numbers of individuals at each stage of study—eg numbers potentially eligible, examined for eligibility, confirmed eligible, included in the study, completing follow-up, and analysed. Give information separately for for exposed and unexposed groups if applicable.	Figure 1
Participants	#13b	Give reasons for non-participation at each stage	Figure 1
Participants	#13c	Consider use of a flow diagram	
Figure 1			
Descriptive data	#14a	Give characteristics of study participants (eg demographic, clinical, social) and information on exposures and potential confounders. Give information separately for exposed and unexposed groups if applicable.	Table 1
Descriptive data	#14b	Indicate number of participants with missing data for each variable of interest - Supplementary Table 1	
Descriptive data	#14c	Summarise follow-up time (eg, average and total amount)	
Figure 1			
Outcome data	#15	Report numbers of outcome events or summary measures over time. Give information separately for exposed and unexposed groups if applicable.	
Page 11			

For peer review only

Main results	#16a	Give unadjusted estimates and, if applicable, confounder-adjusted estimates and their precision (eg, 95% confidence interval). Make clear which confounders were adjusted for and why they were included	Tables
Main results	#16b	Report category boundaries when continuous variables were categorized	7
Main results	#16c	If relevant, consider translating estimates of relative risk into absolute risk for a meaningful time period	
N/A			
Other analyses	#17	Report other analyses done—eg analyses of subgroups and interactions, and sensitivity analyses	Tables
**Discussion**			
Key results	#18	Summarise key results with reference to study objectives	12
Limitations	#19	Discuss limitations of the study, taking into account sources of potential bias or imprecision. Discuss both direction and magnitude of any potential bias.	14-15
Interpretation	#20	Give a cautious overall interpretation considering objectives, limitations, multiplicity of analyses, results from similar studies, and other relevant evidence.	12-16
Generalisability	#21	Discuss the generalisability (external validity) of the study results	15

For peer review only

Other Information

Funding	#22	Give the source of funding and the role of the funders for the present study and, if applicable, for the original study on which the present article is based	17

None The STROBE checklist is distributed under the terms of the Creative Commons Attribution License CC-BY. This checklist can be completed online using https://www.goodreports.org/, a tool made by the EQUATOR Network in collaboration with Penelope.ai

For peer review only

BMJ Open

Predictors and prognosis of population-based subjective cognitive decline: Longitudinal evidence from the Caerphilly Prospective Study (CaPS)

Journal:	*BMJ Open*
Manuscript ID	bmjopen-2023-073205.R1
Article Type:	Original research
Date Submitted by the Author:	01-Aug-2023
Complete List of Authors:	Ball, Harriet; University of Bristol, Population Health Sciences<br>Coulthard , Liz ; University of Bristol Medical School, Translational Health Sciences<br>Fish, Mark; Royal Devon University Healthcare NHS Foundation Trust<br>Bayer, Antony; Cardiff University, Institute of Primary Care and Public Health<br>Gallacher, John; Oxford<br>Ben-Shlomo, Yoav; University of Bristol, Population Health Sciences
**Primary Subject Heading**:	Epidemiology
Secondary Subject Heading:	Mental health, Neurology
Keywords:	Dementia < NEUROLOGY, Dementia, Delirium & cognitive disorders < PSYCHIATRY, EPIDEMIOLOGY

SCHOLARONE™
Manuscripts

I, the Submitting Author has the right to grant and does grant on behalf of all authors of the Work (as defined in the below author licence), an exclusive licence and/or a non-exclusive licence for contributions from authors who are: i) UK Crown employees; ii) where BMJ has agreed a CC-BY licence shall apply, and/or iii) in accordance with the terms applicable for US Federal Government officers or employees acting as part of their official duties; on a worldwide, perpetual, irrevocable, royalty-free basis to BMJ Publishing Group Ltd ("BMJ") its licensees and where the relevant Journal is co-owned by BMJ to the co-owners of the Journal, to publish the Work in this journal and any other BMJ products and to exploit all rights, as set out in our licence.

The Submitting Author accepts and understands that any supply made under these terms is made by BMJ to the Submitting Author unless you are acting as an employee on behalf of your employer or a postgraduate student of an affiliated institution which is paying any applicable article publishing charge ("APC") for Open Access articles. Where the Submitting Author wishes to make the Work available on an Open Access basis (and intends to pay the relevant APC), the terms of reuse of such Open Access shall be governed by a Creative Commons licence – details of these licences and which Creative Commons licence will apply to this Work are set out in our licence referred to above.

Other than as permitted in any relevant BMJ Author's Self Archiving Policies, I confirm this Work has not been accepted for publication elsewhere, is not being considered for publication elsewhere and does not duplicate material already published. I confirm all authors consent to publication of this Work and authorise the granting of this licence.

For peer review only

Predictors and prognosis of population-based subjective cognitive decline: Longitudinal evidence from the Caerphilly Prospective Study (CaPS)

Harriet Ball[1], Elizabeth Coulthard[2], Mark Fish[3], Antony Bayer[4], John Gallacher[5], Yoav Ben-Shlomo[1].

1. Population Health Sciences, Bristol Medical School, University of Bristol, UK
2. Translational Health Sciences, Bristol Medical School, University of Bristol, UK
3. Royal Devon University Healthcare Trust, Exeter, UK
4. Division of Population Medicine, Cardiff Medical School, Cardiff University, UK
5. Department of Psychiatry, University of Oxford, UK

Corresponding author: Harriet Ball, harriet.ball@bristol.ac.uk Southmead Hospital Learning and Research Building, Bristol Medical School, University of Bristol.

Word count: 4,878

For peer review only

For peer review only - http://bmjopen.bmj.com/site/about/guidelines.xhtml

Article summary

Objectives: to understand associations between the subjective experience of cognitive decline, and objective cognition. This subjective experience is often conceptualised as an early step towards neurodegeneration, but this has not been scrutinised at the population level. An alternative explanation is poor meta-cognition, the extreme of which is seen in Functional Cognitive Disorder (FCD).

Design: prospective cohort (Caerphilly Prospective Study)

Setting: population-based, South Wales, UK

Participants: This men-only study began in 1979; 1225 men participated at average age 73 in 2002-2004, including assessment of simple Subjective Cognitive Decline (sSCD, defined as subjective report of worsening memory or concentration). Dementia outcomes were followed up to 2012-2014. Data on non-completers was additionally obtained from death certificates and local health records.

Primary and Secondary Outcome Measures: The primary outcome measure was incident dementia over 10 years. Secondary outcome measures included prospective change in objective cognition, and cross-sectional cognitive internal inconsistency (the existence of a cognitive ability at some times, and its absence at other times, with no intervening explanatory factors except for focus of attention).

Results: sSCD was common (30%) and only weakly associated with prior objective cognitive decline (sensitivity 36% [95% CI 30-42], specificity 72% [95% CI 68-75]). Independent predictors of sSCD were older age, poor sleep quality and higher trait anxiety. Those with sSCD did not have excess cognitive internal inconsistency, but results suggested a mild attentional deficit. sSCD did not predict future objective cognitive change (linear regression coefficient -0.01 (95% CI -0.13, 0.15)).

Conclusions: sSCD is weakly associated with prior objective cognitive decline, and does not predict future cognition. Prior sleep difficulties and anxiety were the most robust predictors of sSCD. sSCD in

For peer review only

For peer review only - http://bmjopen.bmj.com/site/about/guidelines.xhtml

the absence of objective decline appears to be a highly prevalent example of poor meta-cognition (i.e., poor self-awareness of cognitive performance), which could be a driver for later FCD.

Strengths and limitations of this study

- The design was prospective and population-based, with detailed collection of many potential risk or confounding factors, from middle to older age
- Outcome measures included objective test scores, and clinical dementia diagnoses (including local clinic and death certificate records for those who did not participate to the end)
- The study only included men from the outset, so we cannot extrapolate results to women

Funding: CaPS was funded by the Medical Research Council (grant G9824960-E01/1), and phase 5 was funded by a grant from the Alzheimer's Society.

Competing interests: None of the authors declare any competing interests.

Keywords: Dementia, subjective cognitive decline, functional cognitive disorder

For peer review only

For peer review only - http://bmjopen.bmj.com/site/about/guidelines.xhtml

BACKGROUND

We know that cognitive symptoms are common, and many people who attend memory clinics due to cognitive symptoms turn out not to have a progressive cognitive disorder.(1) The link between subjective reports and objective evidence of cognitive decline is surprisingly weak,(2-4) or sometimes found only in subgroups. An international working group (SCD-I) has published criteria for “pre-MCI Subjective Cognitive Decline” as those who experience persistent decline in cognitive ability relative to their baseline; despite scoring in normal ranges on objective cognitive tests; and excluding those meeting criteria for Mild Cognitive Impairment (MCI) or dementia, that explained by psychiatric or neurologic disease (except AD), medical disorder, medication or substance abuse.(5) This definition has been created to harmonise research exploring evidence of neurodegeneration. However, this definition excludes many in the population with new-onset subjective experience of cognitive decline (which is important since this experience, regardless of the exclusions in the definition, drives their concern and potential presentation to medical services). We define *simple* subjective cognitive decline (sSCD) as purely the subjective experience, irrespective of objective cognition or medical or psychiatric disorders.

A multi-cohort study found the risk of dementia in those with SCD (as defined by the SCD-I) was driven very largely by clinic-based rather than community or population-based samples.(6) Similarly, population-level association between SCD and amyloid status is masked once controlling for anxiety and demographic variables.(7) In the English Longitudinal Study of Ageing (ELSA) cohort,(8) although objective cognitive changes predicted subsequent worsening in subjective cognition, deterioration in subjective cognition did not prospectively predict objective cognition. Therefore, isolated subjective cognitive problems *often do not* herald the start of a progressive neurodegenerative process. Research attention is now focusing on a narrower group who have extra clinical features (“SCD-plus"), but who are yet further from the common population experience of subjective worsening in cognitive ability. “SCD-plus” refers to Subjective Cognitive Decline plus at least one additional

For peer review only - http://bmjopen.bmj.com/site/about/guidelines.xhtml

feature including: amnestic rather than non-amnestic complaints; onset <5 years ago; onset >age 60 years; concern associated with SCD; persistence of SCD over time; help-seeking; confirmation of decline by an informant).(9) “SCD plus” has been associated with a higher risk of developing objective cognitive deficits,(10) or frank dementia, than those with SCD alone.(11) But it is also likely that “SCD plus” would be over-represented in clinic populations relative to those in the general population.

The aim of this paper is to explore population-based associations with the subjective experience of cognitive decline (sSCD), since outcomes are likely to be different than when examining clinic-based populations that are less representative. The latter are likely to have worse outcomes, which might make more aggressive investigation and potential treatment appropriate, but may not hold for the general population with subjective cognitive problems. This is important to consider since the typical cases seen currently in clinic may change to include more people in the light of novel therapies and wider public awareness of neurodegenerative problems. This paper also takes a more nuanced and dimensional view of any association with objective cognitive change, noting individual differences in test scores rather than whether someone is just above versus just below a cutoff for “abnormal”.

A subjective experience of cognitive difficulty that outstrips objective evidence, when severe and impairing, is a key feature of Functional Cognitive Disorder(12); but a milder experience might be common in the wider population. Functional symptoms (which can wax and wane) therefore might explain *some* of the “recovery” of people with scores in the Mild Cognitive Impairment range to the normal range (up to one third in community based samples [(13)]). Another key features of FCD is cognitive “internal inconsistency”, which is when someone struggles to perform a cognitive ability, for which they are demonstrably capable, without any external factor intervening, aside from perhaps a change in focus of attention.(12, 14)

For peer review only - http://bmjopen.bmj.com/site/about/guidelines.xhtml

Population-based analyses show subjective cognitive concerns are more common in people who are older, of lower social class and less educated[15]; and are associated with worse quality of life, and higher anxiety[16]. Sleep disturbance is associated with objective cognitive dysfunction, more so at older ages[17], but less has been reported about its association with subjective cognition.

The current paper uses a prospective cohort of men in Wales, with in-depth cognitive assessments, and data spanning over three decades, to examine the prevalence of sSCD, its relation to objective cognitive change, and prospective associations with potential causative factors. Our research questions were:

1) Is sSCD is a good indicator of prior objective cognitive decline? This is relevant because clinicians might put more weight on a report of subjective cognitive decline, than an objective test score in a broadly normal range.
2) Do those with sSCD in the absence of objective decline, also have features of cognitive internal inconsistency?
3) Does sSCD have different risk factors compared to those for objective cognitive decline ?
4) Is sSCD associated with risk of future objective cognitive decline or dementia?

METHODS

The Caerphilly Prospective Study (CaPS) began in 1979 (Supplemental Figure 1) (18-20). Men aged 45-59 were identified from electoral rolls and general practice lists in Caerphilly and surrounding villages in South Wales, UK. Women were excluded as the initial focus was on cardiovascular health and it was felt that too few events would be accrued in the early follow-up. Beginning in phase 3, (age 55-69 years) cognitive assessments were added including the Cambridge Cognitive Examination (CAMCOG)[21], the Alice Heim 4 test (AH4)[22] and the Rivermead Behavioural Memory Test (RBMT,[23] which includes immediate recall of a 21-clause story, and delayed recall after 20 minutes). Cognitive assessments were also repeated at Phases 4 and 5. At Phase 5 (mean age 73 years), a subset of

For peer review only - http://bmjopen.bmj.com/site/about/guidelines.xhtml

participants (those who could not complete the CAMCOG, or scored 82 or fewer, or showed at least a 10 point decline over time) were invited to a clinical assessment to identify those who had dementia, and those who had "cognitive impairment not dementia" (CIND, which includes those with MCI). Informants (e.g., spouses or children) also rated change in participants' day to day abilities (IQCODE(24)).

A further assessment of *likely* dementia status was made holistically at Phase 7, by a cognitive disorders clinician (AB), using all available information, including longitudinal CAMCOG score, IQCODE, and all medical records. Those who had previously been classified as having dementia at Phase 5 were assumed to still have this diagnosis and were not re-evaluated. By this phase (mean age 82 years), some men had other health problems making it difficult to complete the cognitive assessments, leading to altered scores related to factors such as hearing, vision or manual dexterity. We have followed the STROBE cohort reporting guidelines.(25)

In assessing decline in objective cognitive test scores from Phases 3 to 5, we focused firstly on an instrument typically used as a dementia screen (CAMCOG), and secondly a scale more often used to indicate reasoning skills in the general population (AH4). The mean score at Phases 3 and 4 (or just one if the other was missing), was subtracted from the score at Phase 5, and divided by the time interval, to indicate rate of change over time (years). We defined "objective cognitive decline" as a decline of one standard deviation below the mean decline, of those men who had neither dementia nor CIND at Phase 5. Although an arbitrary cutoff, this value was chosen to capture a potentially subtle difference from an expected average decline over 8-12 years which is unlikely to be due to merely random error; with the benefit of premorbid detailed cognitive assessment, this difference might be smaller than what is felt to be a meaningful decline in a clinical setting.

At Phase 5, men were asked about their subjective cognition via two questions: In the last 12 months my *memory/concentration* (both asked independently) has: improved, not changed, got a

For peer review only - http://bmjopen.bmj.com/site/about/guidelines.xhtml

little worse, got a lot worse. These we recoded as 0=improved/no change, 1=little worse, 2=lot worse to produce (0-2) scores for memory and concentration. Scores on these two items were correlated (Spearman rho 0.64, p<0.001) and we combined them to generate a total score (0-4). We also created a binary variable (sSCD) defined as a score of 2 or more out of 4. Therefore our definition of SCD is more basic than SCD as defined earlier by the international working group.

"Cognitive internal inconsistency" was not a focus of this study at the time of data collection. Performance validity tests exist, but are protracted and were not performed as part of this study; further, the literature around these tests mainly focus on identification of conscious malingering, eg the TOMM.(26) We therefore looked for ways to identify unambiguous cognitive internal inconsistency in routinely collected data. We pragmatically operationalised this as delayed recall being paradoxically higher than immediate recall on the RBMT story recall task (i.e., looking at a memory component of internal inconsistency), and compared the frequency of this across those with and without sSCD. This is very far from a definitive test for FCD, but we would expect it to be present more often if a group included an excess of people with FCD. Due to the constraints of the data collected, it was not possible to identify a similarly unambiguous signal for cognitive components other than memory.

We considered the following variables as important confounders: age, social class (manual vs non-manual), years of education (before or after age 14 years) and premorbid IQ proxied by the National Adult Reading Test (NART)[27]. We assessed neurodegenerative risk markers at phases 2 to 4. These included probable ischaemic heart disease (assessed through hospital records and ECG changes), BMI and waist-hip ratio, smoking status, and medication history indicating vascular risk (i.e., medications whose indications were unequivocally for blood pressure, vascular disease, diabetes, or lipid-lowering). Alcohol exposure was indexed both as a binary marker of avoidance ("teetotal" at *any* of phases 2 through 4), and a binary indicator of the highest cumulative alcohol

For peer review only - http://bmjopen.bmj.com/site/about/guidelines.xhtml

use at Phases 2 through 4 (those drinking an average of 4 or more units per day across the period), due to previous work suggesting a non-linear association between alcohol and cognition[28]. At phase 3 we also assessed mood symptoms, using the chronic scoring of the General Health Questionnaire, the Spielberger State-Trait Anxiety Inventory, and use of any anti-depressant medication. Sleep problems were assessed using items from the Wisconsin Sleep Questionnaire, divided into "experiencing poor sleep" and "breathing-disordered sleep" (see Supplemental material).

Statistical analysis plan

The present study includes men who took part at phase 5, including the cognitive assessments (*n*=1,225). We calculated the prevalence of sSCD at phase 5 as the number of cases divided by the population at risk. We compared objective cognitive decline between phases 3 and 5, either as a continuous score (t-test) or binary measure "objective cognitive decline" (chi-squared test, using CAMCOG and AH4 separately) for those with and without sSCD at phase 5. We also derived the sensitivity and specificity of sSCD in relation to objective decline (Yes/No) as the gold standard. Amongst those with no objective cognitive decline by phase 5, we tested for an association between sSCD and cognitive internal inconsistency, using chi-squared test. We ran multivariable logistic regression models to identify possible causal contributors (measured at phases 2-4) to sSCD (at phase 5). We tested whether sSCD (at phase 5) predicted either a decline in CAMCOG (linear regression), or worsening cognitive severity category (logistic regression), between phases 5 and 7.

To address the potential bias when undertaking complete case analysis of potential causal contributors to sSCD, we included multiple imputation with chained equations (MICE) and pooled estimates using Rubin's rules across 50 imputed datasets. The imputation model used all the variables included in the analysis model (i.e., all the potential predictors of sSCD, as well as sSCD itself). To address potential survivor bias, we included a MICE model when looking at phase 7 outcomes, using the same principles and the same imputed variables as in the aforementioned

For peer review only - http://bmjopen.bmj.com/site/about/guidelines.xhtml

imputation, except we additionally included all variables that were in the phase 7 analysis model, in the imputation model.

As sensitivity analyses, we repeated the above excluding those with a pre-existing diagnosis of dementia. This is because in a clinical setting, it is usually relatively straightforward to identify people whose cognitive impairment is severe enough to be categorised as dementia; the diagnostic difficulty lies in understanding causes amongst those whose cognitive difficulty is milder. In addition, it excludes those who might have missing data on items about subjective cognition due to being severely cognitively impaired (which could introduce bias). We also repeated analyses looking at the memory-only versus concentration-only aspects of subjectively reported cognition.

Analyses were conducted in STATA v16.

Patient and public involvement

This study began in the 1970s when it was not normal practice to involve participants in the design and co-production of research. However, the researchers kept the participants well informed of the research progress and key findings through regular newsletters and including a 25th follow-up birthday party where all survivors met the research team and the Welsh Minister of Health. Over the study duration, ad hoc feedback has occurred with study participants writing or speaking to the research team with their views about the study. For example, a sensitive question was dropped based on participants’ comments.

RESULTS

Sociodemographics, and frequency of risk variables

1,225 men took part at Phase 5 including completion of cognitive assessments, with mean age of 73 (range 65-83) years (Supplemental Figure 1). 62% of men (n=720) were of manual social class. 46% (n=507) had left school by age 14 (in later analyses, we use NART-IQ as a more normally distributed

For peer review only - http://bmjopen.bmj.com/site/about/guidelines.xhtml

proxy for amount of education received). 75 (6.1%) were assessed as having dementia, and 192 (15.7%) as having CIND. 326 participants (30.6%) had sSCD. Unadjusted, subjects with sSCD were more likely to have CIND, Dementia, lower education, probable IHD, be older, report worse sleep, have a worse GHQ score, and show slightly greater declines in their CAMCOG and AH4 scores (Supplemental Table 1). sSCD status was missing for 160/1225 (13%) amongst the whole cohort, 42/192 (22%) amongst those with CIND, and 25/75 (33%) amongst those with dementia. Only 15 men (1.2%) were on antidepressant medication; due to low prevalence, this variable was not analysed further.

Of those who did not already have dementia at Phase 5, 37/573 (6.5%) had possible dementia by phase 7. Of those who did not already have CIND at phase 5, 84/528 (15.9%) had CIND or dementia by phase 7. Of those who were classified as not having dementia at phase 5, 575/1148 (50.1%) had missing data on this measure at phase 7.

Question 1: Is subjective cognitive decline (at phase 5) a good indicator of prior objective cognitive decline?

Objective CAMCOG cognitive change was scored continuously, and the average drop (from the baseline of Phase 3 or 4, to Phase 5) was 2.7 points per year in those developing dementia, 0.7 per year in those developing CIND, and 0.06 per year in those without either by Phase 5. The equivalent figures for AH4 were 0.9, 0.2 and 0.1 respectively. Objective changes in cognition were slightly worse in those with sSCD (Table 1), but the proportion of variance explained was very low. These associations were attenuated when excluding those with dementia, and this (unadjusted) association remained statistically significant for AH4 (but not for CAMCOG).

Of people who reported sSCD, the majority did not have objective cognitive decline as defined by >=1 standard deviation decline (Table 2). The utility for sSCD as a screening tool for objective decline was poor with a sensitivity of between 36-44% and specificity of 72-75%.

For peer review only - http://bmjopen.bmj.com/site/about/guidelines.xhtml
For peer review only

Question 2: Do those with subjective cognitive decline but no objective decline (at phase 5), have features of cognitive internal inconsistency?

In the Rivermead story recall test, subjects reporting sSCD did slightly worse than others on immediate recall (5.55 versus 6.15, p=0.02), and then proportionately worse on delayed recall (4.73 versus 5.29, p=0.03). The worse score on delayed recall was in proportion to the worse performance on immediate recall and more reflective of a mild attentional deficit than a true amnestic difficulty. There was no evidence of excess cognitive internal inconsistency (indicated by a higher score on delayed than immediate recall) when comparing those with and without sSCD (0.83 vs 0.84, p=0.92) (Table 3 and Supplemental Figure 2).

Question 3: Which characteristics predict subjective cognitive decline (at phase 5), and are these different to risk factors traditionally linked to objective cognitive decline

Variables that predicted sSCD in unadjusted logistic models included: age; probable ischaemic heart disease; vascular medications; experiencing poor sleep; breathing-disordered sleep; mood symptoms; trait anxiety; rate of decline in CAMCOG; and rate of decline in AH4 (Table 4, and Supplemental Table 3). There was no unadjusted association with social class, premorbid IQ, alcohol use (either high use or teetotal status), tobacco exposure, BMI, waist-hip ratio, nor baseline measures of CAMCOG and AH4. However, in a model simultaneously adjusting for all these factors, the only significant independent predictors remaining were: age, experiencing poor sleep, mood symptoms, and trait anxiety (respective ORs: 1.12, 1.52, 1.27 and 1.38). These associations (except mood symptoms, which became borderline non-significant) remained when excluding participants with a clinical diagnosis of dementia at phase 5.

Due to missing data, the adjusted complete case analysis only included 601 participants. This model, but with multiple imputation (n=1,225), found the same significant independent predictors, with the addition of one variable (rate of decline in AH4, OR 1.50, 1.16-1.95, see Supplemental Table 3).

For peer review only - http://bmjopen.bmj.com/site/about/guidelines.xhtml

Question 4: Does subjective cognitive decline (phase 5) predict future CIND and dementia (phase 7)?

sSCD at phase 5 did not predict worsening cognition at phase 7 (Table 5), when examined either by diagnosis (new CIND or new dementia) or by change in CAMCOG score. Due to attrition to phase 7, this was assessed both by complete case analysis and multiple imputation.

Sensitivity analyses

Results of analyses excluding those with a diagnosis of dementia are reported above.

We replaced overall sSCD with reported complaints in either memory-only or concentration-only. These showed the same patterns of association with measures of objective cognition, internal inconsistency (lack of association), possible confounders and risk factors, and future dementia (lack of association).

DISCUSSION

In this population-based sample of 1,225 older men in South Wales, we found only a weak association between objective cognitive change and subjective report of that same change, partially accounted for by some of the subjective group having dementia. Those who had subjective cognitive concerns without objective cognitive decline, nonetheless, showed a mild deficit in attentional processes. At the population level, sSCD has low sensitivity and specificity to identify objective evidence of cognitive decline (both missing many people who lack insight into their cognitive problems; and detecting people who are concerned in the absence of objective problems). Independent predictors for sSCD included preceding anxiety or mood symptoms, and poor self-reported sleep, as well as older age, but not other variables known to be risk factors for neurodegeneration. sSCD, adjusted for objective cognition, did not predict later CIND or dementia.

For peer review only - http://bmjopen.bmj.com/site/about/guidelines.xhtml

For peer review only

This is the first large population-based cohort study of subjective experience of cognitive decline which can look at both predictors as well as future clinical outcomes, with a wide range of potential confounders measured at younger ages, and hence is less prone to bias and reverse causation. We found no prospective association between subjective cognitive decline and later objective cognitive decline (either in numerical score or severity category i.e., CIND or dementia). Naturally, a larger sample size would have given greater power to detect a more modest effect. However, this negative finding suggests that subjective cognitive symptoms, by themselves, are not substantially (on a population level) the manifestation of a neurodegenerative condition. We found a weak association between prior objective cognitive decline and subjective report of decline in cognition. The interpretation of this association is complex since those with severely impaired cognition might lack insight or even not understand the question, but also there are a large number of people in the general population who feel their cognition is suboptimal. Previous studies that have found a more convincing association have largely been clinic-based. The reasons for this discrepancy likely include differences in populations according to who seeks and can access clinic assessment (which could include socioeconomic differences, age, comorbidities and ethnicity for example)(29). Most clinic-based studies of SCD often have participants with "SCD-plus" (i.e., extra factors, such as partner concern, that make neurodegeneration more likely and may increase their chances of reaching clinic). The choice of test and cut-off, and cross-sectional versus decline measure, can also affect results. Our decline measure is potentially a quite sensitive measure of change over time, given that we had a premorbid baseline measure, which is not present in clinic-based studies that begin when patients already present. The exact question framing also matters regarding self-reported cognitive difficulties, since some items are more likely to index normal ageing, and others pathological changes.[30-32]

For peer review only - http://bmjopen.bmj.com/site/about/guidelines.xhtml

Of our two objective cognitive tests, AH4 showed a slightly more robust association with sSCD than did CAMCOG (AH4 assesses complex verbal and numerical reasoning, analogous to an IQ test(22); CAMCOG measures faculties more commonly assessed in the dementia clinic(21)). Perhaps the population variance in complex reasoning more closely mirrors peoples' understanding of what it is to have memory or concentration problems. Alternatively, perhaps AH4 captures more meaningful variance in the normal range of cognition, whereas CAMCOG is better at detecting the more severe end (where those with cognitive difficulties often lack insight). Either way, it may be useful to incorporate measures similar to AH4 into neuropsychometry batteries for patients with suspected functional neurological symptoms, as this could represent a measure capable of tracking progress, providing a useful discussion point with patients.

Amongst men with subjective cognitive symptoms despite a lack of objective cognitive decline, we found they fared slightly worse (compared to those without cognitive symptoms) in a task of immediate recall, but no worse than expected on delayed recall of the initially remembered items. This reinforces the suggestion that SCD is linked to a slight deficit in attentional processes (despite not having dipped on overall cognitive test scores over many years). This pattern is distinct from the amnestic processes typically seen in early Alzheimer's disease(33, 34) or the attentional problems alongside wider progressive cognitive problems seen in dementia with Lewy bodies[(35)]. It is also of note that this group did not exhibit cognitive internal inconsistency (no evidence of scoring *better* on delayed recall than on immediate recall). This is not entirely surprising, as our group are quite distinct from clinic-based patients with Functional Cognitive Disorder in whom this characteristic is a key part of diagnosis(12) (and in whom more than a one-off example of evidence of internal inconsistency should be sought). Attentional problems have been identified in other conditions within the umbrella of functional somatic syndromes, when such patients have been tested cognitively (36). It is also important to recognise that our pragmatic measure of "cognitive internal

For peer review only - http://bmjopen.bmj.com/site/about/guidelines.xhtml

inconsistency" is identifiable in approximately one fifth of normal healthy people (Supplemental Figure 2), a surprisingly high number if only taking this one snapshot measurement into account.

We found that sSCD was more common at older ages. The same has been found in individual studies where a broad age range is considered[8]. When comparing across studies of people of differing ages, this association is less robust(1), probably in part due to heterogeneity between included studies. Reasons for this association could include differences in meta-cognitive processes by age; older people resigning to using work-arounds; age-related cultural expectations; or the way questions are framed leading to self-rating against different comparator groups; as well as some loss of insight when objective cognitive deficits take over[37]. The dip in subjective cognition with age may reflect the recognised small dip in objective cognitive abilities at older ages, as well as higher prevalence of neurodegeneration. However, factors typically associated with neurodegenerative change (vascular health, alcohol use) did not predict later sSCD in the fully adjusted model. This also held in an analysis excluding people with dementia. We interpret this to indicate that subjective cognitive decline is predicted by objective cognitive decline and its determinants, but also has important predictors independent of objective cognition. These predictors included prior reports of poor sleep quality, and prior anxiety symptoms. This supports the proposition that anxiety and poor sleep may be contributing to sSCD (rather than being a by-product of worry about cognitive change). These risk factors for sSCD may also predict functional cognitive symptoms. It is also notable that anxiety and poor sleep are relevant in our study which consisted of older males (whereas the literature and clinical diagnoses of functional disorders focus more on younger females). The association between subjective and objective cognition may be different in women, in particular since women may have an extra mid-life peak of subjective cognitive concern.(15)

Subjective reports of poor sleep often go alongside sSCD, however when using actigraphy(an objective assessment of sleep quality), the association is less or even reversed [1]. There are likely

For peer review only

qualitatively different sleep problems amongst those with sSCD, compared to those with organic changes to sleep in early dementia.

Clinical implications

Subjectively experiencing cognitive decline in the absence of an objective decline, is a marker of poor meta-cognition (i.e. poor ability to reflect on and monitor one's own cognitive abilities). This is a process postulated to be important in the development of FCD[38]. The current study demonstrates the high prevalence of subjective cognitive decline in the absence of objective cognitive decline. Subjective cognitive decline is not an independent predictor of later neurodegeneration. Since it is associated with increased self-focused attention[39], subjective cognitive decline may be a vulnerability point from which some people go on to develop FCD, so clinicians should be aware of this condition and aim to positively diagnose it when this would be beneficial to patient management.(12) Of course there are many alternative causes of cognitive change (aside from FCD and neurodegeneration), so holistic clinical assessment remains crucial for those with problematic symptoms.

Although low-mood symptoms, and reports of poor sleep which often go alongside low mood, were found to predict sSCD, this does *not* simply imply that depression causes SCD. Most of the people in our sample did not have symptoms sufficient to qualify for a clinical diagnosis of depression, rather these are correlations between milder level symptoms. Whilst depressive symptoms correlate primarily with subjective measures of cognition, they may[40] or may not[8] lead to objective cognitive deficits. This suggests that processes interfering with meta-cognition may affect those with depression, as well as many others without depression.

Subjective cognitive decline is quite a poor indicator of recent objective cognitive decline, and does not independently predict future objective decline. These findings are relevant to the current design

For peer review only - http://bmjopen.bmj.com/site/about/guidelines.xhtml

of memory clinics, and potential changes if we enter an era of disease-modifying therapies for neurodegenerative conditions. These services would be overwhelmed if opened up to any and all people with sSCD, with little need or benefit from clinical input (and potential iatrogenic harm via heightened concern whilst awaiting clinical assessment). It is therefore important to capture objective evidence of decline, and separate features of clinical concern, to identify those more likely to benefit from specialist input.

Limitations

These data are observational, so we cannot draw strong causal inferences. CAMCOG represents a summation of different cognitive faculties; it would be helpful to be able to examine individual cognitive domains more carefully, to better understand which most closely track sSCD; and similarly, the same cognitive faculty, under different levels of attentional focus (e.g., free recall vs recognition vs implicit memory). The nature of sSCD could also be probed in more detail, such as the level of concern associated with it. This study examined older white men only, in one region of Wales. We should be cautious to generalise our results to women, ethnic minorities and other geographical populations, especially from low or middle income countries. Older white men are not the demographic associated with the highest prevalence of functional disorders reported from clinics. Nevertheless, ours is an important group to study, as we empirically don't know how common functional cognitive symptoms may be in the general population, especially since diagnostic labels may be swayed by the higher *a priori* likelihood of neurodegeneration at older ages.

Some results may be influenced by survival or loss-to-follow-up bias: only men who took part at phase 5 (average age 73) are included; their cognitive trajectories may not be representative of men who died, were lost to follow-up or too impaired to take part (though examination of death certificates and local health records was used to identify dementia cases amongst non-attenders). Nonetheless, this study gives greater insight than would a hypothetical study that only began

For peer review only

For peer review only - http://bmjopen.bmj.com/site/about/guidelines.xhtml

enrolling men at an older age (and thus never included those men from the start) and had no data on risk factors in earlier life. We tried to address loss-to-follow up at the final phase using multiple imputation, however these models may still be biased if this is informative censoring, whereby the observed outcome itself predicts missingness.(41)

Conclusions

Subjective cognitive decline is modestly associated with objective cognitive decline, but these processes appear to have largely distinct underlying mechanisms. Our data provide reassuring evidence that subjects with sSCD but without objective impairments, are not at increased risk of later CIND or dementia.

List of abbreviations:

FCD: Functional Cognitive Disorder

MCI: Mild Cognitive Impairment

SCD: Subjective Cognitive Decline

sSCD: simple Subjective Cognitive Decline (the subjective report of cognitive decline, with no other qualifiers such as score on objective tests or absence of comorbidities).

For peer review only - http://bmjopen.bmj.com/site/about/guidelines.xhtml

DECLARATIONS

Ethical approval and consent to participate

All participants gave informed consent. Ethical approval was provided by Gwent Research Ethics Committee (01/69).

Consent for publication

Not applicable.

Availability of data and materials

The datasets analysed during the current study are not publicly available due the sensitive information contained, and the nature of the consent given by the participants. The data that support the findings are available on reasonable request.

Competing interests

None of the authors declare any competing interests.

Funding

CaPS was funded by the Medical Research Council (grant G9824960-E01/1), and phase 5 was funded by a grant from the Alzheimer's Society.

Author Contributions

MF, AB, JG and YBS contributed to conception and design of the study. JG led the data collection. MF and AB established the clinical diagnoses. HB, YBS and EC planned the analyses. HB analysed the data and wrote the first draft. All authors read and approved the final manuscript.

Acknowledgements

HB is a National Institute for Health Research (NIHR) academic clinical lecturer. YBS is partially supported by the NIHR Applied Research Collaboration West (NIHR ARC West). JG is partially supported by MRC grant MR/T0333771. The views expressed in this article are those of the author(s) and not necessarily those of the NIHR or the Department of Health and Social Care.

For peer review only

For peer review only - http://bmjopen.bmj.com/site/about/guidelines.xhtml

Table 1: Relationship between change in objective cognition performance (Phase 3 to 5) and self-reported simple Subjective Cognitive Decline (sSCD) (phase 5)

	MEAN (SD)			P-VALUE	ADJUSTED R-SQUARED*
**DECLINE IN COGNITIVE TEST SCORE (ANNUAL RATE)**	No subjective cognitive decline	Subjective cognitive decline	Difference (95% CI)		
WHOLE SAMPLE					
**CAMCOG**	0.17 (0.63)	0.35 (0.91)	0.18 (0.8, 0.27)	<0.001	0.01
**AH4**	0.08 (0.61)	0.26 (0.66)	0.18 (0.09, 0.27)	<0.001	0.02
EXCLUDING THOSE WITH DEMENTIA AT PHASE 5					
**CAMCOG**	0.13 (0.55)	0.17 (0.49)	0.04 (-0.04, 0.11)	0.36	<0.001
**AH4**	0.07 (0.59)	0.22 (0.62)	0.16 (0.07, 0.24)	<0.001	0.01

*The proportion of variance in the dependent variable, that can be predicted by the independent variable

For peer review only - http://bmjopen.bmj.com/site/about/guidelines.xhtml

Table 2: The diagnostic utility of simple subjective cognitive decline (sSCD), for objective cognitive performance

	OBJECTIVE COGNITIVE DECLINE,* N(%)	NO OBJECTIVE COGNITIVE DECLINE, N(%)	P-VALUE	SENSITIVITY (95% CONFIDENCE INTERVAL)	SPECIFICITY (95% CONFIDENCE INTERVAL)
**WHOLE SAMPLE**					
	**CAMCOG +VE**	**CAMCOG -VE**			
**SSCD**	90 (9)	201 (21)	0.03	36 (30-42)	72 (68-75)
**NO SSCD**	163 (17)	515 (53)			
	**AH4 +VE**	**AH4-VE**			
**SSCD**	71 (8)	205 (22)	<0.001	44 (36-52)	73 (70-76)
**NO SSCD**	91 (10)	558 (60)			
	**CAMCOG OR AH4 +VE**	**CAMCOG OR AH4 -VE**			
**SSCD**	132 (14)	159 (16)	<0.001	38 (33-43)	74 (71-78)
**NO SSCD**	216 (22)	462 (48)			
**EXCLUDING DEMENTIA**					
	**CAMCOG +VE**	**CAMCOG -VE**			
**SSCD**	67 (7)	199 (21)	0.36	31 (25-38)	72 (69-75)
**NOSSCD**	148 (16)	513 (55)			
	**AH4 +VE**	**AH4-VE**			
**SSCD**	61 (7)	196 (22)	<0.001	42 (34-51)	74 (71-77)
**NO SSCD**	83 (9)	552 (62)			
	**CAMCOG OR AH4 +VE**	**CAMCOG OR AH4 -VE**			
**SSCD**	109 (12)	157 (17)	0.002	35 (30-41)	75 (71-78)
**NO SSCD**	201 (22)	460 (50)			

* Continuous scoring converted to a binary variable, +ve/-ve indicates whether a participant had declined more/less than 1 standard deviation from the mean rate, calculated amongst men without CIND nor dementia, between Phases 3 to 5.

sSCD = simple Subjective Cognitive decline; CAMCOG = Cambridge Cognitive Examination; AH4 = Alice Heim 4 test.

For peer review only - http://bmjopen.bmj.com/site/about/guidelines.xhtml

For peer review only

For peer review only - http://bmjopen.bmj.com/site/about/guidelines.xhtml

Table 3: Simple subjective cognitive decline and cognitive internal inconsistency, amongst people without objective cognitive decline (at phase 5). N=619.

MEN WITH NO OBJECTIVE COGNITIVE DECLINE, PHASE 5	SUBJECTIVE REPORT OF COGNITION		
**RIVERMEAD STORY RECALL TASK**	No sSCD, mean (SD)	sSCD, mean (SD)	P-value
**IMMEDIATE RECALL "IR"**	6.15 (2.82)	5.55 (2.59)	0.02
**DELAYED RECALL "DR"**	5.29 (2.78)	4.73 (2.59)	0.03
**IR-DR**	0.84 (1.45)	0.83 (1.41)	0.92
**RETENTION: DR/IR**	0.86 (0.37)	0.86 (0.35)	0.58
**RELATIVE DIFFERENCE: (IR-DR)/IR**	0.12 (0.37)	0.14 (0.35)	0.58
**CONSISTENCY OF RECALL ABILITY**	n (%)		Chi-squared P-value
**"CONSISTENT" (DR =/< IR)**	370 (80.8)	130 (82.3)	0.68
**"INCONSISTENT" (DR > IR)**	88 (19.2)	28 (17.7)	

sSCD = simple Subjective Cognitive decline

For peer review only - http://bmjopen.bmj.com/site/about/guidelines.xhtml

Table 4: Possible risk factors (phases 2 to 4) for simple Subjective Cognitive Decline (sSCD) (phase 5)

	ADJUSTED OR (95% CI) (n=601)	ADJUSTED OR (95% CI) EXCLUDING DEMENTIA (N=580)
**AGE (YEARS)**	1.12 (1.06, 1.17)	1.11 (1.05, 1.17)
**SOCIAL CLASS (MANUAL/NON-MANUAL)**	0.99 (0.65, 1.51)	0.95 (0.62, 1.46)
**PREMORBID IQ (X 0.1)**	1.06 (0.84, 1.33)	1.05 (0.83, 1.32)
**HIGH ALCOHOL USE (BINARY)**	1.07 (0.63, 1.83)	1.20 (0.70, 2.07)
**TEETOTAL (BINARY)**	0.64 (0.31, 1.32)	0.60 (0.29, 1.27)
**PROBABLE ISCHAEMIC HEART DISEASE (BINARY)**	0.98 (0.59, 1.62)	1.03 (0.61, 1.72)
**NUMBER OF VASCULAR MEDICATIONS**	1.18 (0.92, 1.50)	1.20 (0.93, 1.54)
**SMOKING (BINARY)**	0.89 (0.60, 1.34)	0.85 (0.56, 1.30)
**WAIST-HIP RATIO (X10)**	0.93 (0.66, 1.31)	0.89 (0.63, 1.27)
**POOR SLEEP**	1.52 (1.19, 1.94)	1.55 (1.21, 1.99)
**SLEEP-DISORDERED BREATHING**	1.13 (0.91, 1.40)	1.12 (0.90, 1.40)
**MOOD SYMPTOMS (GHQ SCORE: CHRONIC)***	1.27 (1.01, 1.59)	1.23 (0.97, 1.55)
**TRAIT ANXIETY (STAI)***	1.38 (1.09, 1.74)	1.39 (1.09, 1.76)
**RATE OF DECLINE CAMCOG**	1.18 (0.83, 1.69)	1.18 (0.72, 1.95)
**RATE OF DECLINE AH4**	1.33 (0.95, 1.85)	1.36 (0.97, 1.92)

*standardised

For peer review only - http://bmjopen.bmj.com/site/about/guidelines.xhtml

Table 5: sSCD does not predict future cognitive outcomes (new CIND or dementia)

**PREDICTOR AT PHASE 5: SSCD**			
**OUTCOME AT PHASE 7**	CIND or dementia	Dementia	Decline in CAMCOG between phases 5 and 7*
**PARTICIPANTS**	Excluding those with CIND or dementia at phase 5	Excluding those with dementia at phase 5	Excluding those with dementia at phase 5
**MODEL**	Odds ratio (95% CI)	Odds ratio (95% CI)	Linear coefficient (95% CI)
**COMPLETE CASE ANALYSIS**	0.92 (0.49, 1.73)	1.35 (0.61, 2.99)	0.01 (-0.13, 0.15)
**MULTIPLE IMPUTATION**	0.98 (0.56, 1.74)	1.18 (0.53, 2.61)	-0.02 (-0.19, 0.15)

All models controlled for: age, CAMCOG score at phase 5.

*The CAMCOG analysis also controlled for a dummy variable indicating CAMCOG points lost for hearing, vision, or manual dexterity at phase 7.

For peer review only - http://bmjopen.bmj.com/site/about/guidelines.xhtml

References:

1. McWhirter L, Ritchie C, Stone J, Carson A. Functional cognitive disorders: a systematic review. The Lancet Psychiatry. 2020;7(2):191-207.
2. Burmester B, Leathem J, Merrick P. Subjective Cognitive Complaints and Objective Cognitive Function in Aging: A Systematic Review and Meta-Analysis of Recent Cross-Sectional Findings. Neuropsychology Review. 2016;26(4):376-93.
3. Crumley JJ, Stetler CA, Horhota M. Examining the relationship between subjective and objective memory performance in older adults: a meta-analysis. Psychol Aging. 2014;29(2):250-63.
4. Mol ME, van Boxtel MP, Willems D, Jolles J. Do subjective memory complaints predict cognitive dysfunction over time? A six-year follow-up of the Maastricht Aging Study. Int J Geriatr Psychiatry. 2006;21(5):432-41.
5. Jessen F, Amariglio RE, van Boxtel M, Breteler M, Ceccaldi M, Chételat G, et al. A conceptual framework for research on subjective cognitive decline in preclinical Alzheimer's disease. Alzheimer's & Dementia. 2014;10(6):844-52.
6. Slot RER, Sikkes SAM, Berkhof J, Brodaty H, Buckley R, Cavedo E, et al. Subjective cognitive decline and rates of incident Alzheimer's disease and non–Alzheimer's disease dementia. Alzheimer's & Dementia. 2019;15(3):465-76.
7. Pavisic IM, Lu K, Keuss SE, James S-N, Lane CA, Parker TD, et al. Subjective cognitive complaints at age 70: associations with amyloid and mental health. Journal of Neurology, Neurosurgery & Psychiatry. 2021;92(11):1215.
8. Brailean A, Steptoe A, Batty GD, Zaninotto P, Llewellyn DJ. Are subjective memory complaints indicative of objective cognitive decline or depressive symptoms? Findings from the English Longitudinal Study of Ageing. J Psychiatr Res. 2019;110:143-51.
9. Jessen F, Amariglio RE, Buckley RF, van der Flier WM, Han Y, Molinuevo JL, et al. The characterisation of subjective cognitive decline. The Lancet Neurology. 2020.
10. Visser PJ, Verhey F, Knol DL, Scheltens P, Wahlund L-O, Freund-Levi Y, et al. Prevalence and prognostic value of CSF markers of Alzheimer's disease pathology in patients with subjective cognitive impairment or mild cognitive impairment in the DESCRIPA study: a prospective cohort study. The Lancet Neurology. 2009;8(7):619-27.
11. Eckerström M, Göthlin M, Rolstad S, Hessen E, Eckerström C, Nordlund A, et al. Longitudinal evaluation of criteria for subjective cognitive decline and preclinical Alzheimer's disease in a memory clinic sample. Alzheimer's & Dementia: Diagnosis, Assessment & Disease Monitoring. 2017;8:96-107.
12. Ball HA, McWhirter L, Ballard C, Bhome R, Blackburn DJ, Edwards MJ, et al. Functional cognitive disorder: dementia's blind spot. Brain. 2020.
13. Ganguli M, Jia Y, Hughes TF, Snitz BE, Chang C-CH, Berman SB, et al. Mild Cognitive Impairment that Does Not Progress to Dementia: A Population-Based Study. J Am Geriatr Soc. 2019;67(2):232-8.
14. Pennington C, Ball H, Swirski M. Functional Cognitive Disorder: Diagnostic Challenges and Future Directions. Diagnostics (Basel). 2019;9(4).
15. Banks J, Blundell R, Calderwood L. Wave 1- Health, Wealth and lifestyles of the older population in England: ELSA 2002. Marmot M, Banks J, Blundell R, Lessor C, Nazroo J, editors2002.
16. Mol ME, van Boxtel MP, Willems D, Verhey FR, Jolles J. Subjective forgetfulness is associated with lower quality of life in middle-aged and young-old individuals: a 9-year follow-up in older participants from the Maastricht Aging Study. Aging Ment Health. 2009;13(5):699-705.
17. Miller MA, Wright H, Ji C, Cappuccio FP. Cross-sectional study of sleep quantity and quality and amnestic and non-amnestic cognitive function in an ageing population: the English Longitudinal Study of Ageing (ELSA). PLoS One. 2014;9(6):e100991.

For peer review only - http://bmjopen.bmj.com/site/about/guidelines.xhtml

18. Fish M, Bayer AJ, Gallacher JE, Bell T, Pickering J, Pedro S, et al. Prevalence and pattern of cognitive impairment in a community cohort of men in South Wales: methodology and findings from the Caerphilly Prospective Study. Neuroepidemiology. 2008;30(1):25-33.
19. Creavin ST, Gallacher J, Bayer A, Fish M, Ebrahim S, Ben-Shlomo Y. Metabolic syndrome, diabetes, poor cognition, and dementia in the Caerphilly prospective study. J Alzheimers Dis. 2012;28(4):931-9.
20. Elwood PC, Pickering J, Bayer A, Gallacher JEJ. Vascular disease and cognitive function in older men in the Caerphilly cohort. Age and Ageing. 2002;31(1):43-8.
21. Roth M, Tym E, Mountjoy CQ, Huppert FA, Hendrie H, Verma S, et al. CAMDEX. A standardised instrument for the diagnosis of mental disorder in the elderly with special reference to the early detection of dementia. Br J Psychiatry. 1986;149:698-709.
22. Heim AW. Manual for the Group test of General Intelligence AH4. London, England: National Foundation for Educational Research.; 1955.
23. Wilson BA, Cockburn J, Baddeley AD. The Rivermead behavioural memory test. London: Thames Valley Test Company; 1985.
24. Jorm AF, Jacomb PA. The Informant Questionnaire on Cognitive Decline in the Elderly (IQCODE): socio-demographic correlates, reliability, validity and some norms. Psychol Med. 1989;19(4):1015-22.
25. von Elm E, Altman DG, Egger M, Pocock SJ, Gøtzsche PC, Vandenbroucke JP. Strengthening the Reporting of Observational Studies in Epidemiology (STROBE) statement: guidelines for reporting observational studies. Bmj. 2007;335(7624):806-8.
26. Tombaugh TN. The Test of Memory Malingering (TOMM): Normative data from cognitively intact and cognitively impaired individuals. Psychological Assessment. 1997;9(3):260.
27. Nelson HE. National adult reading test (NART) : for the assessment of premorbid intelligence in patients with dementia : test manual. Windsor, Berks: NFER-Nelson; 1982.
28. Rehm J, Hasan OSM, Black SE, Shield KD, Schwarzinger M. Alcohol use and dementia: a systematic scoping review. Alzheimers Res Ther. 2019;11(1):1-.
29. Ogliari G, Turner Z, Khalique J, Gordon AL, Gladman JRF, Chadborn NH. Ethnic disparity in access to the memory assessment service between South Asian and white British older adults in the United Kingdom: A cohort study. International Journal of Geriatric Psychiatry. 2020;35(5):507-15.
30. Liew TM, Yap P, Ng TP, Mahendran R, Kua EH, Feng L. Symptom clusters of subjective cognitive decline amongst cognitively normal older persons and their utilities in predicting objective cognitive performance: structural equation modelling. Eur J Neurol. 2019;26(9):1153-60.
31. Cosentino S, Devanand D, Gurland B. A Link between Subjective Perceptions of Memory and Physical Function: Implications for Subjective Cognitive Decline. Journal of Alzheimer's disease : JAD. 2018;61(4):1387-98.
32. Suo C, Gates N, Singh MF, Saigal N, Wilson GC, Meiklejohn J, et al. Midlife managerial experience is linked to late life hippocampal morphology and function. Brain imaging and behavior. 2017;11(2):333-45.
33. Goldstein FC, Loring DW, Thomas T, Saleh S, Hajjar I. Recognition Memory Performance as a Cognitive Marker of Prodromal Alzheimer's Disease. Journal of Alzheimer's disease : JAD. 2019;72(2):507-14.
34. Russo MJ, Campos J, Vazquez S, Sevlever G, Allegri RF. Adding Recognition Discriminability Index to the Delayed Recall Is Useful to Predict Conversion from Mild Cognitive Impairment to Alzheimer's Disease in the Alzheimer's Disease Neuroimaging Initiative. Front Aging Neurosci. 2017;9:46.
35. Oda H, Yamamoto Y, Maeda K. Neuropsychological profile of dementia with Lewy bodies. Psychogeriatrics. 2009;9(2):85-90.
36. Teodoro T, Edwards MJ, Isaacs JD. A unifying theory for cognitive abnormalities in functional neurological disorders, fibromyalgia and chronic fatigue syndrome: systematic review. Journal of Neurology Neurosurgery and Psychiatry. 2018;89(12):1308-19.

For peer review only - http://bmjopen.bmj.com/site/about/guidelines.xhtml

37. de Winter JCF, Dodou D, Hancock PA. On the paradoxical decrease of self-reported cognitive failures with age. Ergonomics. 2015;58(9):1471-86.
38. Bhome R, McWilliams A, Huntley JD, Fleming SM, Howard RJ. Metacognition in functional cognitive disorder- a potential mechanism and treatment target. Cogn Neuropsychiatry. 2019;24(5):311-21.
39. Chin J, Oh KJ, Seo SW, Na DL. Are depressive symptomatology and self-focused attention associated with subjective memory impairment in older adults? Int Psychogeriatr. 2014;26(4):573-80.
40. Schweizer S, Kievit RA, Emery T, Cam CAN, Henson RN. Symptoms of depression in a large healthy population cohort are related to subjective memory complaints and memory performance in negative contexts. Psychological medicine. 2018;48(1):104-14.
41. Dufouil C, Brayne C, Clayton D. Analysis of longitudinal studies with death and drop-out: a case study. Stat Med. 2004;23(14):2215-26.

For peer review only

For peer review only - http://bmjopen.bmj.com/site/about/guidelines.xhtml

Supplemental material for "Predictors and prognosis of population-based subjective cognitive decline: Longitudinal evidence from the Caerphilly Prospective Study (CaPS)"

Contents

Items derived from The Wisconsin Sleep Scale 1

Supplemental Figure 1: Caerphilly Prospective Study (CaPS) flowchart 2

Supplemental Figure 2: Histogram displaying delayed recall minus immediate recall ("internal inconsistency") at phase 5, according to presence or absence of simple Subjective Cognitive Decline (sSCD) 3

Supplemental Table 1: Relative frequency of possible risk variables, amongst people with and without simple Subjective Cognitive Decline (sSCD) (Phase 5) 4

Supplemental Table 2: Missingness by variable, as a percentage of n=1225 participants at phase 5 6

Supplemental table 3: possible risk factors (phases 2 to 4) for simple Subjective Cognitive Decline (phase 5), including unadjusted and imputed models 8

Items derived from The Wisconsin Sleep Scale

The Wisconsin sleep scale is mainly composed of items scored on a 1-5 point scale. These items were separated into two groups, then summed and standardized: i) *Experiencing poor sleep*, including: difficulty initiating sleep; waking overnight; difficulty getting back to sleep; not feeling rested during the day; daytime sleepiness; dozing easily in the daytime; use of caffeine or other stimulants to stay awake; and napping during the day. This comprised 8 items, (alpha 0.77). Minimum and maximum scores prior to standardisation were 8 and 39. ii) Breathing-disordered Sleep, including: frequency of snoring; loudness of snoring; awareness of snoring; making gasp/ choke/ snort noises during sleep; waking with these noises; and frequency of momentary periods in sleep where you breathe abnormally. This comprised 6 items (alpha 0.77). Minimum and maximum scores prior to standardisation were 6 and 30. Note this approach excluded items on the Wisconsin questionnaire that were either scored on a substantially different scale, or assessing other aspects of sleep: how awake you feel today; restless legs/ twitches/ kicking during sleep; nasal congestion interrupting sleep; sleep latency; reported duration of sleep (weekdays and weekends); use of sleeping tablets; and allergies causing nasal congestion.

For peer review only

Supplemental Figure 1: Caerphilly Prospective Study (CaPS) flowchart

References: (1, 2, 3)

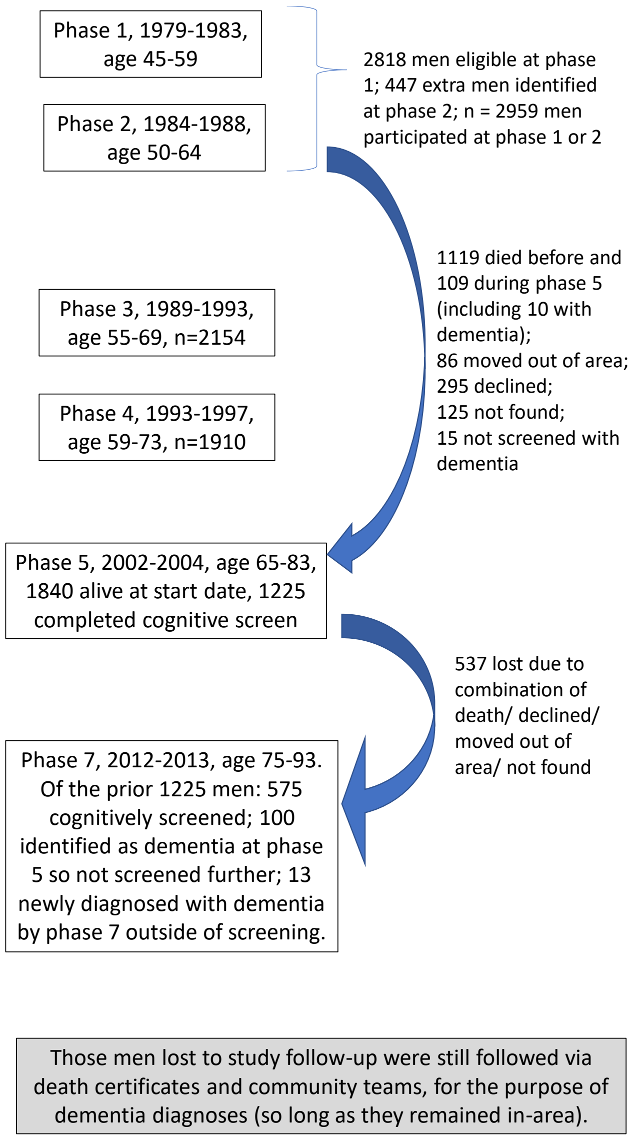

References:

1. Fish M, Bayer AJ, Gallacher JE, Bell T, Pickering J, Pedro S, et al. Prevalence and pattern of cognitive impairment in a community cohort of men in South Wales: methodology and findings from the Caerphilly Prospective Study. Neuroepidemiology. 2008;30(1):25-33.
2. Creavin ST, Gallacher J, Bayer A, Fish M, Ebrahim S, Ben-Shlomo Y. Metabolic syndrome, diabetes, poor cognition, and dementia in the Caerphilly prospective study. J Alzheimers Dis. 2012;28(4):931-9.
3. Elwood PC, Pickering J, Bayer A, Gallacher JEJ. Vascular disease and cognitive function in older men in the Caerphilly cohort. Age and Ageing. 2002;31(1):43-8.

Supplemental Figure 2: Histogram displaying delayed recall minus immediate recall ("internal inconsistency") at phase 5, according to presence or absence of simple Subjective Cognitive Decline (sSCD)

This analysis included only those men with no objective decline in measured cognition since Phase 3.

Score greater than 0 is "inconsistent". Note how roughly one fifth of men had this marker of internal inconsistency (regardless of subjective decline status).

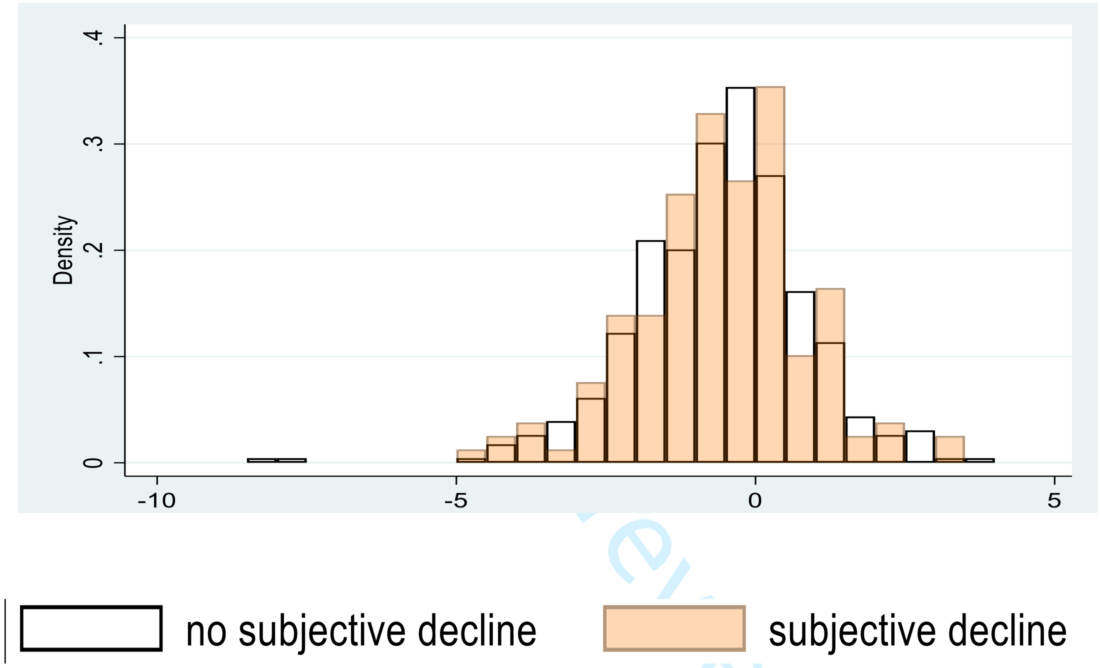

For peer review only

Supplemental Table 1: Relative frequency of possible risk variables, amongst people with and without simple Subjective Cognitive Decline (sSCD) (Phase 5)

	TOTAL N	NO SUBJECTIVE COGNITIVE DECLINE, N	SUBJECTIVE COGNITIVE DECLINE, N
TOTAL: N	1225	739	326
CIND (PHASE 5)	192	79	71
DEMENTIA (PHASE 5)	75	19	31
MANUAL SOCIAL CLASS	720	417	192
LEFT SCHOOL BEFORE 14	507	286	149
TEETOTAL (AT ANY PHASE 3-5)	120	63	37
CUMULATIVE HIGH ALCOHOL INTAKE	182	110	47
CURRENT SMOKER (ANY OF PHASE 3-5)	452	261	122
PROBABLE ISCHAEMIC HEART DISEASE	253	131	84
ANTIDEPRESSANT MEDICATION (BINARY)	15	6	8
	**ALL**	**NO SUBJECTIVE COGNITIVE DECLINE, MEAN (SD)**	**SUBJECTIVE COGNITIVE DECLINE, MEAN (SD)**
AGE (YEARS)	73.0 (4.2)	72.5 (4.0)	73.6 (4.2)
PREMORBID IQ	107.1 (9.9)	108.0 (9.9)	107.0 (9.7)
BODY MASS INDEX	26.9 (3.5)	27.0 (3.5)	26.8 (3.3)
WAIST-HIP RATIO	0.93 (0.06)	0.93 (0.06)	0.93 (0.06)
NUMBER OF MEDICATIONS FOR VASCULAR RISK	0.40 (0.83)	0.32 (0.71)	0.54 (1.05)
EXPERIENCING POOR SLEEP	-0.05 (0.95)	-0.23 (0.87)	0.33 (0.97)
BREATHING-DISORDERED SLEEP	0.02 (0.93)	-0.05 (0.88)	0.22 (0.98)
GHQ (MOOD) CHRONIC SCORING	9.0 (6.3)	7.8 (5.7)	11.3 (6.7)
STAI (TRAIT ANXIETY)	35.9 (8.9)	34.3 (8.5)	38.8 (9.3)
CAMCOG RATE OF CHANGE PER YEAR	-0.29 (0.96)	-0.17 (0.63)	-0.35 (0.91)

For peer review only

**AH4 RATE OF CHANGE PER YEAR**	-0.15 (0.64)	-0.08 (0.61)	-0.26 (0.66)

CIND = cognitive impairment, not depression; QGH = general health questionnaire (a measure of mood symptoms); STAI = Spielberger State-Trait Anxiety Inventory; CAMCOG = Cambridge Cognitive Examination; AH4= Alice Heim 4 test. *For missingness data, see Supplementary Table 1.*

Note where "no subjective cognitive decline" and "subjective cognitive decline" columns do not add up to the total, this is due to missing data. This data was missing 160/1225 (13%) for the whole cohort, 42/192 (22%) for CIND and 25/75 (33%) for dementia.

For peer review only

Supplemental Table 2: Missingness by variable, as a percentage of n=1225 participants at phase 5

VARIABLE	PHASE OF DATA COLLECTION	% MISSING
Age	Baseline	(none)
Sex	Baseline	(none, all men)
Manual social class	Baseline	4.9
Left school at 14	Baseline	10.3
STAI	2	5.3
Probable ischaemic heart disease	3	5.6
Antidepressant medication (binary)	3	Missingness data not available
Premorbid IQ	3	12.1
Body mass index	3	10.5
Waist hip ratio	3	8.2
Experiencing poor sleep	3	9.1
Sleep-disordered breathing	3	34.0
Mood symptoms (GHQ)	3	17.8
CAMCOG	3	11.6
AH4	3	12.2
CAMCOG	4	15.4
AHR	4	16.2
Teetotal	3-5	4.8
Cumulative high alcohol intake	3-5	4.8
Smoker	3-5	4.7
Number of medications for vascular risk	3	Missingness data not available
Subjective Cognitive Decline	5	13.1
CAMCOG	5	0.4
AH4	5	6.9
Immediate recall	5	2.5
Delayed recall	5	2.5
CIND or dementia	5	0.2
CAMCOG (excluding men who had dementia by phase 5)	7	56.0

For peer review only

CIND or dementia (excluding men who had dementia by phase 5)	7	53.0

For peer review only

Supplemental table 3: possible risk factors (phases 2 to 4) for simple Subjective Cognitive Decline (phase 5), including unadjusted and imputed models

	n FOR UNADJUSTED OR	UNADJUSTED OR (95% CI)	ADJUSTED OR (95% CI) (n=601)	ADJUSTED OR (95% CI), MICE (N=1225)	ADJUSTED OR (95% CI) EXCLUDING DEMENTIA (N=580)
**AGE (YEARS)**	1065	1.07 (1.03, 1.10)	1.12 (1.06, 1.17)	1.08 (1.04-1.12)	1.11 (1.05, 1.17)
**SOCIAL CLASS (MANUAL/NON-MANUAL)**	1020	1.10 (0.84, 1.45)	0.99 (0.65, 1.51)	1.07 (0.75-1.51)	0.95 (0.62, 1.46)
**PREMORBID IQ (X 0.1)**	956	0.90 (0.78, 1.03)	1.06 (0.84, 1.33)	1.09 (0.90-1.32)	1.05 (0.83, 1.32)
**HIGH ALCOHOL USE (BINARY)**	1022	0.96 (0.66, 1.39)	1.07 (0.63, 1.83)	1.08 (0.71-1.64)	1.20 (0.70, 2.07)
**TEETOTAL (BINARY)**	1022	1.37 (0.89, 2.11)	0.64 (0.31, 1.32)	1.14 (0.69-1.90)	0.60 (0.29, 1.27)
**PROBABLE ISCHAEMIC HEART DISEASE (BINARY)**	1015	1.62 (1.18, 2.22)	0.98 (0.59, 1.62)	0.94 (0.65-1.36)	1.03 (0.61, 1.72)
**NUMBER OF VASCULAR MEDICATIONS**	1065	1.33 (1.15, 1.55)	1.18 (0.92, 1.50)	1.10 (0.92-1.31)	1.20 (0.93, 1.54)
**SMOKING (BINARY)**	1022	1.10 (0.83, 1.44)	0.89 (0.60, 1.34)	1.10 (0.80-1.51)	0.85 (0.56, 1.30)
**WAIST-HIP RATIO (X10)**	990	1.19 (0.94, 1.50)	0.93 (0.66, 1.31)	1.07 (0.84-1.38)	0.89 (0.63, 1.27)
**POOR SLEEP**	983	1.91 (1.64, 2.22)	1.52 (1.19, 1.94)	1.48 (1.22-1.79)	1.55 (1.21, 1.99)
**SLEEP-DISORDERED BREATHING**	727	1.39 (1.17, 1.65)	1.13 (0.91, 1.40)	1.16 (0.95-1.42)	1.12 (0.90, 1.40)
**MOOD SYMPTOMS (GHQ SCORE: CHRONIC)***	886	1.78 (1.53, 2.06)	1.27 (1.01, 1.59)	1.38 (1.13-1.69)	1.23 (0.97, 1.55)
**TRAIT ANXIETY (STAI)***	1019	1.66 (1.44, 1.91)	1.38 (1.09, 1.74)	1.31 (1.10-1.57)	1.39 (1.09, 1.76)
**RATE OF DECLINE CAMCOG**	969	1.37 (1.13, 1.66)	1.18 (0.83, 1.69)	1.15 (0.95-1.40)	1.18 (0.72, 1.95)
**RATE OF DECLINE AH4**	925	1.59 (1.26, 1.99)	1.33 (0.95, 1.85)	1.50 (1.16-1.95)	1.36 (0.97, 1.92)

MICE = multiple imputation with chained equations

For peer review only - http://bmjopen.bmj.com/site/about/guidelines.xhtml

Reporting checklist for cohort study.

Based on the STROBE cohort guidelines.

Instructions to authors

Complete this checklist by entering the page numbers from your manuscript where readers will find each of the items listed below.

Your article may not currently address all the items on the checklist. Please modify your text to include the missing information. If you are certain that an item does not apply, please write "n/a" and provide a short explanation.

Upload your completed checklist as an extra file when you submit to a journal.

In your methods section, say that you used the STROBE cohortreporting guidelines, and cite them as:

von Elm E, Altman DG, Egger M, Pocock SJ, Gotzsche PC, Vandenbroucke JP. The Strengthening the Reporting of Observational Studies in Epidemiology (STROBE) Statement: guidelines for reporting observational studies.

		Reporting Item	Page Number
**Title and abstract**			
Title	#1a	Indicate the study's design with a commonly used term in the title or the abstract	1

For peer review only

Abstract	#1b	Provide in the abstract an informative and balanced summary of what was done and what was found	2
**Introduction**			
Background / rationale	#2	Explain the scientific background and rationale for the investigation being reported	4
Objectives	#3	State specific objectives, including any prespecified hypotheses	6
**Methods**			
Study design	#4	Present key elements of study design early in the paper	6
Setting	#5	Describe the setting, locations, and relevant dates, including periods of recruitment, exposure, follow-up, and data collection	6
Eligibility criteria	#6a	Give the eligibility criteria, and the sources and methods of selection of participants. Describe methods of follow-up.	6
Eligibility criteria	#6b	For matched studies, give matching criteria and number of exposed and unexposed	N/A
Variables	#7	Clearly define all outcomes, exposures, predictors, potential confounders, and effect modifiers. Give diagnostic criteria, if applicable	7-8
Data sources / measurement	#8	For each variable of interest give sources of data and details of methods of assessment (measurement). Describe	7-8

For peer review only

		comparability of assessment methods if there is more than one group. Give information separately for for exposed and unexposed groups if applicable.	
Bias	#9	Describe any efforts to address potential sources of bias	8-9
Study size	#10	Explain how the study size was arrived at	6
Quantitative variables	#11	Explain how quantitative variables were handled in the analyses. If applicable, describe which groupings were chosen, and why	8-9
Statistical methods	#12a	Describe all statistical methods, including those used to control for confounding	
8-9			
Statistical methods	#12b	Describe any methods used to examine subgroups and interactions	23
Statistical methods	#12c	Explain how missing data were addressed	8
Statistical methods	#12d	If applicable, explain how loss to follow-up was addressed	8
Statistical methods	#12e	Describe any sensitivity analyses	
9			

Results

For peer review only

Participants	#13a	Report numbers of individuals at each stage of study—eg numbers potentially eligible, examined for eligibility, confirmed eligible, included in the study, completing follow-up, and analysed. Give information separately for for exposed and unexposed groups if applicable.	Figure 1
Participants	#13b	Give reasons for non-participation at each stage	Figure 1
Participants	#13c	Consider use of a flow diagram	
Figure 1			
Descriptive data	#14a	Give characteristics of study participants (eg demographic, clinical, social) and information on exposures and potential confounders. Give information separately for exposed and unexposed groups if applicable.	Table 1
Descriptive data	#14b	Indicate number of participants with missing data for each variable of interest - Supplementary Table 1	
Descriptive data	#14c	Summarise follow-up time (eg, average and total amount)	
Figure 1			
Outcome data	#15	Report numbers of outcome events or summary measures over time. Give information separately for exposed and unexposed groups if applicable.	

Page 11

For peer review only

Main results	#16a	Give unadjusted estimates and, if applicable, confounder-adjusted estimates and their precision (eg, 95% confidence interval). Make clear which confounders were adjusted for and why they were included	Tables
Main results	#16b	Report category boundaries when continuous variables were categorized	7
Main results	#16c	If relevant, consider translating estimates of relative risk into absolute risk for a meaningful time period	
N/A			
Other analyses	#17	Report other analyses done—eg analyses of subgroups and interactions, and sensitivity analyses	Tables
**Discussion**			
Key results	#18	Summarise key results with reference to study objectives	12
Limitations	#19	Discuss limitations of the study, taking into account sources of potential bias or imprecision. Discuss both direction and magnitude of any potential bias.	14-15
Interpretation	#20	Give a cautious overall interpretation considering objectives, limitations, multiplicity of analyses, results from similar studies, and other relevant evidence.	12-16
Generalisability	#21	Discuss the generalisability (external validity) of the study results	15

For peer review only

Other Information

Funding	#22	Give the source of funding and the role of the funders for the present study and, if applicable, for the original study on which the present article is based	17

None The STROBE checklist is distributed under the terms of the Creative Commons Attribution License CC-BY. This checklist can be completed online using https://www.goodreports.org/, a tool made by the EQUATOR Network in collaboration with Penelope.ai

For peer review only

BMJ Open

Predictors and prognosis of population-based subjective cognitive decline: Longitudinal evidence from the Caerphilly Prospective Study (CaPS)

Journal:	*BMJ Open*
Manuscript ID	bmjopen-2023-073205.R2
Article Type:	Original research
Date Submitted by the Author:	03-Sep-2023
Complete List of Authors:	Ball, Harriet; University of Bristol, Population Health Sciences<br>Coulthard , Liz ; University of Bristol Medical School, Translational Health Sciences<br>Fish, Mark; Royal Devon University Healthcare NHS Foundation Trust<br>Bayer, Antony; Cardiff University, Institute of Primary Care and Public Health<br>Gallacher, John; Oxford<br>Ben-Shlomo, Yoav; University of Bristol, Population Health Sciences
**Primary Subject Heading**:	Epidemiology
Secondary Subject Heading:	Mental health, Neurology
Keywords:	Dementia < NEUROLOGY, Dementia, Delirium & cognitive disorders < PSYCHIATRY, EPIDEMIOLOGY

SCHOLARONE™
Manuscripts

I, the Submitting Author has the right to grant and does grant on behalf of all authors of the Work (as defined in the below author licence), an exclusive licence and/or a non-exclusive licence for contributions from authors who are: i) UK Crown employees; ii) where BMJ has agreed a CC-BY licence shall apply, and/or iii) in accordance with the terms applicable for US Federal Government officers or employees acting as part of their official duties; on a worldwide, perpetual, irrevocable, royalty-free basis to BMJ Publishing Group Ltd ("BMJ") its licensees and where the relevant Journal is co-owned by BMJ to the co-owners of the Journal, to publish the Work in this journal and any other BMJ products and to exploit all rights, as set out in our licence.

The Submitting Author accepts and understands that any supply made under these terms is made by BMJ to the Submitting Author unless you are acting as an employee on behalf of your employer or a postgraduate student of an affiliated institution which is paying any applicable article publishing charge ("APC") for Open Access articles. Where the Submitting Author wishes to make the Work available on an Open Access basis (and intends to pay the relevant APC), the terms of reuse of such Open Access shall be governed by a Creative Commons licence – details of these licences and which Creative Commons licence will apply to this Work are set out in our licence referred to above.

Other than as permitted in any relevant BMJ Author's Self Archiving Policies, I confirm this Work has not been accepted for publication elsewhere, is not being considered for publication elsewhere and does not duplicate material already published. I confirm all authors consent to publication of this Work and authorise the granting of this licence.

For peer review only

Predictors and prognosis of population-based subjective cognitive decline: Longitudinal evidence from the Caerphilly Prospective Study (CaPS)

Harriet Ball[1], Elizabeth Coulthard[2], Mark Fish[3], Antony Bayer[4], John Gallacher[5], Yoav Ben-Shlomo[1].

1. Population Health Sciences, Bristol Medical School, University of Bristol, UK
2. Translational Health Sciences, Bristol Medical School, University of Bristol, UK
3. Royal Devon University Healthcare Trust, Exeter, UK
4. Division of Population Medicine, Cardiff Medical School, Cardiff University, UK
5. Department of Psychiatry, University of Oxford, UK

Corresponding author: Harriet Ball, harriet.ball@bristol.ac.uk Southmead Hospital Learning and Research Building, Bristol Medical School, University of Bristol.

Word count: 5,027

For peer review only

Article summary

Objectives: to understand associations between the subjective experience of cognitive decline, and objective cognition. This subjective experience is often conceptualised as an early step towards neurodegeneration, but this has not been scrutinised at the population level. An alternative explanation is poor meta-cognition, the extreme of which is seen in Functional Cognitive Disorder (FCD).

Design: prospective cohort (Caerphilly Prospective Study)

Setting: population-based, South Wales, UK

Participants: This men-only study began in 1979; 1225 men participated at average age 73 in 2002-2004, including assessment of simple Subjective Cognitive Decline (sSCD, defined as subjective report of worsening memory or concentration). Dementia outcomes were followed up to 2012-2014. Data on non-completers was additionally obtained from death certificates and local health records.

Primary and Secondary Outcome Measures: The primary outcome measure was incident dementia over 10 years. Secondary outcome measures included prospective change in objective cognition, and cross-sectional cognitive internal inconsistency (the existence of a cognitive ability at some times, and its absence at other times, with no intervening explanatory factors except for focus of attention).

Results: sSCD was common (30%) and only weakly associated with prior objective cognitive decline (sensitivity 36% [95% CI 30-42], specificity 72% [95% CI 68-75]). Independent predictors of sSCD were older age, poor sleep quality and higher trait anxiety. Those with sSCD did not have excess cognitive internal inconsistency, but results suggested a mild attentional deficit. sSCD did not predict future objective cognitive change (linear regression coefficient -0.01 (95% CI -0.13, 0.15)).

Conclusions: sSCD is weakly associated with prior objective cognitive decline, and does not predict future cognition. Prior sleep difficulties and anxiety were the most robust predictors of sSCD. sSCD in

For peer review only - http://bmjopen.bmj.com/site/about/guidelines.xhtml

the absence of objective decline appears to be a highly prevalent example of poor meta-cognition (i.e., poor self-awareness of cognitive performance), which could be a driver for later FCD.

Strengths and limitations of this study

- The design was prospective and population-based, with detailed collection of many potential risk or confounding factors, from middle to older age
- Outcome measures included objective test scores, and clinical dementia diagnoses (including local clinic and death certificate records for those who did not participate to the end)
- The study only included men from the outset, so we cannot extrapolate results to women

Funding: CaPS was funded by the Medical Research Council (grant G9824960-E01/1), and phase 5 was funded by a grant from the Alzheimer's Society.

Competing interests: None of the authors declare any competing interests.

Keywords: Dementia, subjective cognitive decline, functional cognitive disorder

For peer review only - http://bmjopen.bmj.com/site/about/guidelines.xhtml

BACKGROUND

We know that cognitive symptoms are common, and many people who attend memory clinics due to cognitive symptoms turn out not to have a progressive cognitive disorder.(1) The link between subjective reports and objective evidence of cognitive decline is surprisingly weak,(2-4) or sometimes found only in subgroups. An international working group (SCD-I) has published criteria for "pre-MCI Subjective Cognitive Decline" as those who experience persistent decline in cognitive ability relative to their baseline; despite scoring in normal ranges on objective cognitive tests; and excluding those meeting criteria for Mild Cognitive Impairment (MCI) or dementia, that explained by psychiatric or neurologic disease (except AD), medical disorder, medication or substance abuse.(5) This definition has been created to harmonise research exploring evidence of neurodegeneration. However, this definition excludes many in the population with new-onset subjective experience of cognitive decline (which is important since this experience, regardless of the exclusions in the definition, drives their concern and potential presentation to medical services). We define *simple* subjective cognitive decline (sSCD) as purely the subjective experience, irrespective of objective cognition or medical or psychiatric disorders.

A multi-cohort study found the risk of dementia in those with SCD (as defined by the SCD-I) was driven very largely by clinic-based rather than community or population-based samples.(6) Similarly, population-level association between SCD and amyloid status is masked once controlling for anxiety and demographic variables.(7) In the English Longitudinal Study of Ageing (ELSA) cohort,(8) although objective cognitive changes predicted subsequent worsening in subjective cognition, deterioration in subjective cognition did not prospectively predict objective cognition. Therefore, isolated subjective cognitive problems *often do not* herald the start of a progressive neurodegenerative process. Research attention is now focusing on a narrower group who have extra clinical features ("SCD-plus"), but who are yet further from the common population experience of subjective worsening in cognitive ability. "SCD-plus" refers to Subjective Cognitive Decline plus at least one additional

For peer review only - http://bmjopen.bmj.com/site/about/guidelines.xhtml

For peer review only

feature including: amnestic rather than non-amnestic complaints; onset <5 years ago; onset >age 60 years; concern associated with SCD; persistence of SCD over time; help-seeking; confirmation of decline by an informant).(9) “SCD plus” has been associated with a higher risk of developing objective cognitive deficits,(10) or frank dementia, than those with SCD alone.(11) But it is also likely that “SCD plus” would be over-represented in clinic populations relative to those in the general population.

The aim of this paper is to explore population-based associations with the subjective experience of cognitive decline (sSCD), since outcomes are likely to be different than when examining clinic-based populations that are less representative. The latter are likely to have worse outcomes, which might make more aggressive investigation and potential treatment appropriate, but may not hold for the general population with subjective cognitive problems. This is important to consider since the typical cases seen currently in clinic may change to include more people in the light of novel therapies and wider public awareness of neurodegenerative problems. This paper also takes a more nuanced and dimensional view of any association with objective cognitive change, noting individual differences in test scores rather than whether someone is just above versus just below a cutoff for “abnormal”.

A subjective experience of cognitive difficulty that outstrips objective evidence, when severe and impairing, is a key feature of Functional Cognitive Disorder(12); but a milder experience might be common in the wider population. Functional symptoms (which can wax and wane) therefore might explain *some* of the “recovery” of people with scores in the Mild Cognitive Impairment range to the normal range (up to one third in community based samples [13]). Another key features of FCD is cognitive “internal inconsistency”, which is when someone struggles to perform a cognitive ability, for which they are demonstrably capable, without any external factor intervening, aside from perhaps a change in focus of attention.(12, 14)

For peer review only - http://bmjopen.bmj.com/site/about/guidelines.xhtml

Population-based analyses show subjective cognitive concerns are more common in people who are older, of lower social class and less educated[15]; and are associated with worse quality of life, and higher anxiety[16]. Sleep disturbance is associated with objective cognitive dysfunction, more so at older ages[17], but less has been reported about its association with subjective cognition.

The current paper uses a prospective cohort of men in Wales, with in-depth cognitive assessments, and data spanning over three decades, to examine the prevalence of sSCD, its relation to objective cognitive change, and prospective associations with potential causative factors. Our research questions were:

1) Is sSCD is a good indicator of prior objective cognitive decline? This is relevant because clinicians might put more weight on a report of subjective cognitive decline, than an objective test score in a broadly normal range.
2) Do those with sSCD in the absence of objective decline, also have features of cognitive internal inconsistency?
3) Does sSCD have different risk factors compared to those for objective cognitive decline ?
4) Is sSCD associated with risk of future objective cognitive decline or dementia?

METHODS

The Caerphilly Prospective Study (CaPS) began in 1979 (Supplemental Figure 1) (18-20). Men aged 45-59 were identified from electoral rolls and general practice lists in Caerphilly and surrounding villages in South Wales, UK. Women were excluded as the initial focus was on cardiovascular health and it was felt that too few events would be accrued in the early follow-up. Beginning in phase 3, (age 55-69 years) cognitive assessments were added including the Cambridge Cognitive Examination (CAMCOG)[21], the Alice Heim 4 test (AH4)[22] and the Rivermead Behavioural Memory Test (RBMT,[23] which includes immediate recall of a 21-clause story, and delayed recall after 20 minutes). Cognitive assessments were also repeated at Phases 4 and 5. At Phase 5 (mean age 73 years), a subset of

For peer review only - http://bmjopen.bmj.com/site/about/guidelines.xhtml

participants (those who could not complete the CAMCOG, or scored 82 or fewer, or showed at least a 10 point decline over time) were invited to a clinical assessment to identify those who had dementia, and those who had “cognitive impairment not dementia” (CIND, which includes those with MCI). Informants (e.g., spouses or children) also rated change in participants’ day to day abilities (IQCODE(24)).

A further assessment of *likely* dementia status was made holistically at Phase 7, by a cognitive disorders clinician (AB), using all available information, including longitudinal CAMCOG score, IQCODE, and all medical records. Those who had previously been classified as having dementia at Phase 5 were assumed to still have this diagnosis and were not re-evaluated. By this phase (mean age 82 years), some men had other health problems making it difficult to complete the cognitive assessments, leading to altered scores related to factors such as hearing, vision or manual dexterity. We have followed the STROBE cohort reporting guidelines.(25)

In assessing decline in objective cognitive test scores from Phases 3 to 5, we focused firstly on an instrument typically used as a dementia screen (CAMCOG), and secondly a scale more often used to indicate reasoning skills in the general population (AH4). The mean score at Phases 3 and 4 (or just one if the other was missing), was subtracted from the score at Phase 5, and divided by the time interval, to indicate rate of change over time (years). We defined “objective cognitive decline” as a decline of one standard deviation below the mean decline, of those men who had neither dementia nor CIND at Phase 5. Although an arbitrary cutoff, this value was chosen to capture a potentially subtle difference from an expected average decline over 8-12 years which is unlikely to be due to merely random error; with the benefit of premorbid detailed cognitive assessment, this difference might be smaller than what is felt to be a meaningful decline in a clinical setting.

At Phase 5, men were asked about their subjective cognition via two questions: In the last 12 months my *memory/concentration* (both asked independently) has: improved, not changed, got a

For peer review only - http://bmjopen.bmj.com/site/about/guidelines.xhtml

little worse, got a lot worse. These we recoded as 0=improved/no change, 1=little worse, 2=lot worse to produce (0-2) scores for memory and concentration. Scores on these two items were correlated (Spearman rho 0.64, p<0.001) and we combined them to generate a total score (0-4). We also created a binary variable (sSCD) defined as a score of 2 or more out of 4. Therefore our definition of SCD is more basic than SCD as defined earlier by the international working group.

"Cognitive internal inconsistency" was not a focus of this study at the time of data collection. Performance validity tests exist, but are protracted and were not performed as part of this study; further, the literature around these tests mainly focus on identification of conscious malingering, eg the TOMM.(26) We therefore looked for ways to identify unambiguous cognitive internal inconsistency in routinely collected data. We pragmatically operationalised this as delayed recall being paradoxically higher than immediate recall on the RBMT story recall task (i.e., looking at a memory component of internal inconsistency), and compared the frequency of this across those with and without sSCD. This is very far from a definitive test for FCD, but we would expect it to be present more often if a group included an excess of people with FCD. Due to the constraints of the data collected, it was not possible to identify a similarly unambiguous signal for cognitive components other than memory.

We considered the following variables as important confounders: age, social class (manual vs non-manual), years of education (before or after age 14 years) and premorbid IQ proxied by the National Adult Reading Test (NART)[27]. We assessed neurodegenerative risk markers at phases 2 to 4. These included probable ischaemic heart disease (assessed through hospital records and ECG changes), BMI and waist-hip ratio, smoking status, and medication history indicating vascular risk (i.e., medications whose indications were unequivocally for blood pressure, vascular disease, diabetes, or lipid-lowering). Alcohol exposure was indexed both as a binary marker of avoidance ("teetotal" at *any* of phases 2 through 4), and a binary indicator of the highest cumulative alcohol

For peer review only - http://bmjopen.bmj.com/site/about/guidelines.xhtml

use at Phases 2 through 4 (those drinking an average of 4 or more units per day across the period), due to previous work suggesting a non-linear association between alcohol and cognition[28]. At phase 3 we also assessed mood symptoms, using the chronic scoring of the General Health Questionnaire, the Spielberger State-Trait Anxiety Inventory, and use of any anti-depressant medication. Sleep problems were assessed using items from the Wisconsin Sleep Questionnaire, divided into "experiencing poor sleep" and "breathing-disordered sleep" (see Supplemental material).

Statistical analysis plan

The present study includes men who took part at phase 5, including the cognitive assessments (*n*=1,225). We calculated the prevalence of sSCD at phase 5 as the number of cases divided by the population at risk. We compared objective cognitive decline between phases 3 and 5, either as a continuous score (t-test) or binary measure "objective cognitive decline" (chi-squared test, using CAMCOG and AH4 separately) for those with and without sSCD at phase 5. We also derived the sensitivity and specificity of sSCD in relation to objective decline (Yes/No) as the gold standard. Amongst those with no objective cognitive decline by phase 5, we tested for an association between sSCD and cognitive internal inconsistency, using chi-squared test. We ran multivariable logistic regression models to identify possible causal contributors (measured at phases 2-4) to sSCD (at phase 5). We tested whether sSCD (at phase 5) predicted either a decline in CAMCOG (linear regression), or worsening cognitive severity category (logistic regression), between phases 5 and 7.

To address the potential bias when undertaking complete case analysis of potential causal contributors to sSCD, we included multiple imputation with chained equations (MICE) and pooled estimates using Rubin's rules across 50 imputed datasets. The imputation model used all the variables included in the analysis model (i.e., all the potential predictors of sSCD, as well as sSCD itself). To address potential survivor bias, we included a MICE model when looking at phase 7 outcomes, using the same principles and the same imputed variables as in the aforementioned

For peer review only - http://bmjopen.bmj.com/site/about/guidelines.xhtml

For peer review only

imputation, except we additionally included all variables that were in the phase 7 analysis model, in the imputation model.

As sensitivity analyses, we repeated the above excluding those with a pre-existing diagnosis of dementia. This is because in a clinical setting, it is usually relatively straightforward to identify people whose cognitive impairment is severe enough to be categorised as dementia; the diagnostic difficulty lies in understanding causes amongst those whose cognitive difficulty is milder. In addition, it excludes those who might have missing data on items about subjective cognition due to being severely cognitively impaired (which could introduce bias). We also repeated analyses looking at the memory-only versus concentration-only aspects of subjectively reported cognition. We performed a post hoc power calculation, though this is controversial and may be misleading.(29) Analyses were conducted in STATA v16.

Patient and public involvement

This study began in the 1970s when it was not normal practice to involve participants in the design and co-production of research. However, the researchers kept the participants well informed of the research progress and key findings through regular newsletters and including a 25th follow-up birthday party where all survivors met the research team and the Welsh Minister of Health. Over the study duration, ad hoc feedback has occurred with study participants writing or speaking to the research team with their views about the study. For example, a sensitive question was dropped based on participants' comments.

RESULTS

Sociodemographics, and frequency of risk variables

1,225 men took part at Phase 5 including completion of cognitive assessments, with mean age of 73 (range 65-83) years (Supplemental Figure 1). 62% of men (n=720) were of manual social class. 46%

For peer review only - http://bmjopen.bmj.com/site/about/guidelines.xhtml

(n=507) had left school by age 14 (in later analyses, we use NART-IQ as a more normally distributed proxy for amount of education received). 75 (6.1%) were assessed as having dementia, and 192 (15.7%) as having CIND. 326 participants (30.6%) had sSCD. Unadjusted, subjects with sSCD were more likely to have CIND, Dementia, lower education, probable IHD, be older, report worse sleep, have a worse GHQ score, and show slightly greater declines in their CAMCOG and AH4 scores (Supplemental Table 1). sSCD status was missing for 160/1225 (13%) amongst the whole cohort, 42/192 (22%) amongst those with CIND, and 25/75 (33%) amongst those with dementia. Only 15 men (1.2%) were on antidepressant medication; due to low prevalence, this variable was not analysed further.

Of those who did not already have dementia at Phase 5, 37/573 (6.5%) had possible dementia by phase 7. Of those who did not already have CIND at phase 5, 84/528 (15.9%) had CIND or dementia by phase 7. Of those who were classified as not having dementia at phase 5, 575/1148 (50.1%) had missing data on this measure at phase 7.

Question 1: Is subjective cognitive decline (at phase 5) a good indicator of prior objective cognitive decline?

Objective CAMCOG cognitive change was scored continuously, and the average drop (from the baseline of Phase 3 or 4, to Phase 5) was 2.7 points per year in those developing dementia, 0.7 per year in those developing CIND, and 0.06 per year in those without either by Phase 5. The equivalent figures for AH4 were 0.9, 0.2 and 0.1 respectively. Objective changes in cognition were slightly worse in those with sSCD (Table 1), but the proportion of variance explained was very low. These associations were attenuated when excluding those with dementia, and this (unadjusted) association remained statistically significant for AH4 (but not for CAMCOG).

For peer review only - http://bmjopen.bmj.com/site/about/guidelines.xhtml

Of people who reported sSCD, the majority did not have objective cognitive decline as defined by >=1 standard deviation decline (Table 2). The utility for sSCD as a screening tool for objective decline was poor with a sensitivity of between 36-44% and specificity of 72-75%.

Question 2: Do those with subjective cognitive decline but no objective decline (at phase 5), have features of cognitive internal inconsistency?

In the Rivermead story recall test, subjects reporting sSCD did slightly worse than others on immediate recall (5.55 versus 6.15, p=0.02), and then proportionately worse on delayed recall (4.73 versus 5.29, p=0.03). The worse score on delayed recall was in proportion to the worse performance on immediate recall and more reflective of a mild attentional deficit than a true amnestic difficulty. There was no evidence of excess cognitive internal inconsistency (indicated by a higher score on delayed than immediate recall) when comparing those with and without sSCD (0.83 vs 0.84, p=0.92) (Table 3 and Supplemental Figure 2).

Question 3: Which characteristics predict subjective cognitive decline (at phase 5), and are these different to risk factors traditionally linked to objective cognitive decline

Variables that predicted sSCD in unadjusted logistic models included: age; probable ischaemic heart disease; vascular medications; experiencing poor sleep; breathing-disordered sleep; mood symptoms; trait anxiety; rate of decline in CAMCOG; and rate of decline in AH4 (Table 4, and Supplemental Table 3). There was no unadjusted association with social class, premorbid IQ, alcohol use (either high use or teetotal status), tobacco exposure, BMI, waist-hip ratio, nor baseline measures of CAMCOG and AH4. However, in a model simultaneously adjusting for all these factors, the only significant independent predictors remaining were: age, experiencing poor sleep, mood symptoms, and trait anxiety (respective ORs: 1.12, 1.52, 1.27 and 1.38). These associations (except mood symptoms, which became borderline non-significant) remained when excluding participants with a clinical diagnosis of dementia at phase 5.

For peer review only - http://bmjopen.bmj.com/site/about/guidelines.xhtml

Due to missing data, the adjusted complete case analysis only included 601 participants. This model, but with multiple imputation (n=1,225), found the same significant independent predictors, with the addition of one variable (rate of decline in AH4, OR 1.50, 1.16-1.95, see Supplemental Table 3).

Question 4: Does subjective cognitive decline (phase 5) predict future CIND and dementia (phase 7)?

sSCD at phase 5 did not predict worsening cognition at phase 7 (Table 5), when examined either by diagnosis (new CIND or new dementia) or by change in CAMCOG score. Due to attrition to phase 7, this was assessed both by complete case analysis and multiple imputation.

Sensitivity analyses and post hoc power calculation

Results of analyses excluding those with a diagnosis of dementia are reported above.

We replaced overall sSCD with reported complaints in either memory-only or concentration-only. These showed the same patterns of association with measures of objective cognition, internal inconsistency (lack of association), possible confounders and risk factors, and future dementia (lack of association).

In testing the hypothesis that sSCD may or may not predict the combined outcome of incident CIND and dementia, we ran several power calculations under the assumption of 5%, 7.5% and 10% increased risk for the sSCD group over the global risk. The ratio of sSCD to non-sSCD was 0.44 (at phase 5), and the proportion of people who newly developed CIND or dementia cases (between phases 5 and 7) was 0.21. The power for these two-sample comparisons, using a two-sided alpha of 0.05, were respectively: 0.54, 0.84 and 0.99.

DISCUSSION

For peer review only - http://bmjopen.bmj.com/site/about/guidelines.xhtml

In this population-based sample of 1,225 older men in South Wales, we found only a weak association between objective cognitive change and subjective report of that same change, partially accounted for by some of the subjective group having dementia. Those who had subjective cognitive concerns without objective cognitive decline, nonetheless, showed a mild deficit in attentional processes. At the population level, sSCD has low sensitivity and specificity to identify objective evidence of cognitive decline (both missing many people who lack insight into their cognitive problems; and detecting people who are concerned in the absence of objective problems). Independent predictors for sSCD included preceding anxiety or mood symptoms, and poor self-reported sleep, as well as older age, but not other variables known to be risk factors for neurodegeneration. sSCD, adjusted for objective cognition, did not predict later CIND or dementia.

This is the first large population-based cohort study of subjective experience of cognitive decline which can look at both predictors as well as future clinical outcomes, with a wide range of potential confounders measured at younger ages, and hence is less prone to bias and reverse causation. We found no prospective association between subjective cognitive decline and later objective cognitive decline (either in numerical score or severity category i.e., CIND or dementia). Naturally, a larger sample size would have given greater power to detect a more modest effect. However, this negative finding suggests that subjective cognitive symptoms, by themselves, are not substantially (on a population level) the manifestation of a neurodegenerative condition. We found a weak association between prior objective cognitive decline and subjective report of decline in cognition. The interpretation of this association is complex since those with severely impaired cognition might lack insight or even not understand the question, but also there are a large number of people in the general population who feel their cognition is suboptimal. Previous studies that have found a more convincing association have largely been clinic-based. The reasons for this discrepancy likely include differences in populations according to who seeks and can access clinic assessment (which could include socioeconomic differences, age, comorbidities and ethnicity for example)(30). Most clinic-

For peer review only

For peer review only - http://bmjopen.bmj.com/site/about/guidelines.xhtml

based studies of SCD often have participants with “SCD-plus” (i.e., extra factors, such as partner concern, that make neurodegeneration more likely and may increase their chances of reaching clinic). The choice of test and cut-off, and cross-sectional versus decline measure, can also affect results. Our decline measure is potentially a quite sensitive measure of change over time, given that we had a premorbid baseline measure, which is not present in clinic-based studies that begin when patients already present. The exact question framing also matters regarding self-reported cognitive difficulties, since some items are more likely to index normal ageing, and others pathological changes.(31-33)

Of our two objective cognitive tests, AH4 showed a slightly more robust association with sSCD than did CAMCOG (AH4 assesses complex verbal and numerical reasoning, analogous to an IQ test(22); CAMCOG measures faculties more commonly assessed in the dementia clinic(21)). Perhaps the population variance in complex reasoning more closely mirrors peoples’ understanding of what it is to have memory or concentration problems. Alternatively, perhaps AH4 captures more meaningful variance in the normal range of cognition, whereas CAMCOG is better at detecting the more severe end (where those with cognitive difficulties often lack insight). Either way, it may be useful to incorporate measures similar to AH4 into neuropsychometry batteries for patients with suspected functional neurological symptoms, as this could represent a measure capable of tracking progress, providing a useful discussion point with patients.

Amongst men with subjective cognitive symptoms despite a lack of objective cognitive decline, we found they fared slightly worse (compared to those without cognitive symptoms) in a task of immediate recall, but no worse than expected on delayed recall of the initially remembered items. This reinforces the suggestion that SCD is linked to a slight deficit in attentional processes (despite not having dipped on overall cognitive test scores over many years). This pattern is distinct from the amnestic processes typically seen in early Alzheimer’s disease(34, 35) or the attentional problems

For peer review only

alongside wider progressive cognitive problems seen in dementia with Lewy bodies[36]. It is also of note that this group did not exhibit cognitive internal inconsistency (no evidence of scoring *better* on delayed recall than on immediate recall). This is not entirely surprising, as our group are quite distinct from clinic-based patients with Functional Cognitive Disorder in whom this characteristic is a key part of diagnosis(12) (and in whom more than a one-off example of evidence of internal inconsistency should be sought). Attentional problems have been identified in other conditions within the umbrella of functional somatic syndromes, when such patients have been tested cognitively (37). It is also important to recognise that our pragmatic measure of "cognitive internal inconsistency" is identifiable in approximately one fifth of normal healthy people (Supplemental Figure 2), a surprisingly high number if only taking this one snapshot measurement into account.

We found that sSCD was more common at older ages. The same has been found in individual studies where a broad age range is considered[8]. When comparing across studies of people of differing ages, this association is less robust(1), probably in part due to heterogeneity between included studies. Reasons for this association could include differences in meta-cognitive processes by age; older people resigning to using work-arounds; age-related cultural expectations; or the way questions are framed leading to self-rating against different comparator groups; as well as some loss of insight when objective cognitive deficits take over[38]. The dip in subjective cognition with age may reflect the recognised small dip in objective cognitive abilities at older ages, as well as higher prevalence of neurodegeneration. However, factors typically associated with neurodegenerative change (vascular health, alcohol use) did not predict later sSCD in the fully adjusted model. This also held in an analysis excluding people with dementia. We interpret this to indicate that subjective cognitive decline is predicted by objective cognitive decline and its determinants, but also has important predictors independent of objective cognition. These predictors included prior reports of poor sleep quality, and prior anxiety symptoms. This supports the proposition that anxiety and poor sleep may be contributing to sSCD (rather than being a by-product of worry about cognitive change). These risk

For peer review only - http://bmjopen.bmj.com/site/about/guidelines.xhtml

factors for sSCD may also predict functional cognitive symptoms. It is also notable that anxiety and poor sleep are relevant in our study which consisted of older males (whereas the literature and clinical diagnoses of functional disorders focus more on younger females). The association between subjective and objective cognition may be different in women, in particular since women may have an extra mid-life peak of subjective cognitive concern.(15)

Subjective reports of poor sleep often go alongside sSCD, however when using actigraphy(an objective assessment of sleep quality), the association is less or even reversed [1]. There are likely qualitatively different sleep problems amongst those with sSCD, compared to those with organic changes to sleep in early dementia.

Clinical implications

Subjectively experiencing cognitive decline in the absence of an objective decline, is a marker of poor meta-cognition (i.e. poor ability to reflect on and monitor one's own cognitive abilities). This is a process postulated to be important in the development of FCD[39]. The current study demonstrates the high prevalence of subjective cognitive decline in the absence of objective cognitive decline. Subjective cognitive decline is not an independent predictor of later neurodegeneration. Since it is associated with increased self-focused attention[40], subjective cognitive decline may be a vulnerability point from which some people go on to develop FCD, so clinicians should be aware of this condition and aim to positively diagnose it when this would be beneficial to patient management.(12) Of course there are many alternative causes of cognitive change (aside from FCD and neurodegeneration), so holistic clinical assessment remains crucial for those with problematic symptoms.

Although low-mood symptoms, and reports of poor sleep which often go alongside low mood, were found to predict sSCD, this does *not* simply imply that depression causes SCD. Most of the people in

For peer review only - http://bmjopen.bmj.com/site/about/guidelines.xhtml

our sample did not have symptoms sufficient to qualify for a clinical diagnosis of depression, rather these are correlations between milder level symptoms. Whilst depressive symptoms correlate primarily with subjective measures of cognition, they may[41] or may not[8] lead to objective cognitive deficits. This suggests that processes interfering with meta-cognition may affect those with depression, as well as many others without depression.

Subjective cognitive decline is quite a poor indicator of recent objective cognitive decline, and does not independently predict future objective decline. These findings are relevant to the current design of memory clinics, and potential changes if we enter an era of disease-modifying therapies for neurodegenerative conditions. These services would be overwhelmed if opened up to any and all people with sSCD, with little need or benefit from clinical input (and potential iatrogenic harm via heightened concern whilst awaiting clinical assessment). It is therefore important to capture objective evidence of decline, and separate features of clinical concern, to identify those more likely to benefit from specialist input.

Limitations

These data are observational, so we cannot draw strong causal inferences. CAMCOG represents a summation of different cognitive faculties; it would be helpful to be able to examine individual cognitive domains more carefully, to better understand which most closely track sSCD; and similarly, the same cognitive faculty, under different levels of attentional focus (e.g., free recall vs recognition vs implicit memory). The nature of sSCD could also be probed in more detail, such as the level of concern associated with it. This study examined older white men only, in one region of Wales. We should be cautious to generalise our results to women, ethnic minorities and other geographical populations, especially from low or middle income countries. Older white men are not the demographic associated with the highest prevalence of functional disorders reported from clinics. Nevertheless, ours is an important group to study, as we empirically don't know how common

For peer review only - http://bmjopen.bmj.com/site/about/guidelines.xhtml

functional cognitive symptoms may be in the general population, especially since diagnostic labels may be swayed by the higher *a priori* likelihood of neurodegeneration at older ages.

Some results may be influenced by survival or loss-to-follow-up bias: only men who took part at phase 5 (average age 73) are included; their cognitive trajectories may not be representative of men who died, were lost to follow-up or too impaired to take part (though examination of death certificates and local health records was used to identify dementia cases amongst non-attenders). Nonetheless, this study gives greater insight than would a hypothetical study that only began enrolling men at an older age (and thus never included those men from the start) and had no data on risk factors in earlier life. We tried to address loss-to-follow up at the final phase using multiple imputation, however these models may still be biased if this is informative censoring, whereby the observed outcome itself predicts missingness.(42) A post hoc power calculation showed reasonable power to detect moderate to large differences between participants with and without sSCD. But the sample size and incidence of new CIND and dementia meant we could not exclude a modest increased risk.

Conclusions

Subjective cognitive decline is modestly associated with objective cognitive decline, but these processes appear to have largely distinct underlying mechanisms. Our data provide reassuring evidence that subjects with sSCD but without objective impairments, are not at increased risk of later CIND or dementia.

List of abbreviations:

FCD: Functional Cognitive Disorder

For peer review only - http://bmjopen.bmj.com/site/about/guidelines.xhtml

475 MCI: Mild Cognitive Impairment

476 SCD: Subjective Cognitive Decline

477 sSCD: simple Subjective Cognitive Decline (the subjective report of cognitive decline, with no other qualifiers such as score on objective tests or absence of comorbidities).

For peer review only

For peer review only - http://bmjopen.bmj.com/site/about/guidelines.xhtml

DECLARATIONS

Ethical approval and consent to participate

All participants gave informed consent. Ethical approval was provided by Gwent Research Ethics Committee (01/69).

Consent for publication

Not applicable.

Availability of data and materials

The datasets analysed during the current study are not publicly available due the sensitive information contained, and the nature of the consent given by the participants. The data that support the findings are available on reasonable request.

Competing interests

None of the authors declare any competing interests.

Funding

CaPS was funded by the Medical Research Council (grant G9824960-E01/1), and phase 5 was funded by a grant from the Alzheimer's Society.

Author Contributions

MF, AB, JG and YBS contributed to conception and design of the study. JG led the data collection. MF and AB established the clinical diagnoses. HB, YBS and EC planned the analyses. HB analysed the data and wrote the first draft. All authors read and approved the final manuscript.

Acknowledgements

HB is a National Institute for Health Research (NIHR) academic clinical lecturer. YBS is partially supported by the NIHR Applied Research Collaboration West (NIHR ARC West). JG is partially supported by MRC grant MR/T0333771. The views expressed in this article are those of the author(s) and not necessarily those of the NIHR or the Department of Health and Social Care.

For peer review only - http://bmjopen.bmj.com/site/about/guidelines.xhtml

Table 1: Relationship between change in objective cognition performance (Phase 3 to 5) and self-reported simple Subjective Cognitive Decline (sSCD) (phase 5)

	MEAN (SD)			P-VALUE	ADJUSTED R-SQUARED*
**DECLINE IN COGNITIVE TEST SCORE (ANNUAL RATE)**	No subjective cognitive decline	Subjective cognitive decline	Difference (95% CI)		
WHOLE SAMPLE					
**CAMCOG**	0.17 (0.63)	0.35 (0.91)	0.18 (0.8, 0.27)	<0.001	0.01
**AH4**	0.08 (0.61)	0.26 (0.66)	0.18 (0.09, 0.27)	<0.001	0.02
EXCLUDING THOSE WITH DEMENTIA AT PHASE 5					
**CAMCOG**	0.13 (0.55)	0.17 (0.49)	0.04 (-0.04, 0.11)	0.36	<0.001
**AH4**	0.07 (0.59)	0.22 (0.62)	0.16 (0.07, 0.24)	<0.001	0.01

*The proportion of variance in the dependent variable, that can be predicted by the independent variable

For peer review only - http://bmjopen.bmj.com/site/about/guidelines.xhtml

Table 2: The diagnostic utility of simple subjective cognitive decline (sSCD), for objective cognitive performance

	OBJECTIVE COGNITIVE DECLINE,* N(%)	NO OBJECTIVE COGNITIVE DECLINE, N(%)	P-VALUE	SENSITIVITY (95% CONFIDENCE INTERVAL)	SPECIFICITY (95% CONFIDENCE INTERVAL)
**WHOLE SAMPLE**					
	**CAMCOG +VE**	**CAMCOG -VE**			
**SSCD**	90 (9)	201 (21)	0.03	36 (30-42)	72 (68-75)
**NO SSCD**	163 (17)	515 (53)			
	**AH4 +VE**	**AH4-VE**			
**SSCD**	71 (8)	205 (22)	<0.001	44 (36-52)	73 (70-76)
**NO SSCD**	91 (10)	558 (60)			
	**CAMCOG OR AH4 +VE**	**CAMCOG OR AH4 -VE**			
**SSCD**	132 (14)	159 (16)	<0.001	38 (33-43)	74 (71-78)
**NO SSCD**	216 (22)	462 (48)			
**EXCLUDING DEMENTIA**					
	**CAMCOG +VE**	**CAMCOG -VE**			
**SSCD**	67 (7)	199 (21)	0.36	31 (25-38)	72 (69-75)
**NOSSCD**	148 (16)	513 (55)			
	**AH4 +VE**	**AH4-VE**			
**SSCD**	61 (7)	196 (22)	<0.001	42 (34-51)	74 (71-77)
**NO SSCD**	83 (9)	552 (62)			
	**CAMCOG OR AH4 +VE**	**CAMCOG OR AH4 -VE**			
**SSCD**	109 (12)	157 (17)	0.002	35 (30-41)	75 (71-78)
**NO SSCD**	201 (22)	460 (50)			

* Continuous scoring converted to a binary variable, +ve/-ve indicates whether a participant had declined more/less than 1 standard deviation from the mean rate, calculated amongst men without CIND nor dementia, between Phases 3 to 5.

sSCD = simple Subjective Cognitive decline; CAMCOG = Cambridge Cognitive Examination; AH4 = Alice Heim 4 test.

For peer review only - http://bmjopen.bmj.com/site/about/guidelines.xhtml

For peer review only

For peer review only - http://bmjopen.bmj.com/site/about/guidelines.xhtml

Table 3: Simple subjective cognitive decline and cognitive internal inconsistency, amongst people without objective cognitive decline (at phase 5). N=619.

MEN WITH NO OBJECTIVE COGNITIVE DECLINE, PHASE 5	SUBJECTIVE REPORT OF COGNITION		
**RIVERMEAD STORY RECALL TASK**	No sSCD, mean (SD)	sSCD, mean (SD)	P-value
**IMMEDIATE RECALL "IR"**	6.15 (2.82)	5.55 (2.59)	0.02
**DELAYED RECALL "DR"**	5.29 (2.78)	4.73 (2.59)	0.03
**IR-DR**	0.84 (1.45)	0.83 (1.41)	0.92
**RETENTION: DR/IR**	0.86 (0.37)	0.86 (0.35)	0.58
**RELATIVE DIFFERENCE: (IR-DR)/IR**	0.12 (0.37)	0.14 (0.35)	0.58
**CONSISTENCY OF RECALL ABILITY**	n (%)		Chi-squared P-value
**"CONSISTENT" (DR =/< IR)**	370 (80.8)	130 (82.3)	0.68
**"INCONSISTENT" (DR > IR)**	88 (19.2)	28 (17.7)	

sSCD = simple Subjective Cognitive decline

review only

For peer review only - http://bmjopen.bmj.com/site/about/guidelines.xhtml

Table 4: Possible risk factors (phases 2 to 4) for simple Subjective Cognitive Decline (sSCD) (phase 5)

	ADJUSTED OR (95% CI) (n=601)	ADJUSTED OR (95% CI) EXCLUDING DEMENTIA (N=580)
AGE (YEARS)	1.12 (1.06, 1.17)	1.11 (1.05, 1.17)
SOCIAL CLASS (MANUAL/NON-MANUAL)	0.99 (0.65, 1.51)	0.95 (0.62, 1.46)
PREMORBID IQ (X 0.1)	1.06 (0.84, 1.33)	1.05 (0.83, 1.32)
HIGH ALCOHOL USE (BINARY)	1.07 (0.63, 1.83)	1.20 (0.70, 2.07)
TEETOTAL (BINARY)	0.64 (0.31, 1.32)	0.60 (0.29, 1.27)
PROBABLE ISCHAEMIC HEART DISEASE (BINARY)	0.98 (0.59, 1.62)	1.03 (0.61, 1.72)
NUMBER OF VASCULAR MEDICATIONS	1.18 (0.92, 1.50)	1.20 (0.93, 1.54)
SMOKING (BINARY)	0.89 (0.60, 1.34)	0.85 (0.56, 1.30)
WAIST-HIP RATIO (X10)	0.93 (0.66, 1.31)	0.89 (0.63, 1.27)
POOR SLEEP	1.52 (1.19, 1.94)	1.55 (1.21, 1.99)
SLEEP-DISORDERED BREATHING	1.13 (0.91, 1.40)	1.12 (0.90, 1.40)
MOOD SYMPTOMS (GHQ SCORE: CHRONIC)*	1.27 (1.01, 1.59)	1.23 (0.97, 1.55)
TRAIT ANXIETY (STAI)*	1.38 (1.09, 1.74)	1.39 (1.09, 1.76)
RATE OF DECLINE CAMCOG	1.18 (0.83, 1.69)	1.18 (0.72, 1.95)
RATE OF DECLINE AH4	1.33 (0.95, 1.85)	1.36 (0.97, 1.92)

*standardised

For peer review only - http://bmjopen.bmj.com/site/about/guidelines.xhtml

Table 5: sSCD does not predict future cognitive outcomes (new CIND or dementia)

**PREDICTOR AT PHASE 5: SSCD**			
**OUTCOME AT PHASE 7**	CIND or dementia	Dementia	Decline in CAMCOG between phases 5 and 7*
**PARTICIPANTS**	Excluding those with CIND or dementia at phase 5	Excluding those with dementia at phase 5	Excluding those with dementia at phase 5
**MODEL**	Odds ratio (95% CI)	Odds ratio (95% CI)	Linear coefficient (95% CI)
**COMPLETE CASE ANALYSIS**	0.92 (0.49, 1.73)	1.35 (0.61, 2.99)	0.01 (-0.13, 0.15)
**MULTIPLE IMPUTATION**	0.98 (0.56, 1.74)	1.18 (0.53, 2.61)	-0.02 (-0.19, 0.15)

All models controlled for: age, CAMCOG score at phase 5.

*The CAMCOG analysis also controlled for a dummy variable indicating CAMCOG points lost for hearing, vision, or manual dexterity at phase 7.

For peer review only - http://bmjopen.bmj.com/site/about/guidelines.xhtml

References:

1. McWhirter L, Ritchie C, Stone J, Carson A. Functional cognitive disorders: a systematic review. The Lancet Psychiatry. 2020;7(2):191-207.
2. Burmester B, Leathem J, Merrick P. Subjective Cognitive Complaints and Objective Cognitive Function in Aging: A Systematic Review and Meta-Analysis of Recent Cross-Sectional Findings. Neuropsychology Review. 2016;26(4):376-93.
3. Crumley JJ, Stetler CA, Horhota M. Examining the relationship between subjective and objective memory performance in older adults: a meta-analysis. Psychol Aging. 2014;29(2):250-63.
4. Mol ME, van Boxtel MP, Willems D, Jolles J. Do subjective memory complaints predict cognitive dysfunction over time? A six-year follow-up of the Maastricht Aging Study. Int J Geriatr Psychiatry. 2006;21(5):432-41.
5. Jessen F, Amariglio RE, van Boxtel M, Breteler M, Ceccaldi M, Chételat G, et al. A conceptual framework for research on subjective cognitive decline in preclinical Alzheimer's disease. Alzheimer's & Dementia. 2014;10(6):844-52.
6. Slot RER, Sikkes SAM, Berkhof J, Brodaty H, Buckley R, Cavedo E, et al. Subjective cognitive decline and rates of incident Alzheimer's disease and non–Alzheimer's disease dementia. Alzheimer's & Dementia. 2019;15(3):465-76.
7. Pavisic IM, Lu K, Keuss SE, James S-N, Lane CA, Parker TD, et al. Subjective cognitive complaints at age 70: associations with amyloid and mental health. Journal of Neurology, Neurosurgery & Psychiatry. 2021;92(11):1215.
8. Brailean A, Steptoe A, Batty GD, Zaninotto P, Llewellyn DJ. Are subjective memory complaints indicative of objective cognitive decline or depressive symptoms? Findings from the English Longitudinal Study of Ageing. J Psychiatr Res. 2019;110:143-51.
9. Jessen F, Amariglio RE, Buckley RF, van der Flier WM, Han Y, Molinuevo JL, et al. The characterisation of subjective cognitive decline. The Lancet Neurology. 2020.
10. Visser PJ, Verhey F, Knol DL, Scheltens P, Wahlund L-O, Freund-Levi Y, et al. Prevalence and prognostic value of CSF markers of Alzheimer's disease pathology in patients with subjective cognitive impairment or mild cognitive impairment in the DESCRIPA study: a prospective cohort study. The Lancet Neurology. 2009;8(7):619-27.
11. Eckerström M, Göthlin M, Rolstad S, Hessen E, Eckerström C, Nordlund A, et al. Longitudinal evaluation of criteria for subjective cognitive decline and preclinical Alzheimer's disease in a memory clinic sample. Alzheimer's & Dementia: Diagnosis, Assessment & Disease Monitoring. 2017;8:96-107.
12. Ball HA, McWhirter L, Ballard C, Bhome R, Blackburn DJ, Edwards MJ, et al. Functional cognitive disorder: dementia's blind spot. Brain. 2020.
13. Ganguli M, Jia Y, Hughes TF, Snitz BE, Chang C-CH, Berman SB, et al. Mild Cognitive Impairment that Does Not Progress to Dementia: A Population-Based Study. J Am Geriatr Soc. 2019;67(2):232-8.
14. Pennington C, Ball H, Swirski M. Functional Cognitive Disorder: Diagnostic Challenges and Future Directions. Diagnostics (Basel). 2019;9(4).
15. Banks J, Blundell R, Calderwood L. Wave 1- Health, Wealth and lifestyles of the older population in England: ELSA 2002. Marmot M, Banks J, Blundell R, Lessor C, Nazroo J, editors2002.
16. Mol ME, van Boxtel MP, Willems D, Verhey FR, Jolles J. Subjective forgetfulness is associated with lower quality of life in middle-aged and young-old individuals: a 9-year follow-up in older participants from the Maastricht Aging Study. Aging Ment Health. 2009;13(5):699-705.
17. Miller MA, Wright H, Ji C, Cappuccio FP. Cross-sectional study of sleep quantity and quality and amnestic and non-amnestic cognitive function in an ageing population: the English Longitudinal Study of Ageing (ELSA). PLoS One. 2014;9(6):e100991.

For peer review only - http://bmjopen.bmj.com/site/about/guidelines.xhtml

18. Fish M, Bayer AJ, Gallacher JE, Bell T, Pickering J, Pedro S, et al. Prevalence and pattern of cognitive impairment in a community cohort of men in South Wales: methodology and findings from the Caerphilly Prospective Study. Neuroepidemiology. 2008;30(1):25-33.
19. Creavin ST, Gallacher J, Bayer A, Fish M, Ebrahim S, Ben-Shlomo Y. Metabolic syndrome, diabetes, poor cognition, and dementia in the Caerphilly prospective study. J Alzheimers Dis. 2012;28(4):931-9.
20. Elwood PC, Pickering J, Bayer A, Gallacher JEJ. Vascular disease and cognitive function in older men in the Caerphilly cohort. Age and Ageing. 2002;31(1):43-8.
21. Roth M, Tym E, Mountjoy CQ, Huppert FA, Hendrie H, Verma S, et al. CAMDEX. A standardised instrument for the diagnosis of mental disorder in the elderly with special reference to the early detection of dementia. Br J Psychiatry. 1986;149:698-709.
22. Heim AW. Manual for the Group test of General Intelligence AH4. London, England: National Foundation for Educational Research.; 1955.
23. Wilson BA, Cockburn J, Baddeley AD. The Rivermead behavioural memory test. London: Thames Valley Test Company; 1985.
24. Jorm AF, Jacomb PA. The Informant Questionnaire on Cognitive Decline in the Elderly (IQCODE): socio-demographic correlates, reliability, validity and some norms. Psychol Med. 1989;19(4):1015-22.
25. von Elm E, Altman DG, Egger M, Pocock SJ, Gøtzsche PC, Vandenbroucke JP. Strengthening the Reporting of Observational Studies in Epidemiology (STROBE) statement: guidelines for reporting observational studies. Bmj. 2007;335(7624):806-8.
26. Tombaugh TN. The Test of Memory Malingering (TOMM): Normative data from cognitively intact and cognitively impaired individuals. Psychological Assessment. 1997;9(3):260.
27. Nelson HE. National adult reading test (NART) : for the assessment of premorbid intelligence in patients with dementia : test manual. Windsor, Berks: NFER-Nelson; 1982.
28. Rehm J, Hasan OSM, Black SE, Shield KD, Schwarzinger M. Alcohol use and dementia: a systematic scoping review. Alzheimers Res Ther. 2019;11(1):1-.
29. Dziak JJ, Dierker LC, Abar B. The Interpretation of Statistical Power after the Data have been Gathered. Curr Psychol. 2020;39(3):870-7.
30. Ogliari G, Turner Z, Khalique J, Gordon AL, Gladman JRF, Chadborn NH. Ethnic disparity in access to the memory assessment service between South Asian and white British older adults in the United Kingdom: A cohort study. International Journal of Geriatric Psychiatry. 2020;35(5):507-15.
31. Liew TM, Yap P, Ng TP, Mahendran R, Kua EH, Feng L. Symptom clusters of subjective cognitive decline amongst cognitively normal older persons and their utilities in predicting objective cognitive performance: structural equation modelling. Eur J Neurol. 2019;26(9):1153-60.
32. Cosentino S, Devanand D, Gurland B. A Link between Subjective Perceptions of Memory and Physical Function: Implications for Subjective Cognitive Decline. Journal of Alzheimer's disease : JAD. 2018;61(4):1387-98.
33. Suo C, Gates N, Singh MF, Saigal N, Wilson GC, Meiklejohn J, et al. Midlife managerial experience is linked to late life hippocampal morphology and function. Brain imaging and behavior. 2017;11(2):333-45.
34. Goldstein FC, Loring DW, Thomas T, Saleh S, Hajjar I. Recognition Memory Performance as a Cognitive Marker of Prodromal Alzheimer's Disease. Journal of Alzheimer's disease : JAD. 2019;72(2):507-14.
35. Russo MJ, Campos J, Vazquez S, Sevlever G, Allegri RF. Adding Recognition Discriminability Index to the Delayed Recall Is Useful to Predict Conversion from Mild Cognitive Impairment to Alzheimer's Disease in the Alzheimer's Disease Neuroimaging Initiative. Front Aging Neurosci. 2017;9:46.
36. Oda H, Yamamoto Y, Maeda K. Neuropsychological profile of dementia with Lewy bodies. Psychogeriatrics. 2009;9(2):85-90.

For peer review only - http://bmjopen.bmj.com/site/about/guidelines.xhtml

37. Teodoro T, Edwards MJ, Isaacs JD. A unifying theory for cognitive abnormalities in functional neurological disorders, fibromyalgia and chronic fatigue syndrome: systematic review. Journal of Neurology Neurosurgery and Psychiatry. 2018;89(12):1308-19.
38. de Winter JCF, Dodou D, Hancock PA. On the paradoxical decrease of self-reported cognitive failures with age. Ergonomics. 2015;58(9):1471-86.
39. Bhome R, McWilliams A, Huntley JD, Fleming SM, Howard RJ. Metacognition in functional cognitive disorder- a potential mechanism and treatment target. Cogn Neuropsychiatry. 2019;24(5):311-21.
40. Chin J, Oh KJ, Seo SW, Na DL. Are depressive symptomatology and self-focused attention associated with subjective memory impairment in older adults? Int Psychogeriatr. 2014;26(4):573-80.
41. Schweizer S, Kievit RA, Emery T, Cam CAN, Henson RN. Symptoms of depression in a large healthy population cohort are related to subjective memory complaints and memory performance in negative contexts. Psychological medicine. 2018;48(1):104-14.
42. Dufouil C, Brayne C, Clayton D. Analysis of longitudinal studies with death and drop-out: a case study. Stat Med. 2004;23(14):2215-26.

For peer review only

For peer review only - http://bmjopen.bmj.com/site/about/guidelines.xhtml

Supplemental material for "Predictors and prognosis of population-based subjective cognitive decline: Longitudinal evidence from the Caerphilly Prospective Study (CaPS)"

Contents

Items derived from The Wisconsin Sleep Scale 1

Supplemental Figure 1: Caerphilly Prospective Study (CaPS) flowchart 2

Supplemental Figure 2: Histogram displaying delayed recall minus immediate recall ("internal inconsistency") at phase 5, according to presence or absence of simple Subjective Cognitive Decline (sSCD) 3

Supplemental Table 1: Relative frequency of possible risk variables, amongst people with and without simple Subjective Cognitive Decline (sSCD) (Phase 5) 4

Supplemental Table 2: Missingness by variable, as a percentage of n=1225 participants at phase 5 6

Supplemental table 3: possible risk factors (phases 2 to 4) for simple Subjective Cognitive Decline (phase 5), including unadjusted and imputed models 8

Items derived from The Wisconsin Sleep Scale

The Wisconsin sleep scale is mainly composed of items scored on a 1-5 point scale. These items were separated into two groups, then summed and standardized: i) *Experiencing poor sleep*, including: difficulty initiating sleep; waking overnight; difficulty getting back to sleep; not feeling rested during the day; daytime sleepiness; dozing easily in the daytime; use of caffeine or other stimulants to stay awake; and napping during the day. This comprised 8 items, (alpha 0.77). Minimum and maximum scores prior to standardisation were 8 and 39. ii) Breathing-disordered Sleep, including: frequency of snoring; loudness of snoring; awareness of snoring; making gasp/ choke/ snort noises during sleep; waking with these noises; and frequency of momentary periods in sleep where you breathe abnormally. This comprised 6 items (alpha 0.77). Minimum and maximum scores prior to standardisation were 6 and 30. Note this approach excluded items on the Wisconsin questionnaire that were either scored on a substantially different scale, or assessing other aspects of sleep: how awake you feel today; restless legs/ twitches/ kicking during sleep; nasal congestion interrupting sleep; sleep latency; reported duration of sleep (weekdays and weekends); use of sleeping tablets; and allergies causing nasal congestion.

For peer review only

Supplemental Figure 1: Caerphilly Prospective Study (CaPS) flowchart

References: (1, 2, 3)

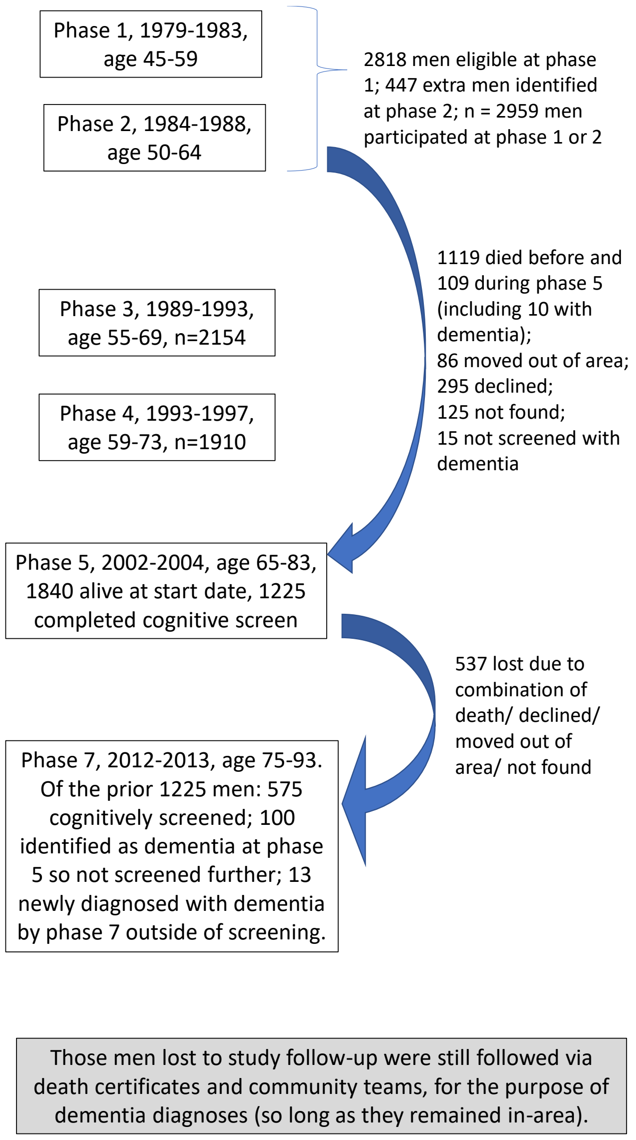

References:

1. Fish M, Bayer AJ, Gallacher JE, Bell T, Pickering J, Pedro S, et al. Prevalence and pattern of cognitive impairment in a community cohort of men in South Wales: methodology and findings from the Caerphilly Prospective Study. Neuroepidemiology. 2008;30(1):25-33.
2. Creavin ST, Gallacher J, Bayer A, Fish M, Ebrahim S, Ben-Shlomo Y. Metabolic syndrome, diabetes, poor cognition, and dementia in the Caerphilly prospective study. J Alzheimers Dis. 2012;28(4):931-9.
3. Elwood PC, Pickering J, Bayer A, Gallacher JEJ. Vascular disease and cognitive function in older men in the Caerphilly cohort. Age and Ageing. 2002;31(1):43-8.

For peer review only - http://bmjopen.bmj.com/site/about/guidelines.xhtml

Supplemental Figure 2: Histogram displaying delayed recall minus immediate recall (“internal inconsistency”) at phase 5, according to presence or absence of simple Subjective Cognitive Decline (sSCD)

This analysis included only those men with no objective decline in measured cognition since Phase 3.

Score greater than 0 is “inconsistent”. Note how roughly one fifth of men had this marker of internal inconsistency (regardless of subjective decline status).

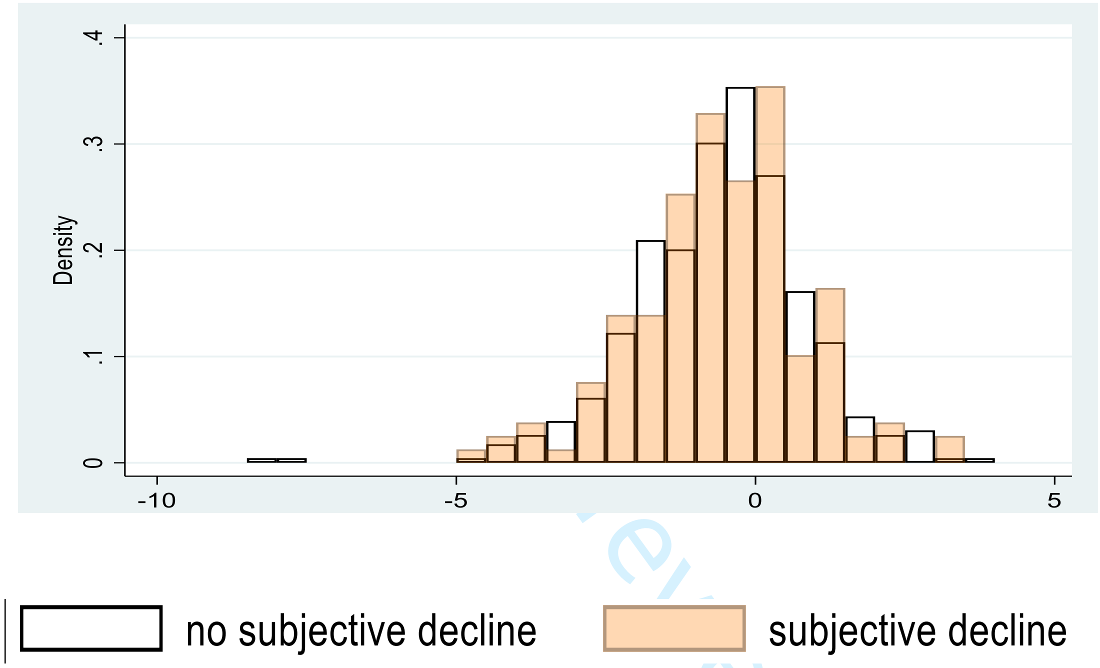

Supplemental Table 1: Relative frequency of possible risk variables, amongst people with and without simple Subjective Cognitive Decline (sSCD) (Phase 5)

	TOTAL N	NO SUBJECTIVE COGNITIVE DECLINE, N	SUBJECTIVE COGNITIVE DECLINE, N
TOTAL: N	1225	739	326
CIND (PHASE 5)	192	79	71
DEMENTIA (PHASE 5)	75	19	31
MANUAL SOCIAL CLASS	720	417	192
LEFT SCHOOL BEFORE 14	507	286	149
TEETOTAL (AT ANY PHASE 3-5)	120	63	37
CUMULATIVE HIGH ALCOHOL INTAKE	182	110	47
CURRENT SMOKER (ANY OF PHASE 3-5)	452	261	122
PROBABLE ISCHAEMIC HEART DISEASE	253	131	84
ANTIDEPRESSANT MEDICATION (BINARY)	15	6	8
	**ALL**	**NO SUBJECTIVE COGNITIVE DECLINE, MEAN (SD)**	**SUBJECTIVE COGNITIVE DECLINE, MEAN (SD)**
AGE (YEARS)	73.0 (4.2)	72.5 (4.0)	73.6 (4.2)
PREMORBID IQ	107.1 (9.9)	108.0 (9.9)	107.0 (9.7)
BODY MASS INDEX	26.9 (3.5)	27.0 (3.5)	26.8 (3.3)
WAIST-HIP RATIO	0.93 (0.06)	0.93 (0.06)	0.93 (0.06)
NUMBER OF MEDICATIONS FOR VASCULAR RISK	0.40 (0.83)	0.32 (0.71)	0.54 (1.05)
EXPERIENCING POOR SLEEP	-0.05 (0.95)	-0.23 (0.87)	0.33 (0.97)
BREATHING-DISORDERED SLEEP	0.02 (0.93)	-0.05 (0.88)	0.22 (0.98)
GHQ (MOOD) CHRONIC SCORING	9.0 (6.3)	7.8 (5.7)	11.3 (6.7)
STAI (TRAIT ANXIETY)	35.9 (8.9)	34.3 (8.5)	38.8 (9.3)
CAMCOG RATE OF CHANGE PER YEAR	-0.29 (0.96)	-0.17 (0.63)	-0.35 (0.91)

**AH4 RATE OF CHANGE PER YEAR**	-0.15 (0.64)	-0.08 (0.61)	-0.26 (0.66)

CIND = cognitive impairment, not depression; QGH = general health questionnaire (a measure of mood symptoms); STAI = Spielberger State-Trait Anxiety Inventory; CAMCOG = Cambridge Cognitive Examination; AH4= Alice Heim 4 test. *For missingness data, see Supplementary Table 1.*

Note where "no subjective cognitive decline" and "subjective cognitive decline" columns do not add up to the total, this is due to missing data. This data was missing 160/1225 (13%) for the whole cohort, 42/192 (22%) for CIND and 25/75 (33%) for dementia.

For peer review only

Supplemental Table 2: Missingness by variable, as a percentage of n=1225 participants at phase 5

VARIABLE	PHASE OF DATA COLLECTION	% MISSING
Age	Baseline	(none)
Sex	Baseline	(none, all men)
Manual social class	Baseline	4.9
Left school at 14	Baseline	10.3
STAI	2	5.3
Probable ischaemic heart disease	3	5.6
Antidepressant medication (binary)	3	Missingness data not available
Premorbid IQ	3	12.1
Body mass index	3	10.5
Waist hip ratio	3	8.2
Experiencing poor sleep	3	9.1
Sleep-disordered breathing	3	34.0
Mood symptoms (GHQ)	3	17.8
CAMCOG	3	11.6
AH4	3	12.2
CAMCOG	4	15.4
AHR	4	16.2
Teetotal	3-5	4.8
Cumulative high alcohol intake	3-5	4.8
Smoker	3-5	4.7
Number of medications for vascular risk	3	Missingness data not available
Subjective Cognitive Decline	5	13.1
CAMCOG	5	0.4
AH4	5	6.9
Immediate recall	5	2.5
Delayed recall	5	2.5
CIND or dementia	5	0.2
CAMCOG (excluding men who had dementia by phase 5)	7	56.0

For peer review only

CIND or dementia (excluding men who had dementia by phase 5)	7	53.0

For peer review only

Supplemental table 3: possible risk factors (phases 2 to 4) for simple Subjective Cognitive Decline (phase 5), including unadjusted and imputed models

	n FOR UNADJUSTED OR	UNADJUSTED OR (95% CI)	ADJUSTED OR (95% CI) (n=601)	ADJUSTED OR (95% CI), MICE (N=1225)	ADJUSTED OR (95% CI) EXCLUDING DEMENTIA (N=580)
**AGE (YEARS)**	1065	1.07 (1.03, 1.10)	1.12 (1.06, 1.17)	1.08 (1.04-1.12)	1.11 (1.05, 1.17)
**SOCIAL CLASS (MANUAL/NON-MANUAL)**	1020	1.10 (0.84, 1.45)	0.99 (0.65, 1.51)	1.07 (0.75-1.51)	0.95 (0.62, 1.46)
**PREMORBID IQ (X 0.1)**	956	0.90 (0.78, 1.03)	1.06 (0.84, 1.33)	1.09 (0.90-1.32)	1.05 (0.83, 1.32)
**HIGH ALCOHOL USE (BINARY)**	1022	0.96 (0.66, 1.39)	1.07 (0.63, 1.83)	1.08 (0.71-1.64)	1.20 (0.70, 2.07)
**TEETOTAL (BINARY)**	1022	1.37 (0.89, 2.11)	0.64 (0.31, 1.32)	1.14 (0.69-1.90)	0.60 (0.29, 1.27)
**PROBABLE ISCHAEMIC HEART DISEASE (BINARY)**	1015	1.62 (1.18, 2.22)	0.98 (0.59, 1.62)	0.94 (0.65-1.36)	1.03 (0.61, 1.72)
**NUMBER OF VASCULAR MEDICATIONS**	1065	1.33 (1.15, 1.55)	1.18 (0.92, 1.50)	1.10 (0.92-1.31)	1.20 (0.93, 1.54)
**SMOKING (BINARY)**	1022	1.10 (0.83, 1.44)	0.89 (0.60, 1.34)	1.10 (0.80-1.51)	0.85 (0.56, 1.30)
**WAIST-HIP RATIO (X10)**	990	1.19 (0.94, 1.50)	0.93 (0.66, 1.31)	1.07 (0.84-1.38)	0.89 (0.63, 1.27)
**POOR SLEEP**	983	1.91 (1.64, 2.22)	1.52 (1.19, 1.94)	1.48 (1.22-1.79)	1.55 (1.21, 1.99)
**SLEEP-DISORDERED BREATHING**	727	1.39 (1.17, 1.65)	1.13 (0.91, 1.40)	1.16 (0.95-1.42)	1.12 (0.90, 1.40)
**MOOD SYMPTOMS (GHQ SCORE: CHRONIC)***	886	1.78 (1.53, 2.06)	1.27 (1.01, 1.59)	1.38 (1.13-1.69)	1.23 (0.97, 1.55)
**TRAIT ANXIETY (STAI)***	1019	1.66 (1.44, 1.91)	1.38 (1.09, 1.74)	1.31 (1.10-1.57)	1.39 (1.09, 1.76)
**RATE OF DECLINE CAMCOG**	969	1.37 (1.13, 1.66)	1.18 (0.83, 1.69)	1.15 (0.95-1.40)	1.18 (0.72, 1.95)
**RATE OF DECLINE AH4**	925	1.59 (1.26, 1.99)	1.33 (0.95, 1.85)	1.50 (1.16-1.95)	1.36 (0.97, 1.92)

MICE = multiple imputation with chained equations

Reporting checklist for cohort study.

Based on the STROBE cohort guidelines.

Instructions to authors

Complete this checklist by entering the page numbers from your manuscript where readers will find each of the items listed below.

Your article may not currently address all the items on the checklist. Please modify your text to include the missing information. If you are certain that an item does not apply, please write "n/a" and provide a short explanation.

Upload your completed checklist as an extra file when you submit to a journal.

In your methods section, say that you used the STROBE cohortreporting guidelines, and cite them as:

von Elm E, Altman DG, Egger M, Pocock SJ, Gotzsche PC, Vandenbroucke JP. The Strengthening the Reporting of Observational Studies in Epidemiology (STROBE) Statement: guidelines for reporting observational studies.

For peer review only

		Reporting Item	Page Number
**Title and abstract**			
Title	#1a	Indicate the study’s design with a commonly used term in the title or the abstract	1

Abstract	#1b	Provide in the abstract an informative and balanced summary of what was done and what was found	2
**Introduction**			
Background / rationale	#2	Explain the scientific background and rationale for the investigation being reported	4
Objectives	#3	State specific objectives, including any prespecified hypotheses	6
**Methods**			
Study design	#4	Present key elements of study design early in the paper	6
Setting	#5	Describe the setting, locations, and relevant dates, including periods of recruitment, exposure, follow-up, and data collection	6
Eligibility criteria	#6a	Give the eligibility criteria, and the sources and methods of selection of participants. Describe methods of follow-up.	6
Eligibility criteria	#6b	For matched studies, give matching criteria and number of exposed and unexposed	N/A
Variables	#7	Clearly define all outcomes, exposures, predictors, potential confounders, and effect modifiers. Give diagnostic criteria, if applicable	7-8
Data sources / measurement	#8	For each variable of interest give sources of data and details of methods of assessment (measurement). Describe	7-8

For peer review only

		comparability of assessment methods if there is more than one group. Give information separately for for exposed and unexposed groups if applicable.	
Bias	#9	Describe any efforts to address potential sources of bias	8-9
Study size	#10	Explain how the study size was arrived at	6
Quantitative variables	#11	Explain how quantitative variables were handled in the analyses. If applicable, describe which groupings were chosen, and why	8-9
Statistical methods	#12a	Describe all statistical methods, including those used to control for confounding	
8-9			
Statistical methods	#12b	Describe any methods used to examine subgroups and interactions	23
Statistical methods	#12c	Explain how missing data were addressed	8
Statistical methods	#12d	If applicable, explain how loss to follow-up was addressed	8
Statistical methods	#12e	Describe any sensitivity analyses	
9			

Results

For peer review only

Participants	#13a	Report numbers of individuals at each stage of study—eg numbers potentially eligible, examined for eligibility, confirmed eligible, included in the study, completing follow-up, and analysed. Give information separately for for exposed and unexposed groups if applicable.	Figure 1
Participants	#13b	Give reasons for non-participation at each stage	Figure 1
Participants	#13c	Consider use of a flow diagram	
Figure 1			
Descriptive data	#14a	Give characteristics of study participants (eg demographic, clinical, social) and information on exposures and potential confounders. Give information separately for exposed and unexposed groups if applicable.	Table 1
Descriptive data	#14b	Indicate number of participants with missing data for each variable of interest - Supplementary Table 1	
Descriptive data	#14c	Summarise follow-up time (eg, average and total amount)	
Figure 1			
Outcome data	#15	Report numbers of outcome events or summary measures over time. Give information separately for exposed and unexposed groups if applicable.	
Page 11			

For peer review only

Main results	#16a	Give unadjusted estimates and, if applicable, confounder-adjusted estimates and their precision (eg, 95% confidence interval). Make clear which confounders were adjusted for and why they were included	Tables
Main results	#16b	Report category boundaries when continuous variables were categorized	7
Main results	#16c	If relevant, consider translating estimates of relative risk into absolute risk for a meaningful time period	
N/A			
Other analyses	#17	Report other analyses done—eg analyses of subgroups and interactions, and sensitivity analyses	Tables
**Discussion**			
Key results	#18	Summarise key results with reference to study objectives	12
Limitations	#19	Discuss limitations of the study, taking into account sources of potential bias or imprecision. Discuss both direction and magnitude of any potential bias.	14-15
Interpretation	#20	Give a cautious overall interpretation considering objectives, limitations, multiplicity of analyses, results from similar studies, and other relevant evidence.	12-16
Generalisability	#21	Discuss the generalisability (external validity) of the study results	15

For peer review only

Other Information

Funding	#22	Give the source of funding and the role of the funders for the present study and, if applicable, for the original study on which the present article is based	17

None The STROBE checklist is distributed under the terms of the Creative Commons Attribution License CC-BY. This checklist can be completed online using https://www.goodreports.org/, a tool made by the EQUATOR Network in collaboration with Penelope.ai

For peer review only
